# Supplementary material for: The Discovery of Antibacterial Cembranoids from the Soft Coral Lobophytum crassum by DeepSAT Analysis
Source: Mar Drugs. 2025 Dec 6;23(12):468. doi: 10.3390/md23120468 (PMC12734846; doi:10.3390/md23120468)
Supplement: Supplementary file 1 [file marinedrugs-23-00468-s001.zip › marinedrugs-4010385-supplementary.pdf]

Supporting Information for

The discovery of antibacterial cembranoids from the Soft Coral *Lobophytum crassum* by DeepSAT analysis

Bing Wu[1,2], Li-Gong Yao[2], Ming-Zhi Su[2], Gui-Ge Hou[1,*], Song-Wei Li[3,*], and Yue-Wei Guo[2,3*]

[1] *School of Pharmacy, Binzhou Medical University, Yantai 264003, China*

[2] *Shandong Laboratory of Yantai Drug Discovery, Bohai Rim Advanced Research Institute for Drug Dis-covery, Yantai 264117, China*

[3] *School of Medicine, Shanghai University, Shanghai 200444, China*

* Correspondence: guigehou@163.com (G.-G.H.); songweili@shu.edu.cn (S.-W.L.); ywguo@simm.ac.cn (Y.-W.G.)

CONTENT

Figure S1. ^{1}H NMR spectrum (600 MHz) of compound **1** in $CDCl_3$..5
Figure S2. ^{13}C NMR spectrum (600 MHz) of compound **1** in $CDCl_3$. 5
Figure S3. DEPT spectrum (150 MHz) of compound **1** in $CDCl_3$..6
Figure S4. ^{1}H-^{1}H COSY spectrum (600 MHz) of compound 1 in $CDCl_3$...............................6
Figure S5. HSQC spectrum (600 MHz) of compound **1** in $CDCl_3$. 7
Figure S6. HMBC spectrum (600 MHz) of compound **1** in $CDCl_3$. 7
Figure S7. NOESY spectrum (600 MHz) of compound **1** in $CDCl_3$..8
Figure S8. HR-ESI-MS spectrum of compound **1**. 8
Figure S9. IR spectrum of compound **1**. 9
Figure S10. DP4+ results of compound **1** (Isomer 1: 2*R**, 7*S**, 8*S**, 11*S**; Isomer 2: 2*S**, 7*S**, 8*S**, 11*S**). 9
Figure S11. UV and CD spectrum of compound **1**..10
Figure S12. ^{1}H NMR spectrum (600 MHz) of compound **2** in $CDCl_3$...................................10
Figure S13. ^{13}C NMR spectrum (600 MHz) of compound **2** in $CDCl_3$. 11
Figure S14. DEPT spectrum (150 MHz) of compound **2** in $CDCl_3$......................................11
Figure S15. ^{1}H-^{1}H COSY spectrum (600 MHz) of compound **2** in $CDCl_3$............................12
Figure S16. HSQC spectrum (600 MHz) of compound **2** in $CDCl_3$. 12
Figure S17. HMBC spectrum (600 MHz) of compound **2** in $CDCl_3$. 13
Figure S18. NOESY spectrum (600 MHz) of compound **2** in $CDCl_3$....................................13
Figure S19. HR-ESI-MS spectrum of compound **2**. 14
Figure S20. IR spectrum of compound **2**. 14
Figure S21. DP4+ results of compound **2** (Isomer 1: 2*R**, 7*S**, 8*S**, 11*S**, Isomer 2: 2*S**, 7*S**, 8*S**, 11*S**). 15
Figure S22. UV and CD spectrum of compound **2**. 15
Figure S23. ^{1}H NMR spectrum (600 MHz) of compound **3** in $CDCl_3$...................................16
Figure S24. ^{13}C NMR spectrum (600 MHz) of compound **3** in $CDCl_3$. 16
Figure S25. DEPT spectrum (150 MHz) of compound **3** in $CDCl_3$......................................17
Figure S26. ^{1}H-^{1}H COSY spectrum (600 MHz) of compound **3** in $CDCl_3$............................17
Figure S27. HSQC spectrum (600 MHz) of compound **3** in $CDCl_3$. 18
Figure S28. HMBC spectrum (600 MHz) of compound **3** in $CDCl_3$. 18
Figure S29. NOESY spectrum (600 MHz) of compound **3** in $CDCl_3$....................................19
Figure S30. HR-ESI-MS spectrum of compound **3**. 19
Figure S31. IR spectrum of compound **3**. 20
Figure S32. DP4+ results of compound **3** (Isomer 1: 2*R**, 7*R**, 8*S**; Isomer 2: 2*R**, 7*S**, 8*R**). 20
Figure S33. UV and CD spectrum of compound **3**. 21
Figure S34. ^{1}H NMR spectrum (600 MHz) of compound **4** in $CDCl_3$...................................22
Figure S35. ^{13}C NMR spectrum (600 MHz) of compound **4** in $CDCl_3$. 22
Figure S36. DEPT spectrum (150 MHz) of compound **4** in $CDCl_3$......................................23
Figure S37. ^{1}H-^{1}H COSY spectrum (600 MHz) of compound **4** in $CDCl_3$............................23
Figure S38. HSQC spectrum (600 MHz) of compound **4** in $CDCl_3$. 24
Figure S39. HMBC spectrum (600 MHz) of compound **4** in $CDCl_3$. 24

Figure S40. NOESY spectrum (600 MHz) of compound **4** in $CDCl_3$....................................25
Figure S41. HR-ESI-MS spectrum of compound **4**. 25
Figure S42. IR spectrum of compound **4**. 26
Figure S43. UV and CD spectrum of compound **4**. 27
Figure S44. DP4+ results of compound **4** (Isomer 1: 1*S**, 2*R**, 7*S**. 8*S**; Isomer 2: 1*S**, 2*S**, 7*S**. 8*S**; Isomer 3: 1*R**, 2*R**, 7*S**. 8*S**; Isomer 4: 1*R**, 2*S**, 7*S**. 8*S**)........................27
Figure S45. ^{1}H NMR spectrum (600 MHz) of compound **5** in $CDCl_3$..................................28
Figure S46. ^{13}C NMR spectrum (600 MHz) of compound **5** in $CDCl_3$. 28
Figure S47. DEPT spectrum (150 MHz) of compound **5** in $CDCl_3$.......................................29
Figure S48. ^{1}H-^{1}H COSY spectrum (600 MHz) of compound **5** in $CDCl_3$............................29
Figure S49. HSQC spectrum (600 MHz) of compound **5** in $CDCl_3$. 30
Figure S50. HMBC spectrum (600 MHz) of compound **5** in $CDCl_3$.....................................30
Figure S51. NOESY spectrum (600 MHz) of compound **5** in $CDCl_3$....................................31
Figure S52. HR-ESI-MS spectrum of compound **5**. 31
Figure S53. IR spectrum of compound **5**. 32
Figure S54. UV and CD spectrum of compound **5**. 33
Figure S55. DP4+ results of compound **5** (Isomer 1: 2*R**, 7*S**, 8*S**; Isomer 2: 2*S**, 7*S**, 8*S**). 33
Figure S56. ^{1}H NMR spectrum (600 MHz) of compound **6** in $CDCl_3$..................................34
Figure S57. ^{13}C NMR spectrum (600 MHz) of compound **6** in $CDCl_3$. 34
Figure S58. DEPT spectrum (150 MHz) of compound **6** in $CDCl_3$.......................................35
Figure S59. ^{1}H-^{1}H COSY spectrum (600 MHz) of compound **6** in $CDCl_3$............................35
Figure S60. HSQC spectrum (600 MHz) of compound **6** in $CDCl_3$. 36
Figure S61. HMBC spectrum (600 MHz) of compound **6** in $CDCl_3$.....................................36
Figure S62. NOESY spectrum (600 MHz) of compound **6** in $CDCl_3$....................................37
Figure S63. HR-ESI-MS spectrum of compound **6**. 37
Figure S64. IR spectrum of compound **6**. 38
Figure S65. UV and CD spectrum of compound **6**. 39
Figure S66. DP4+ results of compound **6** (Isomer 1: 1*S**, 2*R**, 7*S**, 8*S**, 15*S**; Isomer 2: 1*S**, 2*S**, 7*S**, 8*S**, 15*S**; Isomer 3: 1*R**, 2*R**, 7*S**, 8*S**, 15*R**; Isomer 4: 1*R**, 2*S**, 7*S**, 8*S**, 15*R**)..39
Figure S67. ^{1}H NMR spectrum (600 MHz) of compound **7** in $CDCl_3$..................................40
Figure S68. ^{13}C NMR spectrum (600 MHz) of compound **7** in $CDCl_3$. 40
Figure S69. DEPT spectrum (150 MHz) of compound **7** in $CDCl_3$.......................................41
Figure S70. ^{1}H-^{1}H COSY spectrum (600 MHz) of compound **7** in $CDCl_3$............................41
Figure S71. HSQC spectrum (600 MHz) of compound **7** in $CDCl_3$. 42
Figure S72. HMBC spectrum (600 MHz) of compound **7** in $CDCl_3$.....................................42
Figure S73. NOESY spectrum (600 MHz) of compound **7** in $CDCl_3$....................................43
Figure S74. HR-ESI-MS spectrum of compound **7**. 43
Figure S75. IR spectrum of compound **7**. 44
Figure S76. UV and CD spectrum of compound **7**. 45
Figure S77. DP4+ results of compound **7** (Isomer 1: 1*S**, 2*R**, 7*S**, 8*S**, 15*S**; Isomer 2: 1*S**, 2*S**, 7*S**, 8*S**, 15*S**; Isomer 3: 1*R**, 2*R**, 7*S**, 8*S**, 15*R**; Isomer 4: 1*R**, 2*S**, 7*S**, 8*S**, 15*R**)..45

Figure S78. ^{1}H NMR spectrum (600 MHz) of compound **8** in $CDCl_3$....................................46
Figure S79. ^{13}C NMR spectrum (600 MHz) of compound **8** in $CDCl_3$. 46
Figure S80. DEPT spectrum (150 MHz) of compound **8** in $CDCl_3$.......................................47
Figure S81. ^{1}H-^{1}H COSY spectrum (600 MHz) of compound **8** in $CDCl_3$.............................47
Figure S82. HSQC spectrum (600 MHz) of compound **8** in CDCl3.......................................48
Figure S83. HMBC spectrum (600 MHz) of compound **8** in CDCl3. 48
Figure S84. NOESY spectrum (600 MHz) of compound **8** in $CDCl_3$...................................49
Figure S85. HR-ESI-MS spectrum of compound **8**. 49
Figure S86. IR spectrum of compound **8**. 50
Figure S87. UV and CD spectrum of compound **8**. 51
Figure S88. DP4+ results of compound **8** (Isomer 1: 2*R**, 7*R**, 8*S**, 12*S**; Isomer 2: 2*R**, 7*R**, 8*S**, 12*R**; Isomer 3: 2*S**, 7*R**, 8*S**, 12*R**; Isomer 4: 2*S**, 7*R**, 8*S**, 12*S**). 51

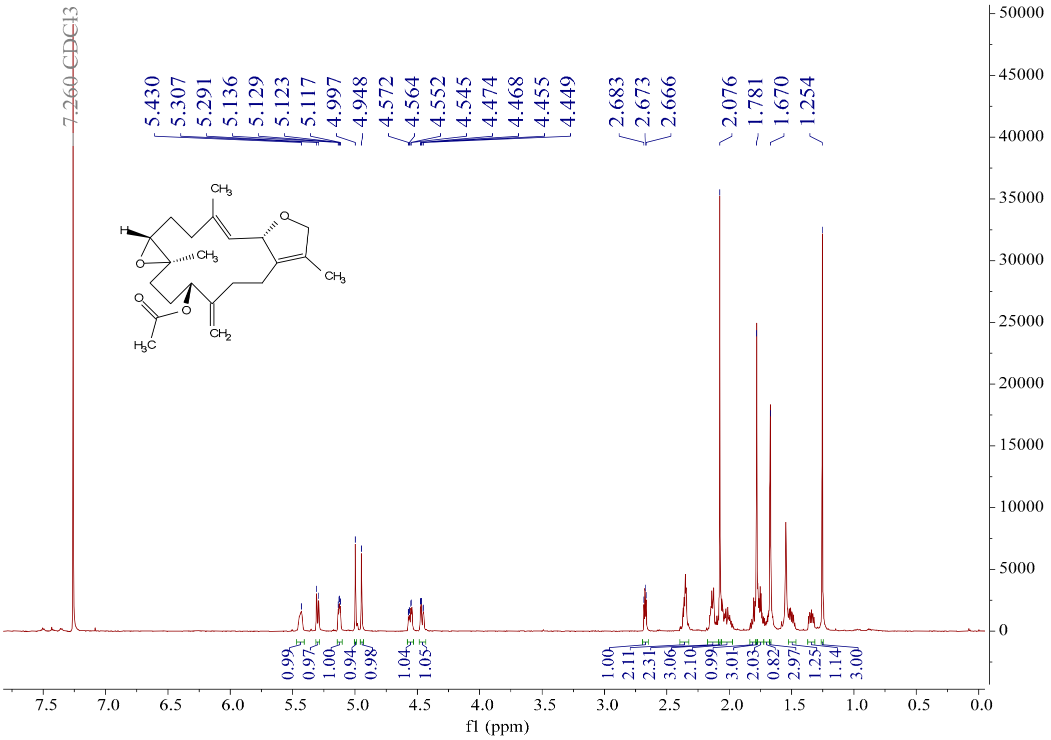

Figure S1. ^{1}**H NMR spectrum (600 MHz) of compound 1 in** $CDCl_3$**.**

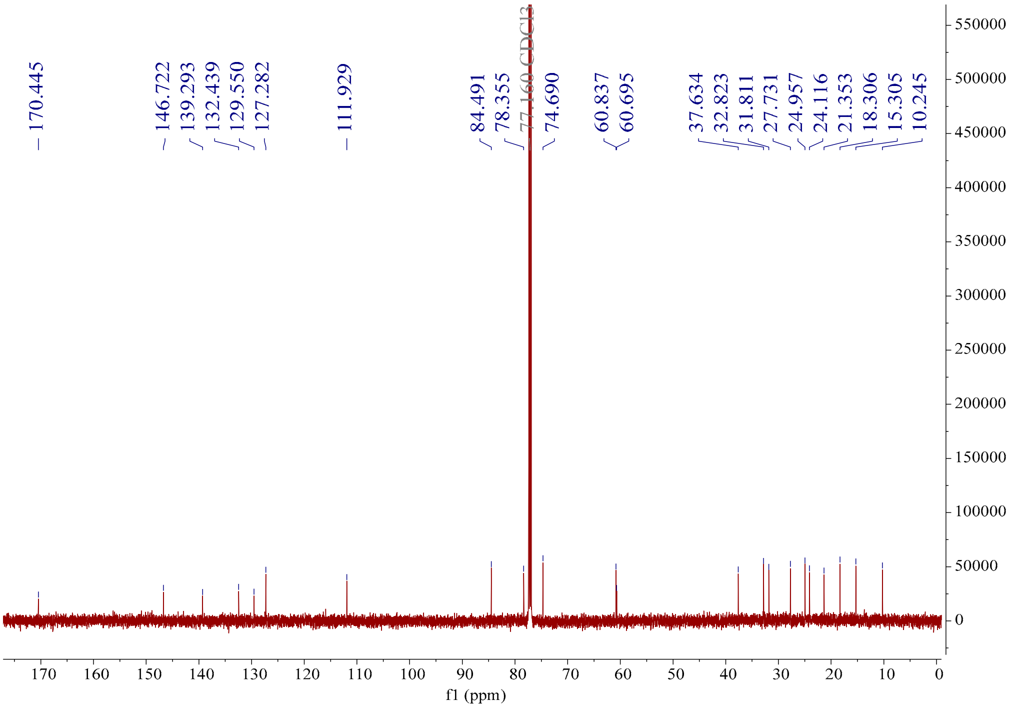

Figure S2. ^{13}**C NMR spectrum (600 MHz) of compound 1 in** $CDCl_3$**.**

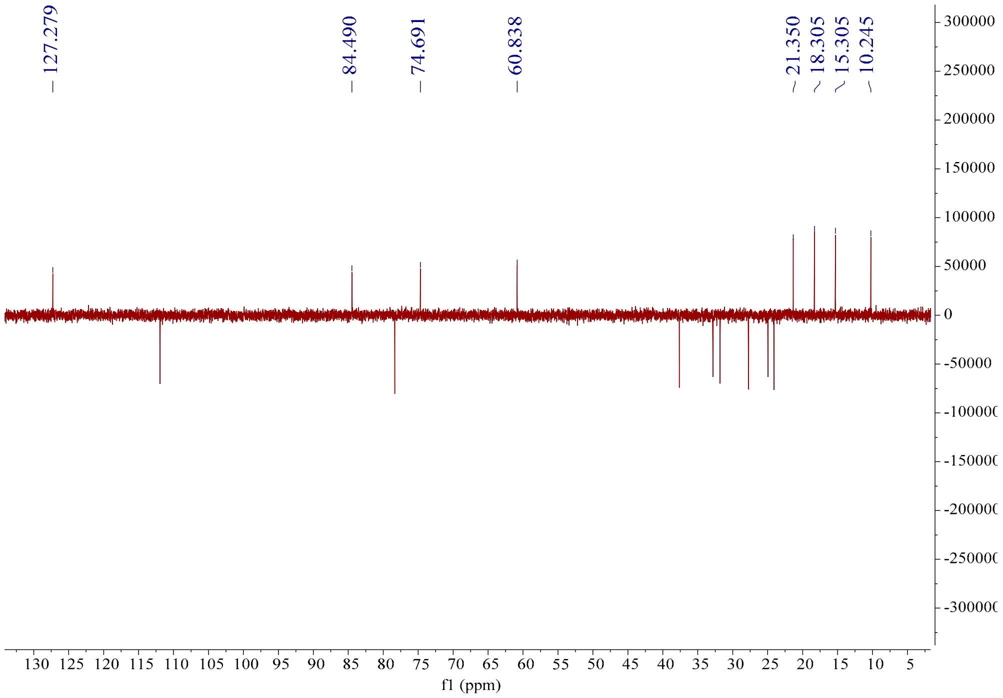

Figure S3. DEPT spectrum (150 MHz) of compound 1 in $CDCl_3$.

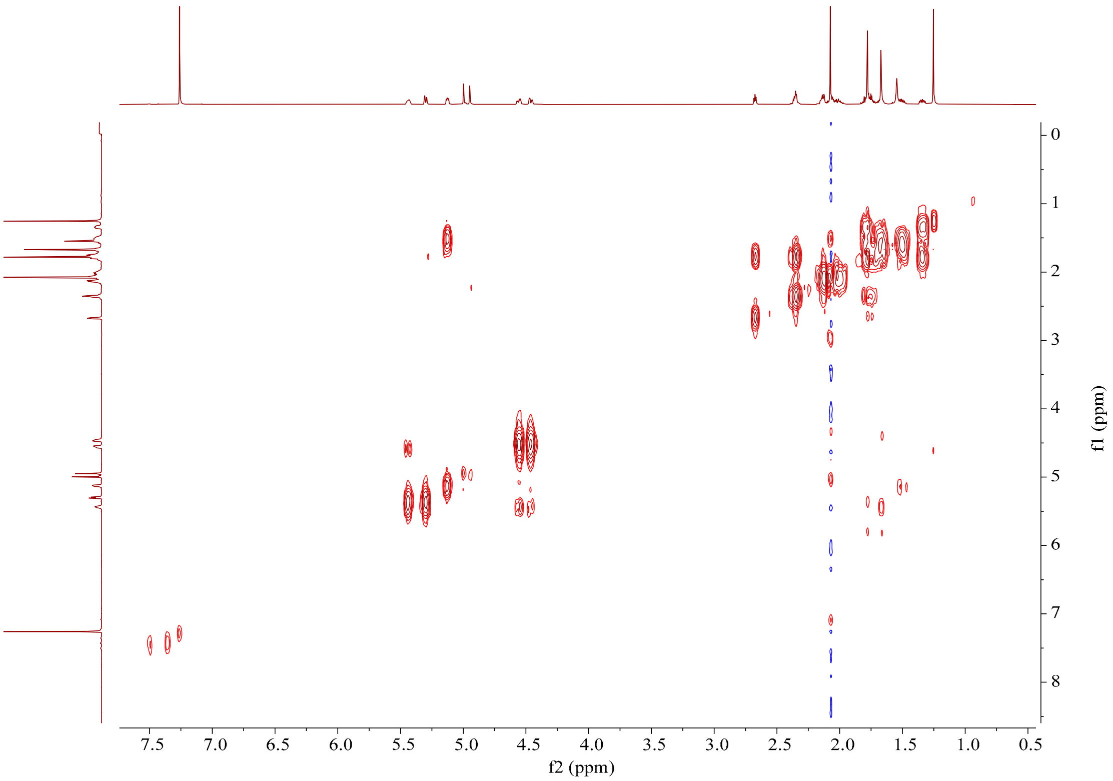

Figure S4. ^1H-^1H COSY spectrum (600 MHz) of compound 1 in $CDCl_3$.

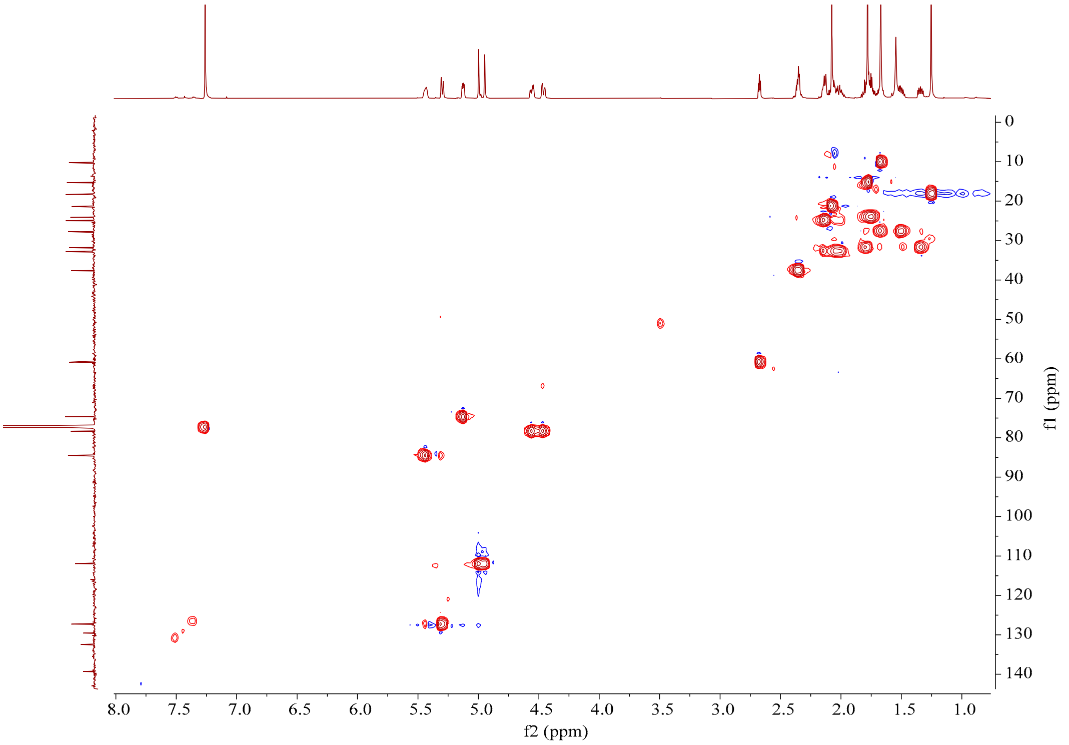

Figure S5. HSQC spectrum (600 MHz) of compound 1 in $CDCl_3$.

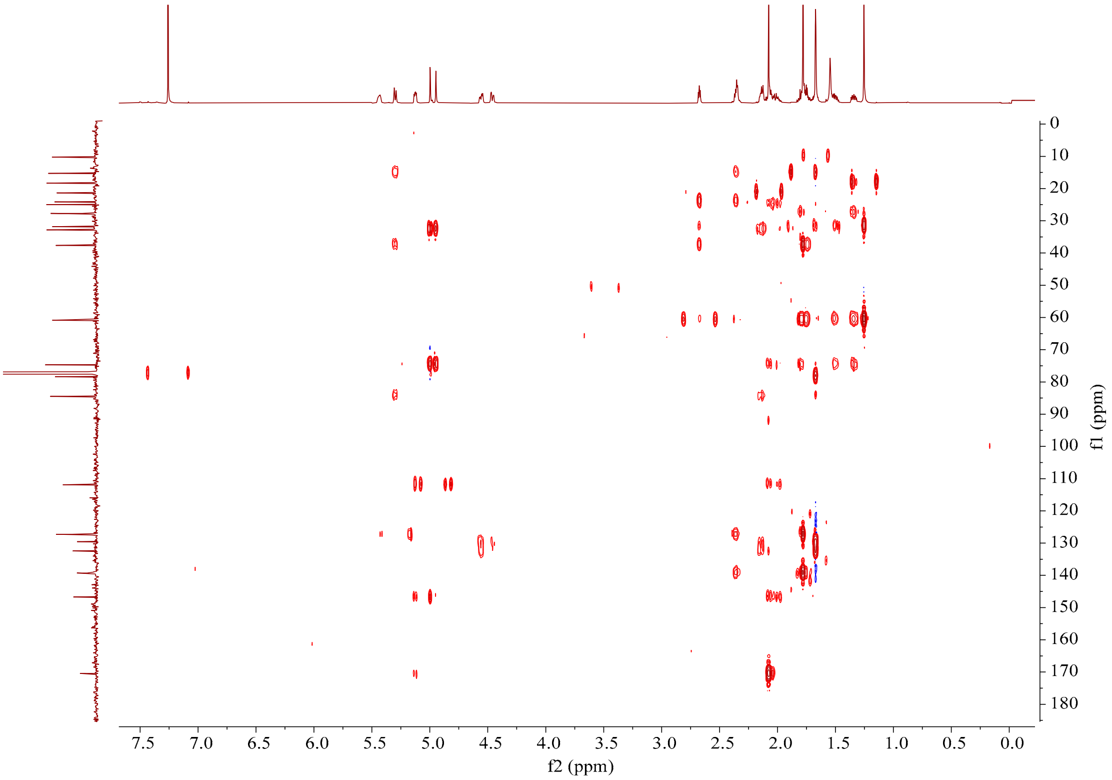

Figure S6. HMBC spectrum (600 MHz) of compound 1 in $CDCl_3$.

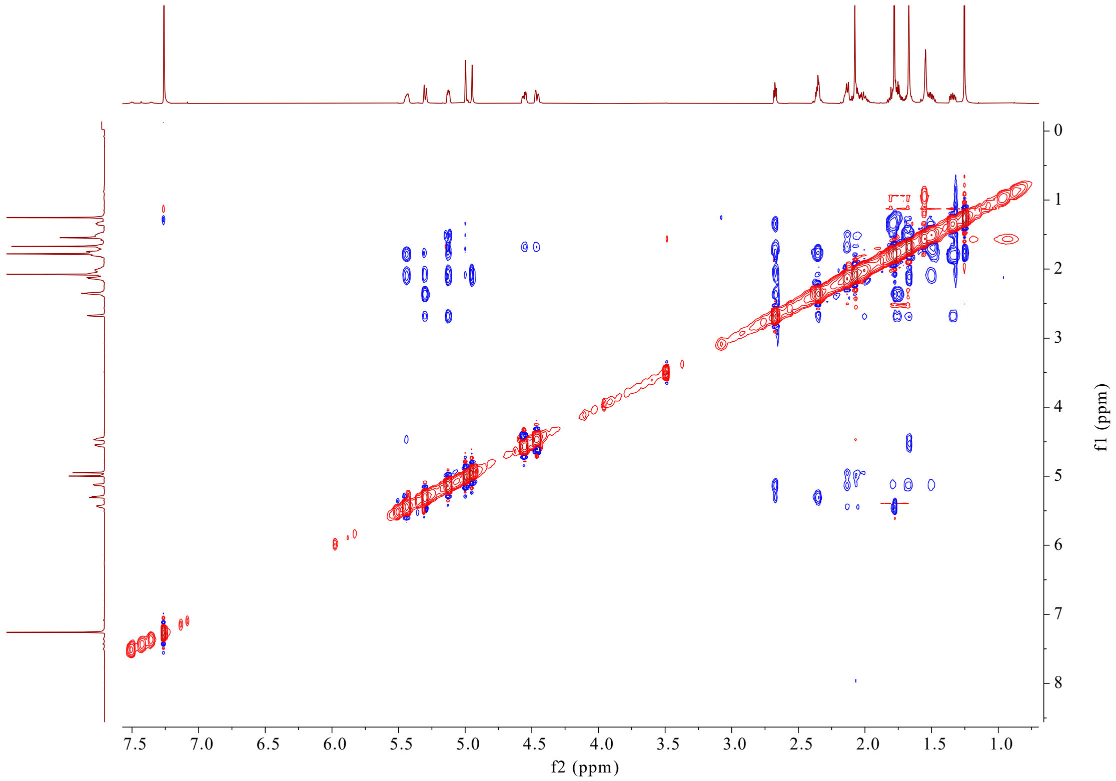

Figure S7. NOESY spectrum (600 MHz) of compound 1 in $CDCl_3$.

MS spectra

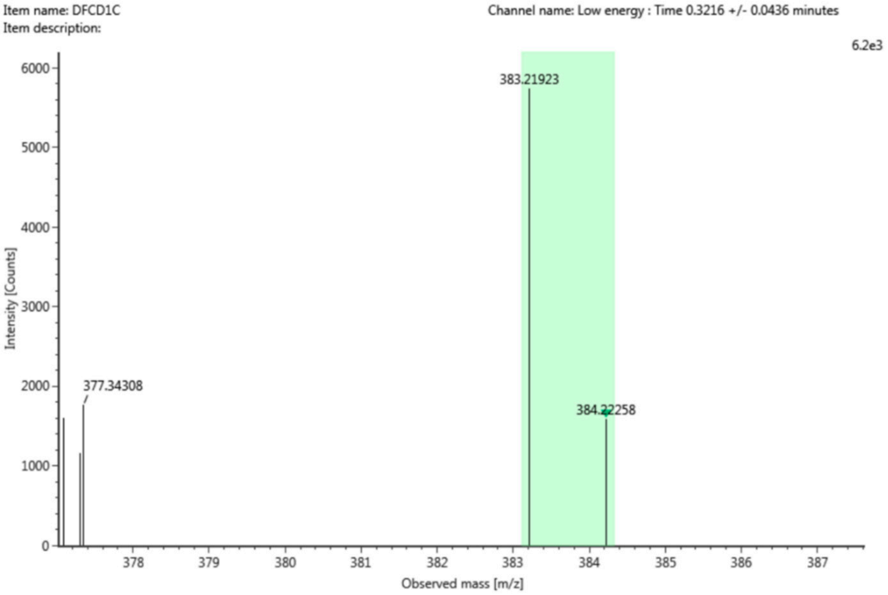

Formula Calculator Results

Formula	Neutral mass (Da)	Observed m/z	Observed RT (min)	Mass error (mDa)	Mass error (ppm)	Adducts
C22H32O4	360.23006	383.2192	0.32	0.0	-0.1	+Na

Figure S8. HR-ESI-MS spectrum of compound 1.

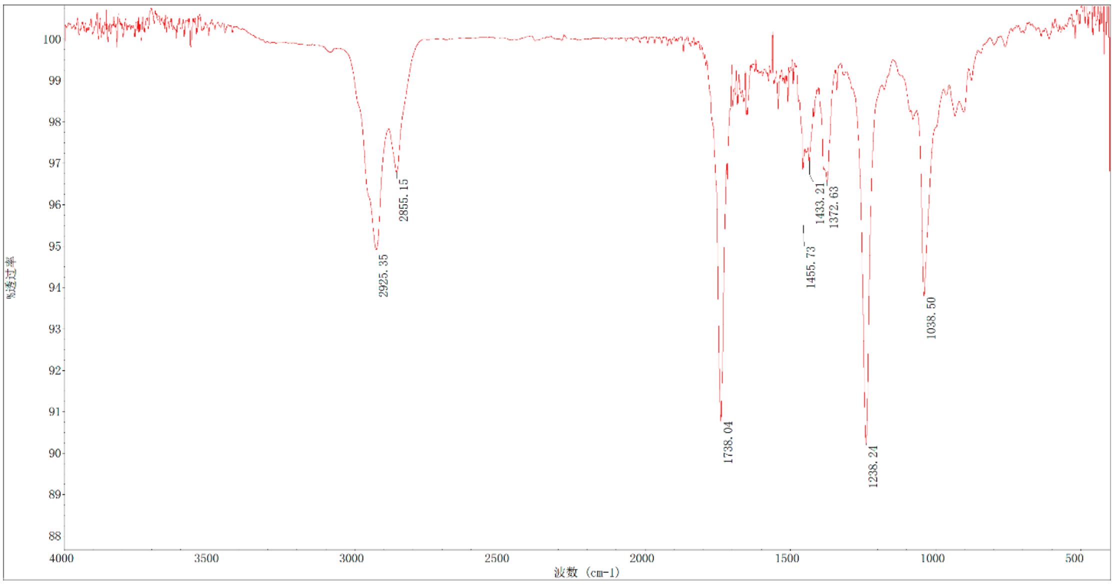

Figure S9. IR spectrum of compound 1.

Functional		Solvent?		Basis Set		Type of Data	
mPW1PW91		PCM		6-311+G(d,p)		Shielding Tensors	
	Isomer 1	Isomer 2	Isomer 3	Isomer 4	Isomer 5	Isomer 6	
sDP4+ (H data)	100.00%	0.00%	–	–	–	–	
sDP4+ (C data)	79.01%	20.99%	–	–	–	–	
sDP4+ (all data)	100.00%	0.00%	–	–	–	–	
uDP4+ (H data)	99.97%	0.03%	–	–	–	–	
uDP4+ (C data)	99.78%	0.22%	–	–	–	–	
uDP4+ (all data)	100.00%	0.00%	–	–	–	–	
DP4+ (H data)	100.00%	0.00%	–	–	–	–	
DP4+ (C data)	99.94%	0.06%	–	–	–	–	
DP4+ (all data)	100.00%	0.00%	–	–	–	–	

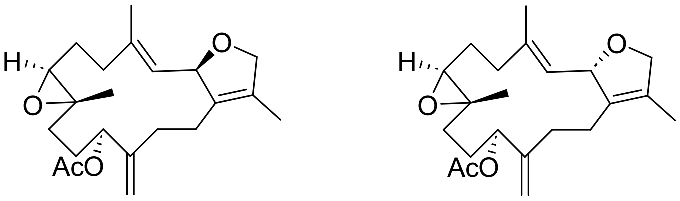

Isomer 1 Isomer 2

Figure S10. DP4+ results of compound 1 (Isomer 1: 2*R, 7*S**, 8*S**, 11*S**; Isomer 2: 2*S**, 7*S**, 8*S**, 11*S**).**

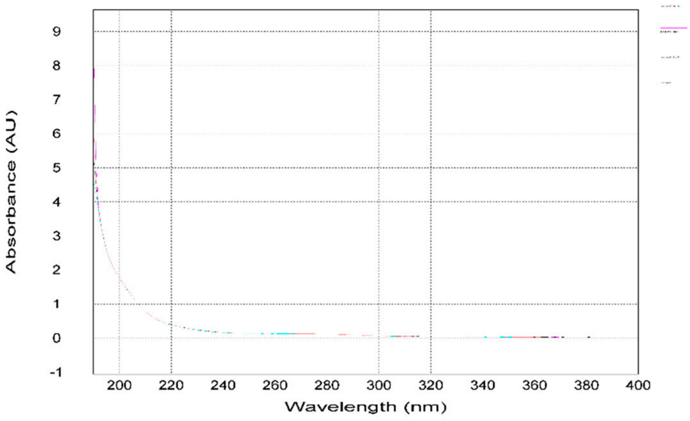

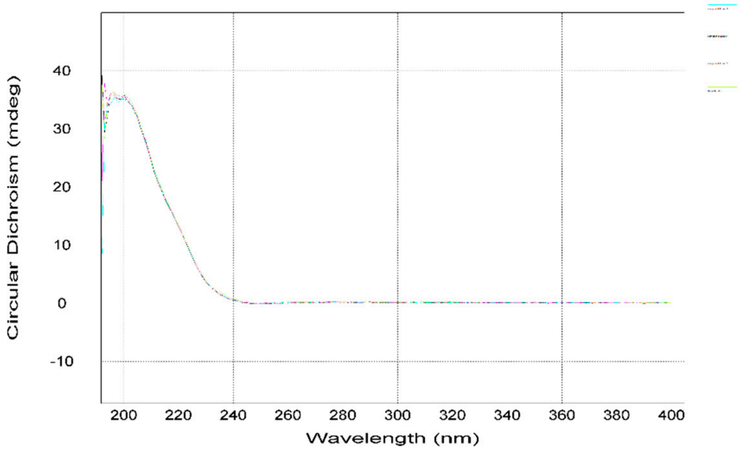

Figure S11. UV and CD spectrum of compound 1.

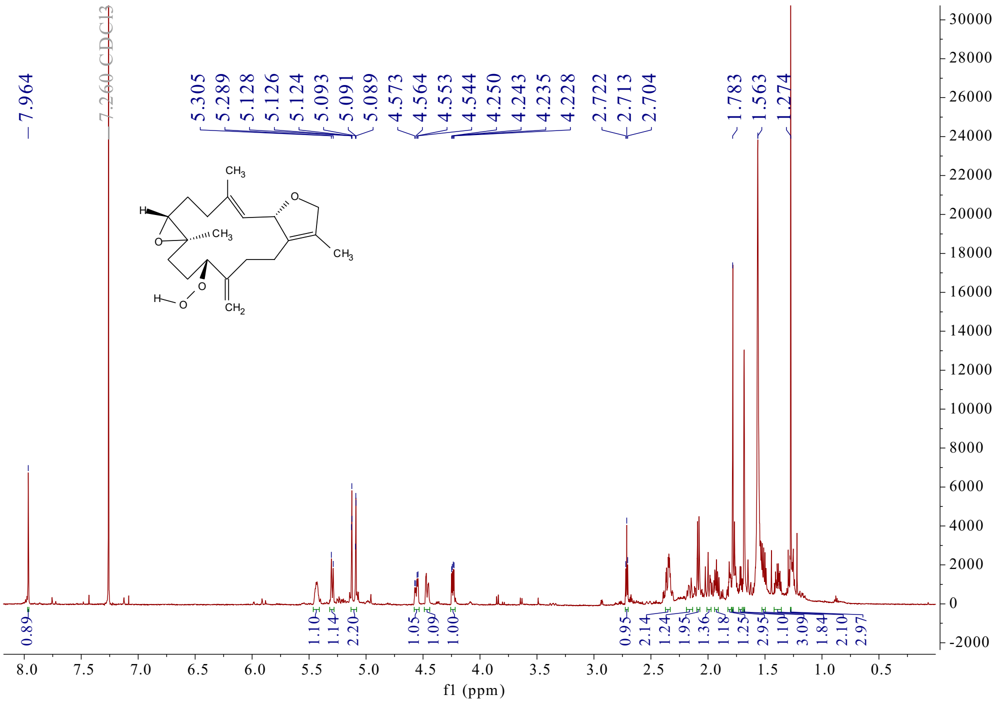

Figure S12. ^{1}H NMR spectrum (600 MHz) of compound 2 in $CDCl_3$.

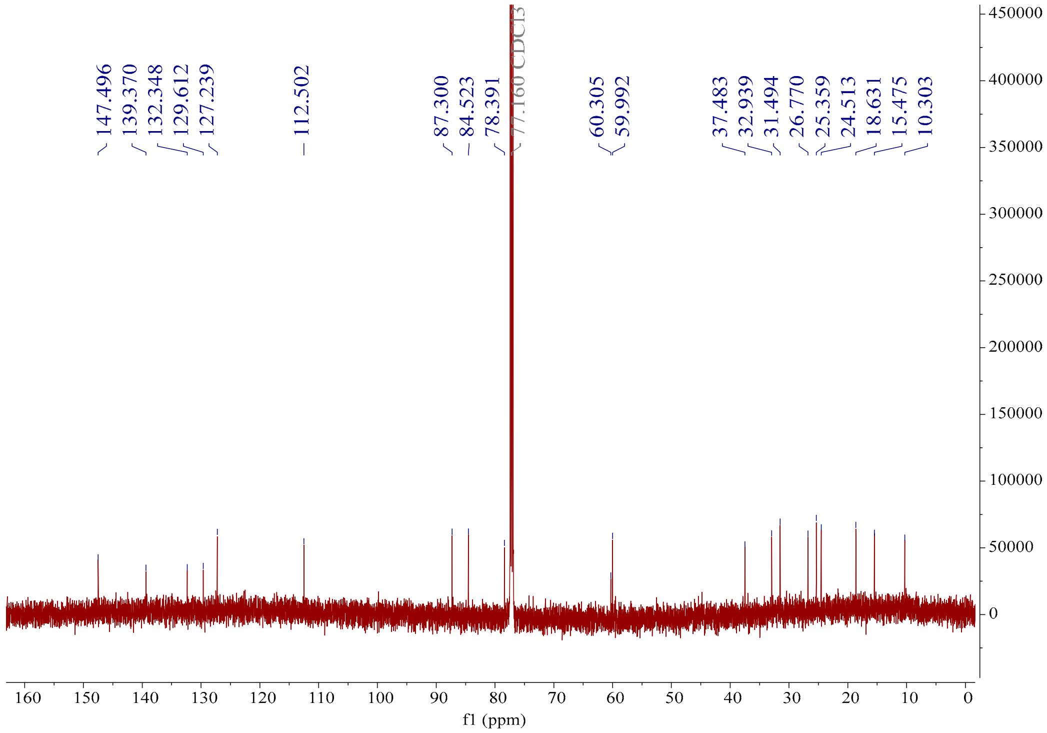

Figure S13. ^{13}C NMR spectrum (600 MHz) of compound 2 in $CDCl_3$.

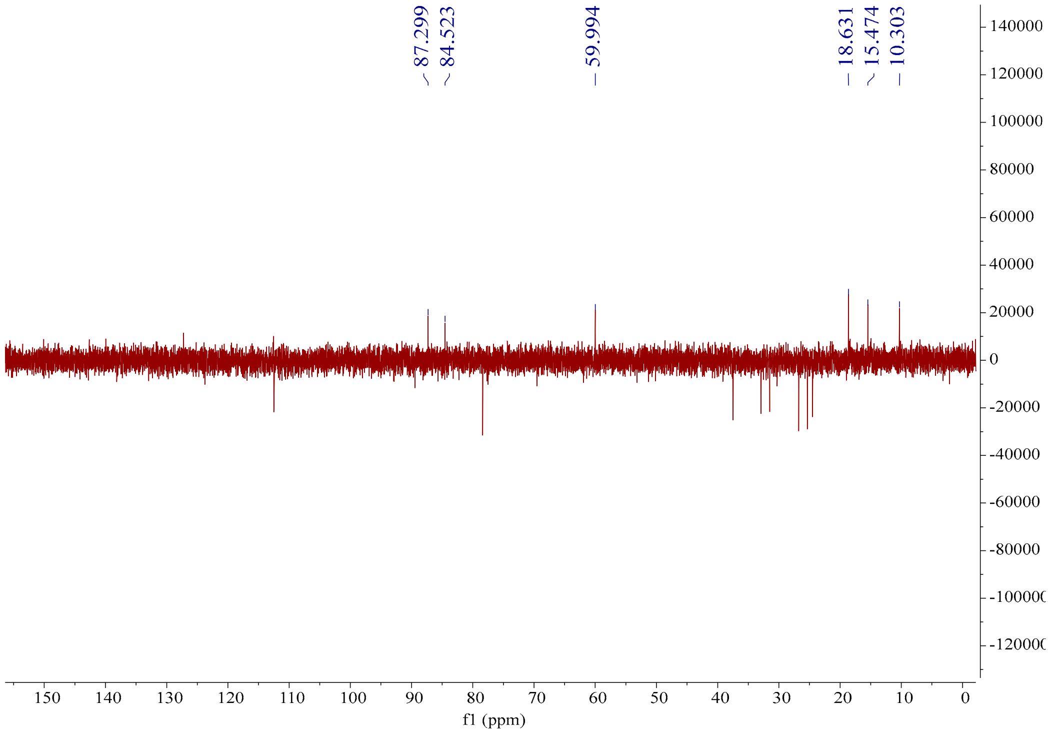

Figure S14. DEPT spectrum (150 MHz) of compound 2 in $CDCl_3$.

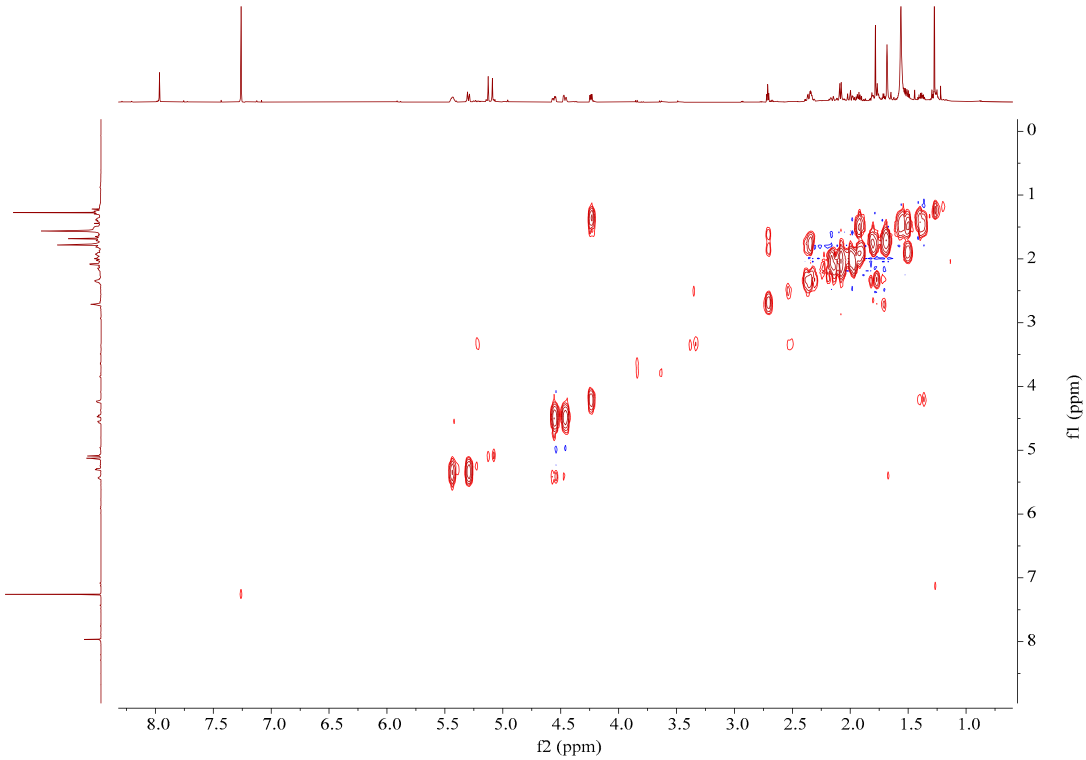

Figure S15. ^{1}H-^{1}H COSY spectrum (600 MHz) of compound 2 in $CDCl_3$.

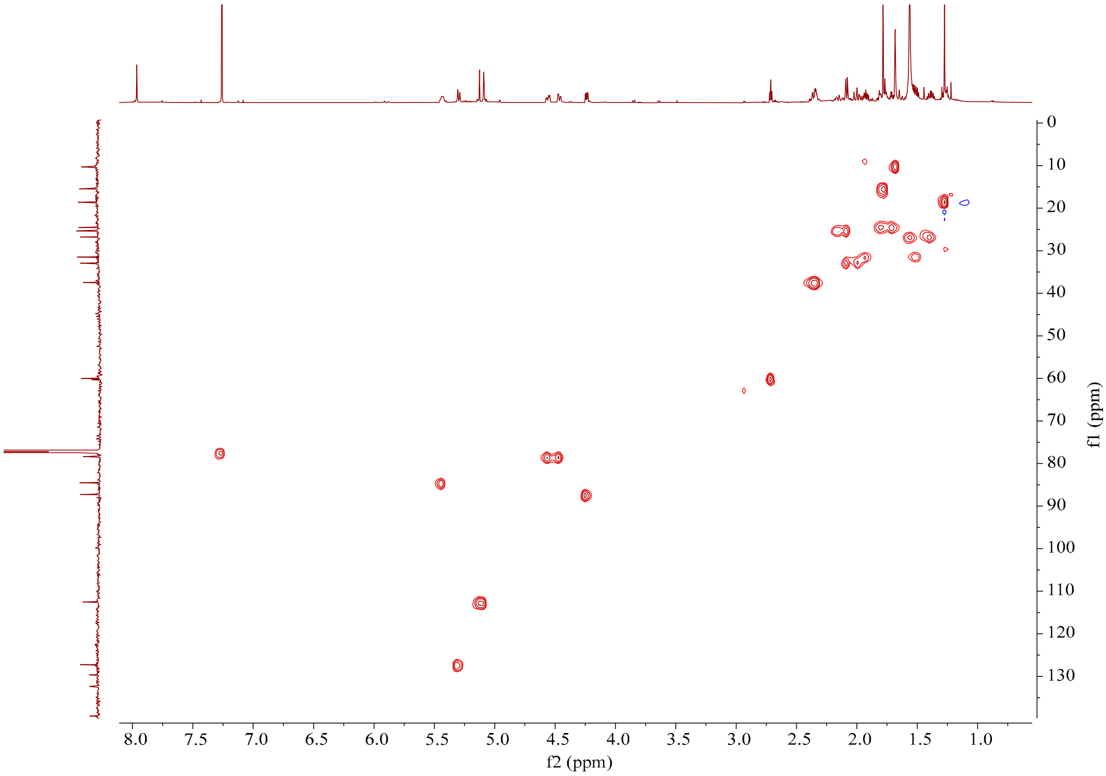

Figure S16. HSQC spectrum (600 MHz) of compound 2 in $CDCl_3$.

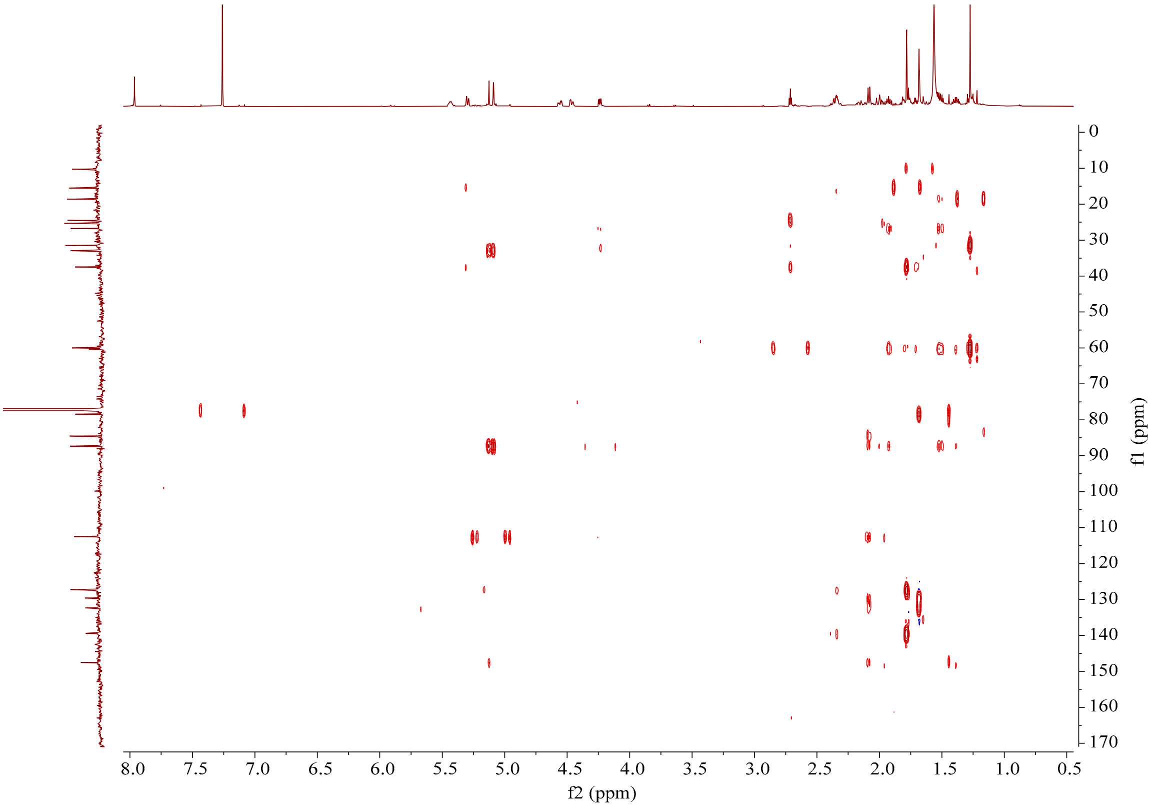

Figure S17. HMBC spectrum (600 MHz) of compound 2 in $CDCl_3$.

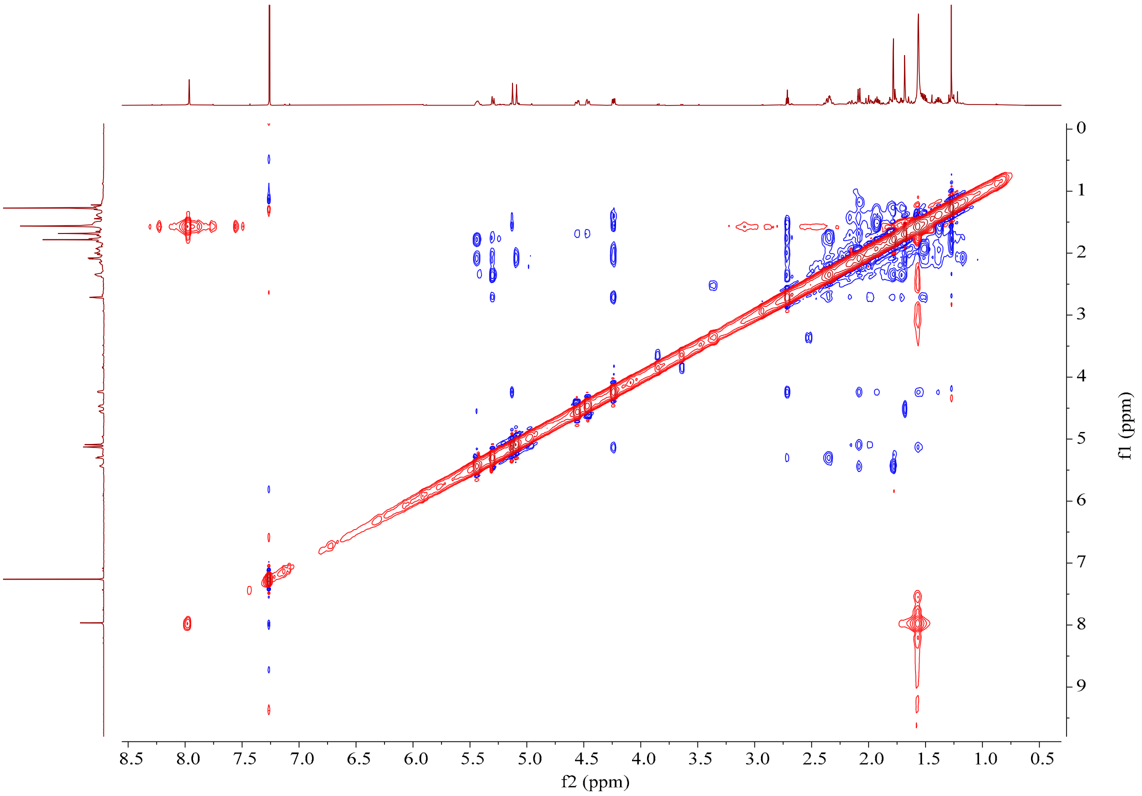

Figure S18. NOESY spectrum (600 MHz) of compound 2 in $CDCl_3$.

MS spectra

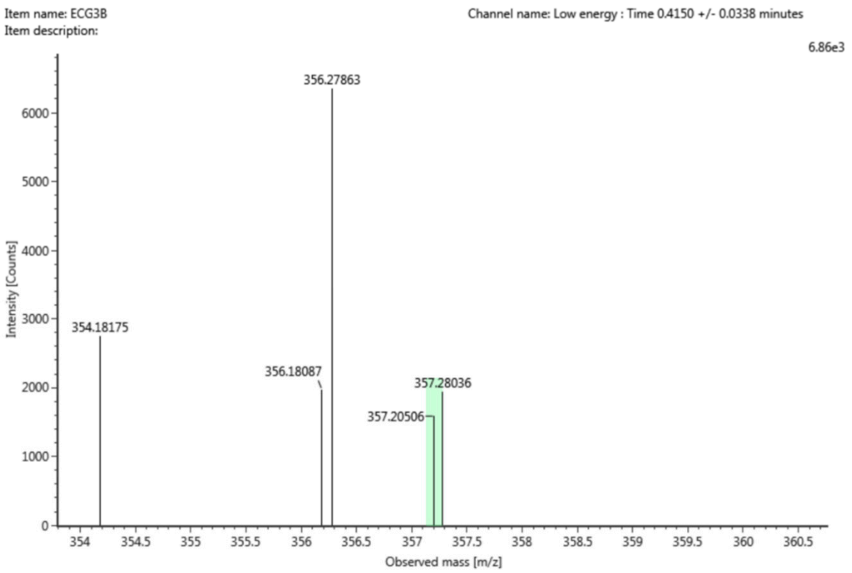

Formula Calculator Results

Formula	Neutral mass (Da)	Observed m/z	Observed RT (min)	Mass error (mDa)	Mass error (ppm)	Adducts
C20H30O4	334.21441	357.2051	0.42	1.4	4.0	+Na

Figure S19. HR-ESI-MS spectrum of compound 2.

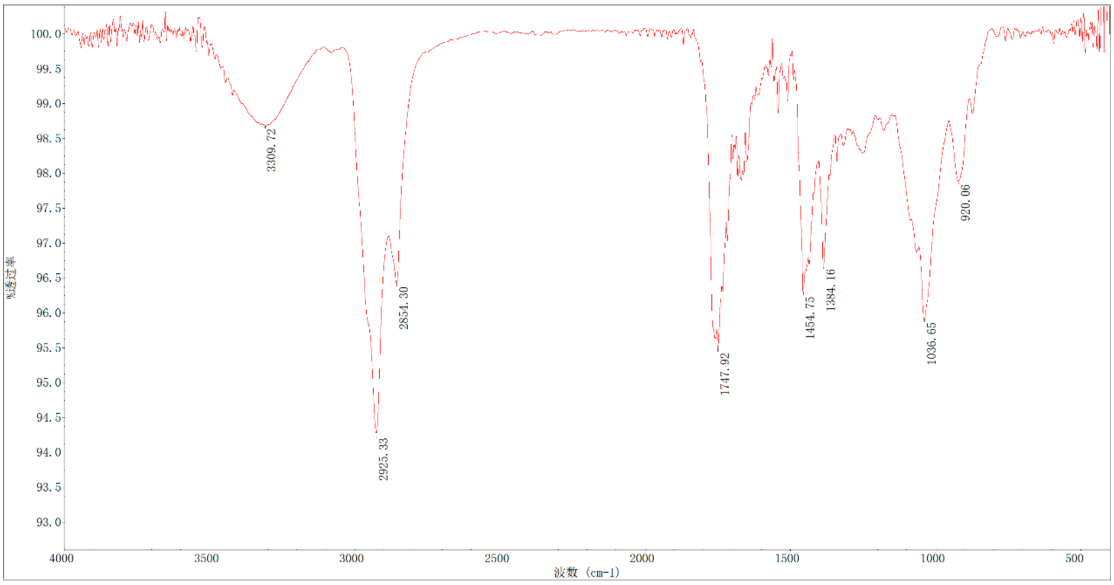

Figure S20. IR spectrum of compound 2.

Functional	Solvent?	Basis Set	Type of Data			
mPW1PW91	PCM	6-311+G(d,p)	Shielding Tensors			
	Isomer 1	Isomer 2	Isomer 3	Isomer 4	Isomer 5	Isomer 6
sDP4+ (H data)	99.72%	0.28%	–	–	–	–
sDP4+ (C data)	99.57%	0.43%	–	–	–	–
sDP4+ (all data)	100.00%	0.00%	–	–	–	–
uDP4+ (H data)	99.91%	0.09%	–	–	–	–
uDP4+ (C data)	99.99%	0.01%	–	–	–	–
uDP4+ (all data)	100.00%	0.00%	–	–	–	–
DP4+ (H data)	100.00%	0.00%	–	–	–	–
DP4+ (C data)	100.00%	0.00%	–	–	–	–
DP4+ (all data)	100.00%	0.00%	–	–	–	–

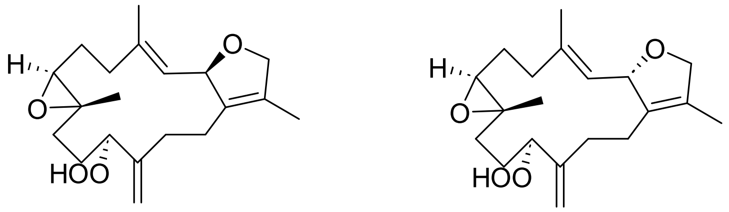

Isomer 1 Isomer 2

Figure S21. DP4+ results of compound 2 (Isomer 1: 2*R, 7*S**, 8*S**, 11*S**, Isomer 2: 2*S**, 7*S**, 8*S**, 11*S**).**

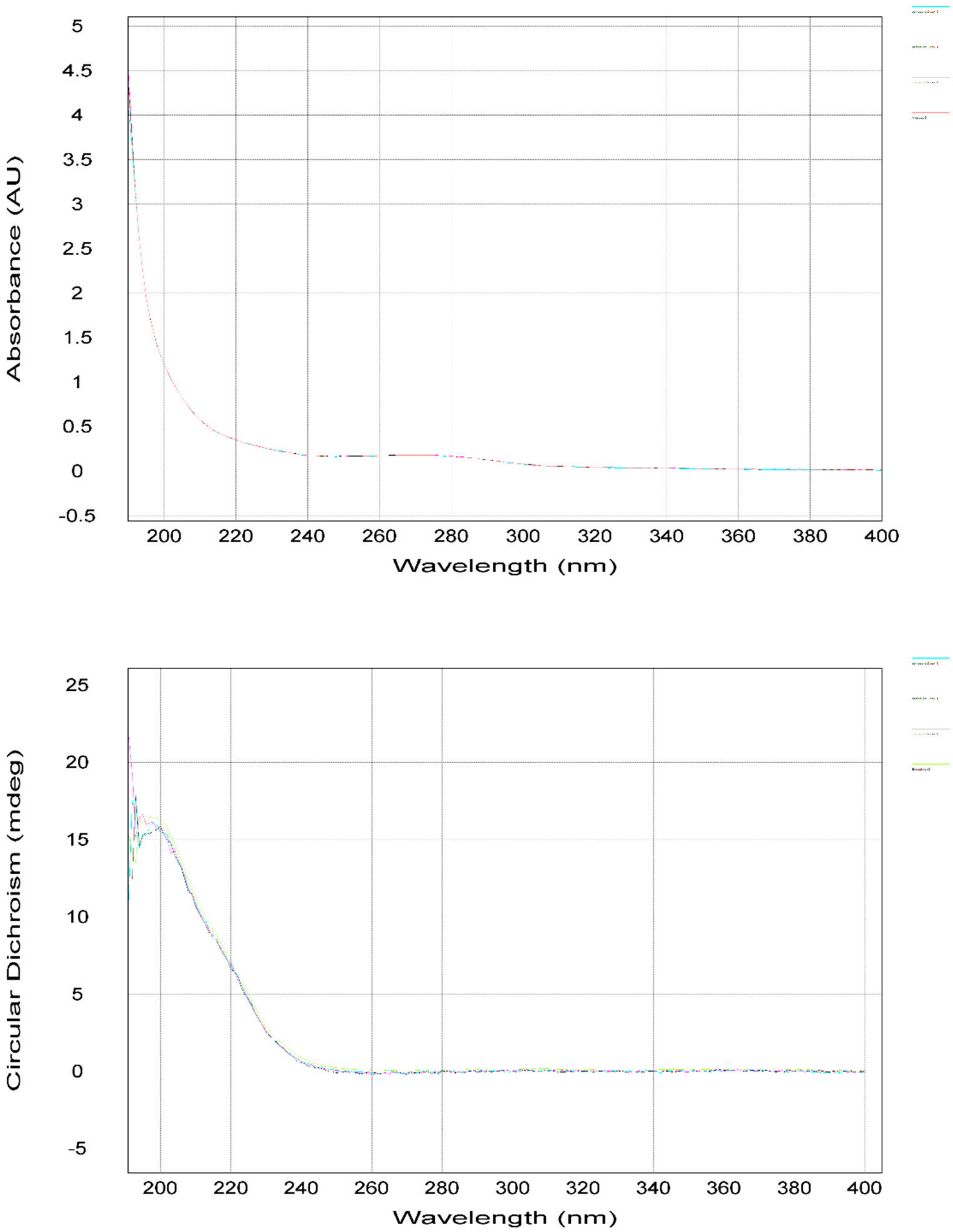

Figure S22. UV and CD spectrum of compound 2.

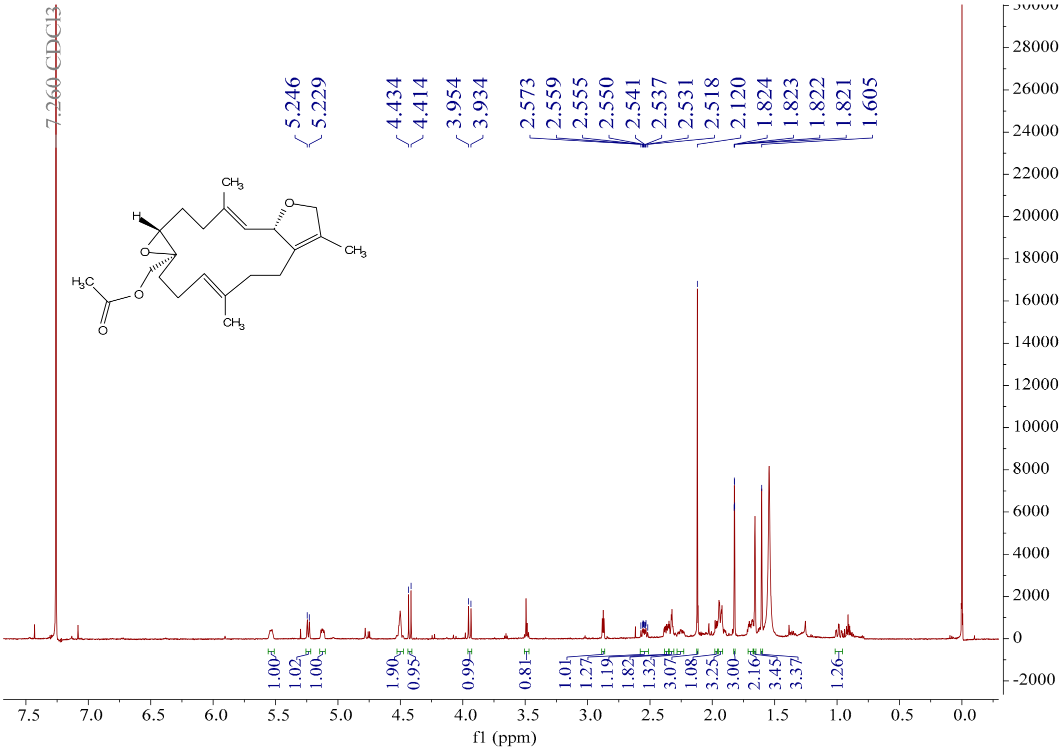

Figure S23. **^{1}H NMR spectrum (600 MHz) of compound 3 in $CDCl_3$.**

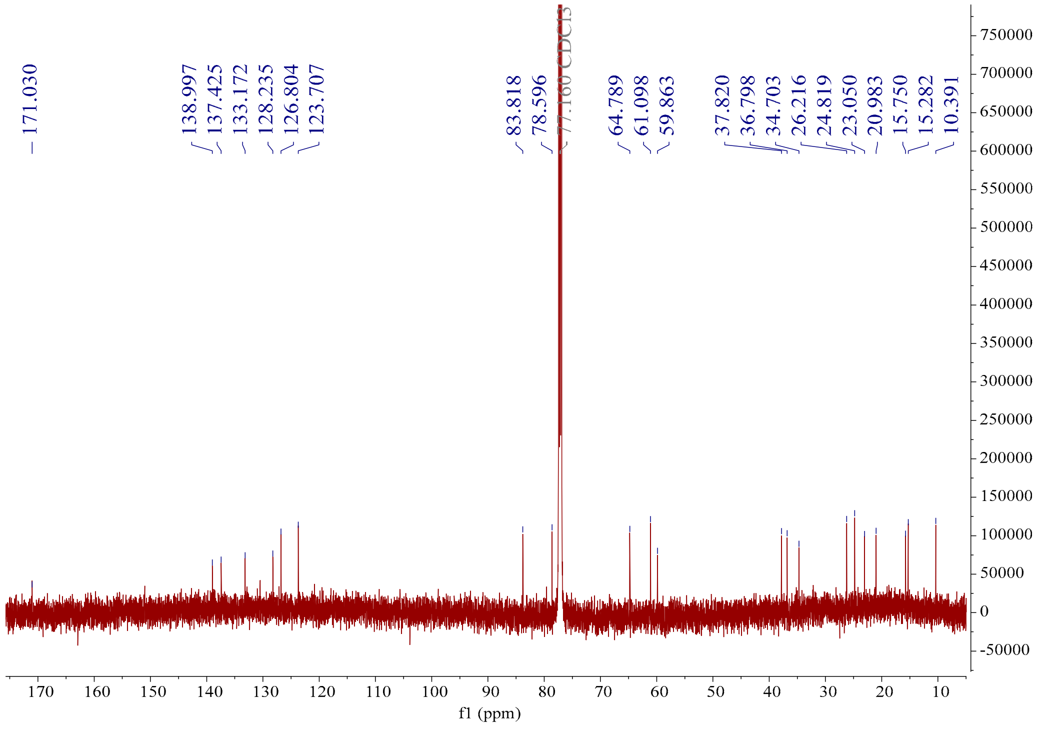

Figure S24. **^{13}C NMR spectrum (600 MHz) of compound 3 in $CDCl_3$.**

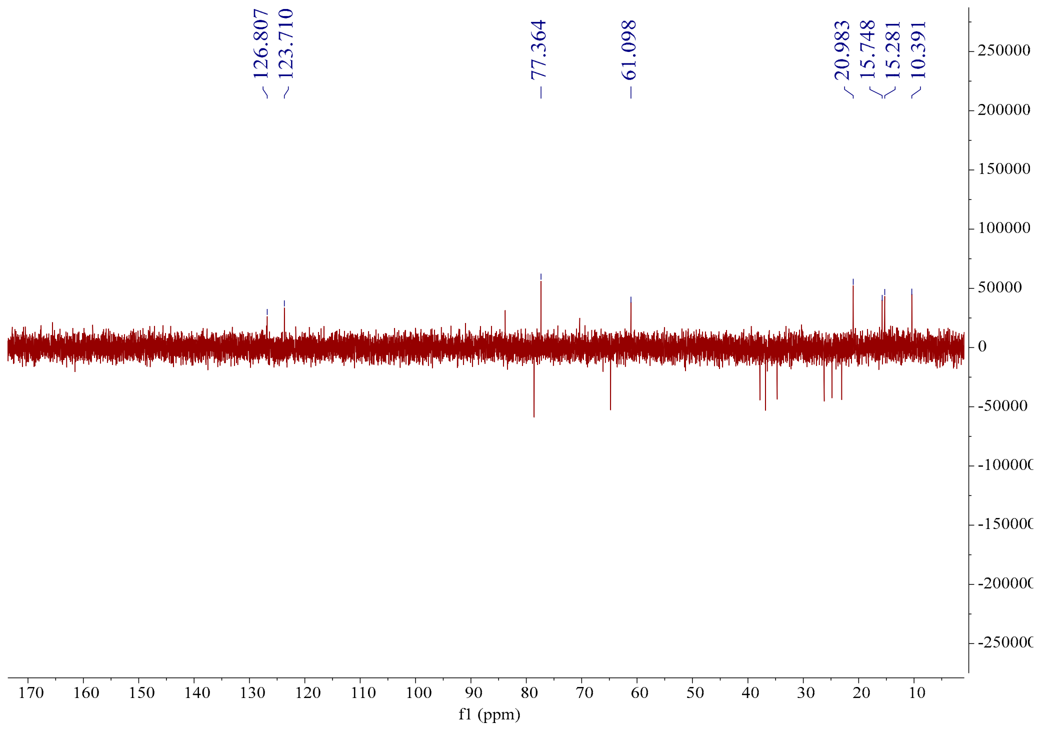

Figure S25. DEPT spectrum (150 MHz) of compound 3 in $CDCl_3$.

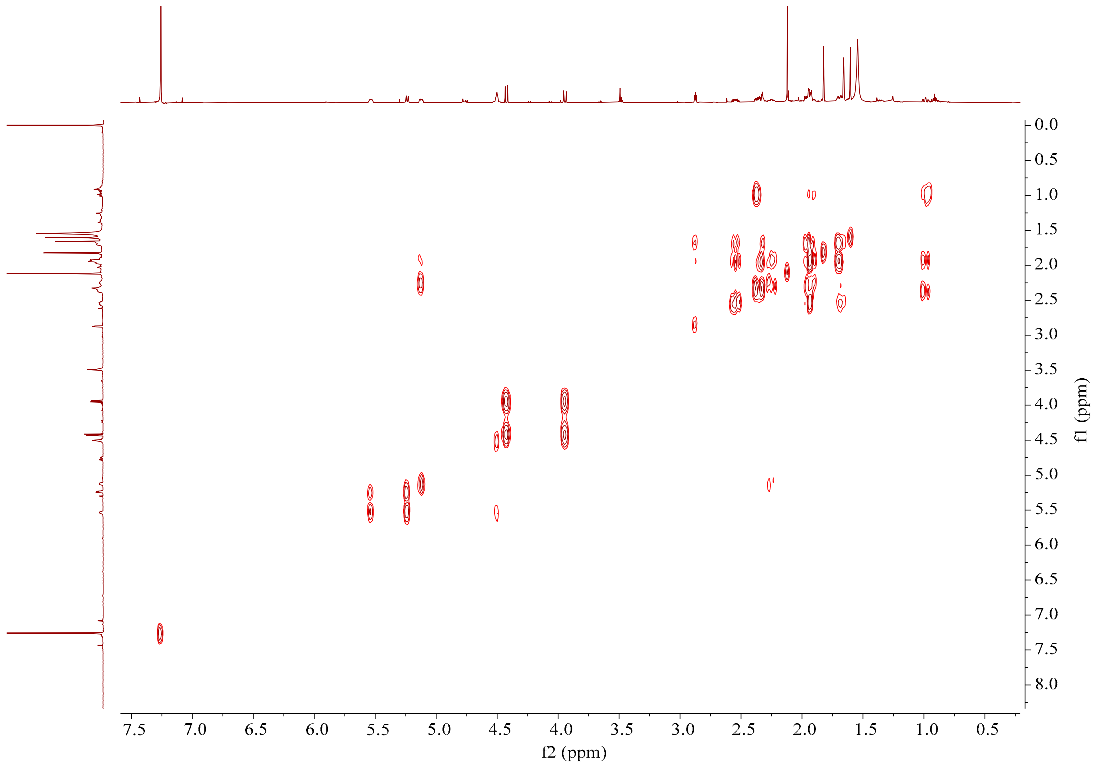

Figure S26. ^1H-^1H COSY spectrum (600 MHz) of compound 3 in $CDCl_3$.

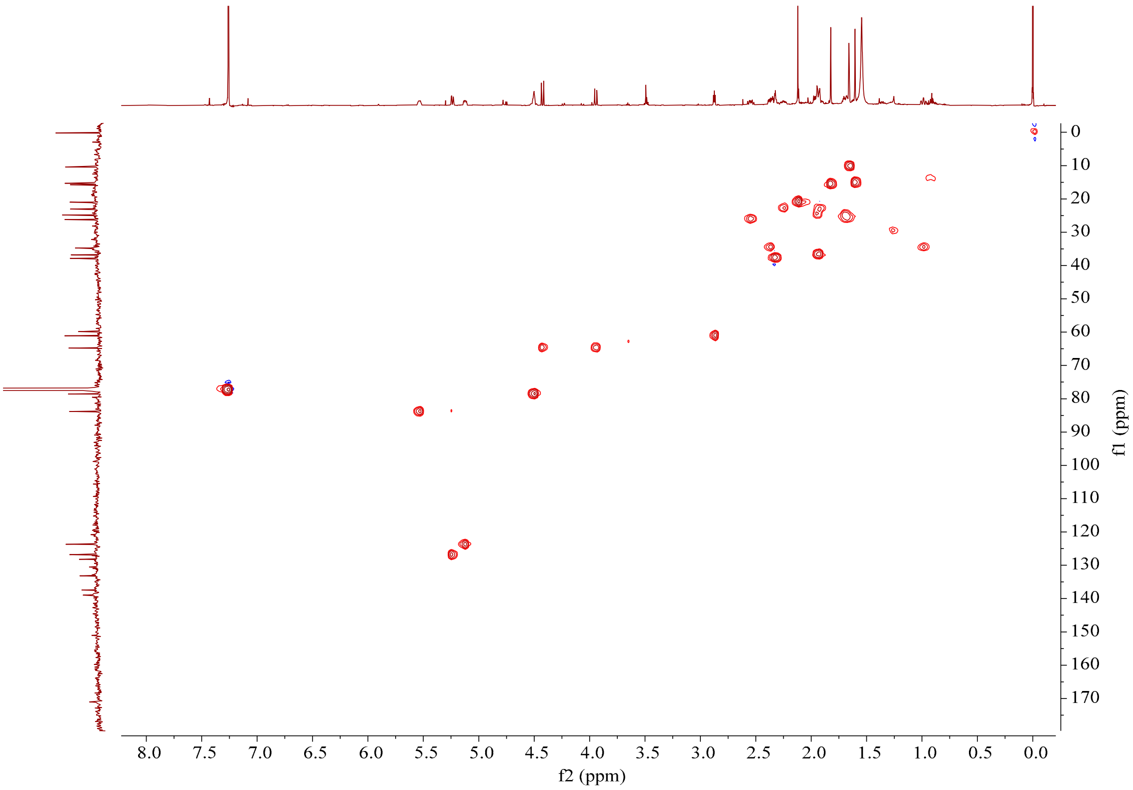

Figure S27. HSQC spectrum (600 MHz) of compound 3 in $CDCl_3$.

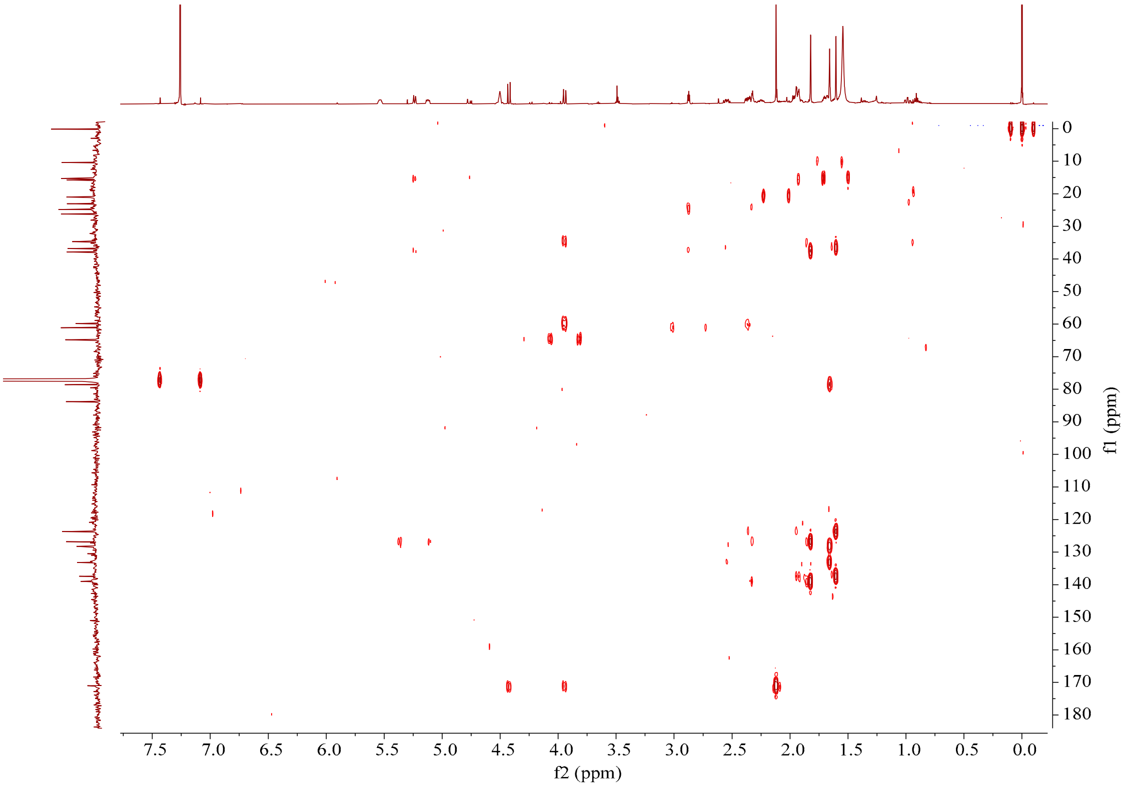

Figure S28. HMBC spectrum (600 MHz) of compound 3 in $CDCl_3$.

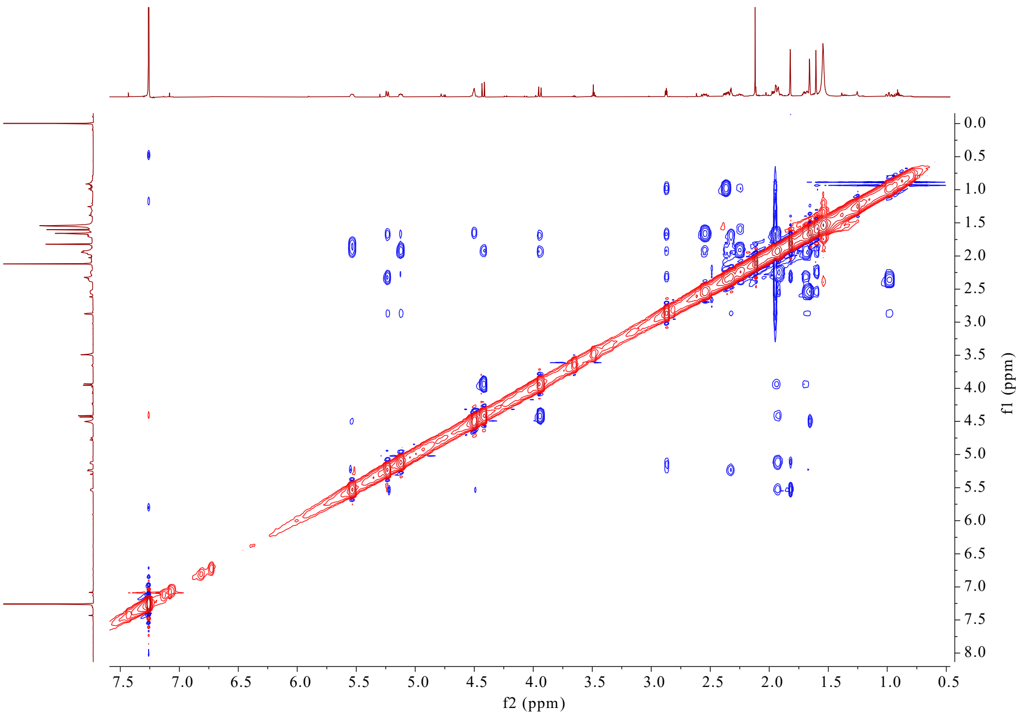

Figure S29. NOESY spectrum (600 MHz) of compound 3 in $CDCl_3$.

MS spectra

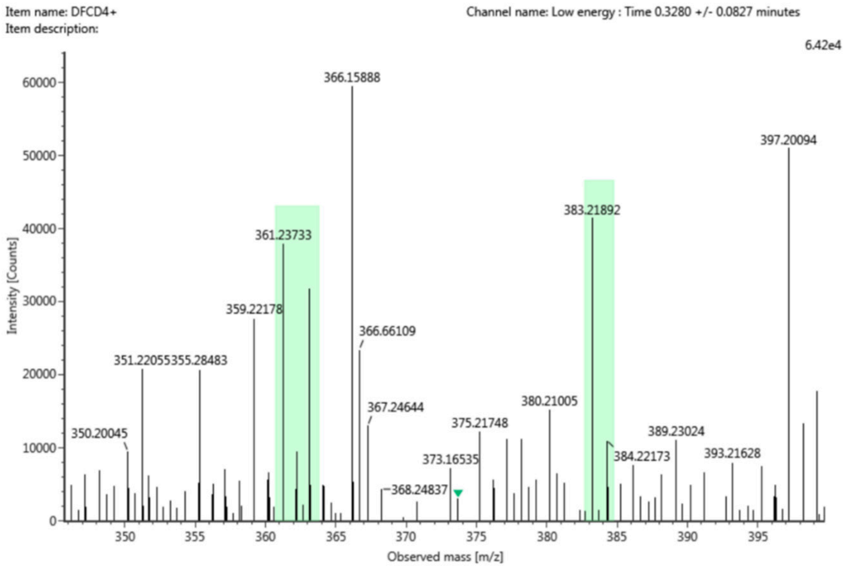

Formula Calculator Results

Formula	Neutral mass (Da)	Observed m/z	Observed RT (min)	Mass error (mDa)	Mass error (ppm)	Adducts
C22H32O4	360.23006	383.2189	0.33	-0.4	-0.9	+Na, +H

Figure S30. HR-ESI-MS spectrum of compound 3.

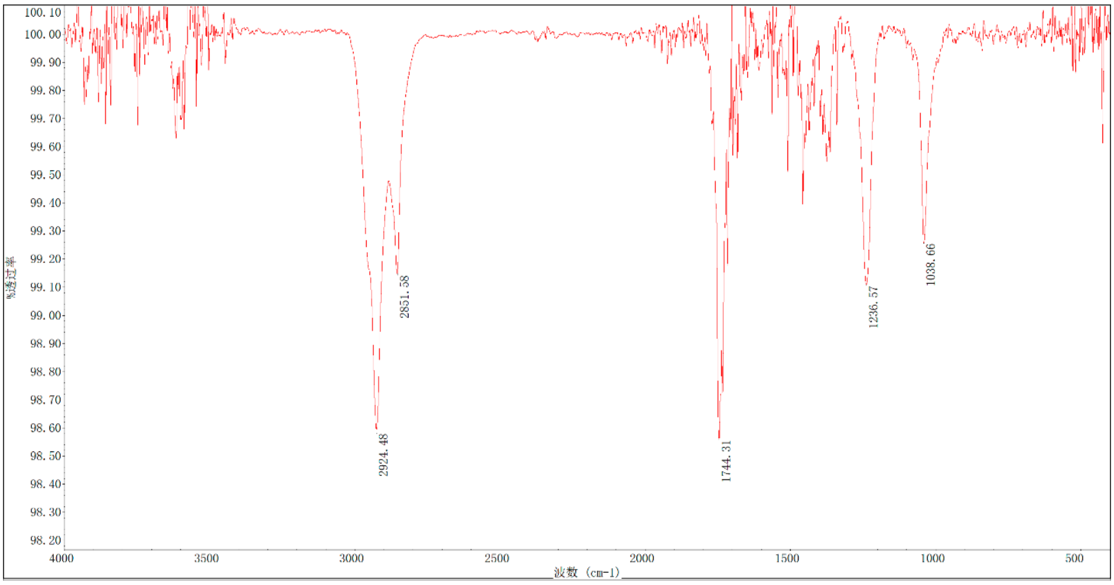

Figure S31. IR spectrum of compound 3.

Functional	Solvent?	Basis Set	Type of Data			
mPW1PW91	PCM	6-311+G(d,p)	Shielding Tensors			
	Isomer 1	Isomer 2	Isomer 3	Isomer 4	Isomer 5	Isomer 6
sDP4+ (H data)	0.00%	100.00%	–	–	–	–
sDP4+ (C data)	13.20%	86.80%	–	–	–	–
sDP4+ (all data)	0.00%	100.00%	–	–	–	–
uDP4+ (H data)	0.10%	99.90%	–	–	–	–
uDP4+ (C data)	25.11%	74.89%	–	–	–	–
uDP4+ (all data)	0.03%	99.97%	–	–	–	–
DP4+ (H data)	0.00%	100.00%	–	–	–	–
DP4+ (C data)	4.85%	95.15%	–	–	–	–
DP4+ (all data)	0.00%	100.00%	–	–	–	–

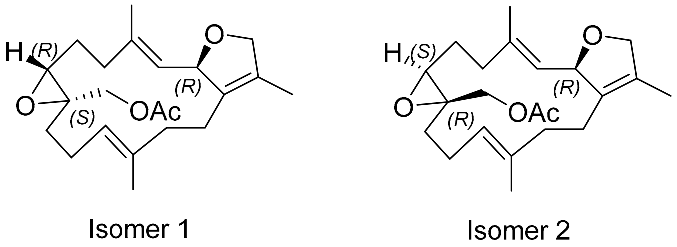

Figure S32. DP4+ results of compound 3 (Isomer 1: 2*R, 7*R**, 8*S**; Isomer 2: 2*R**, 7*S**, 8*R**).**

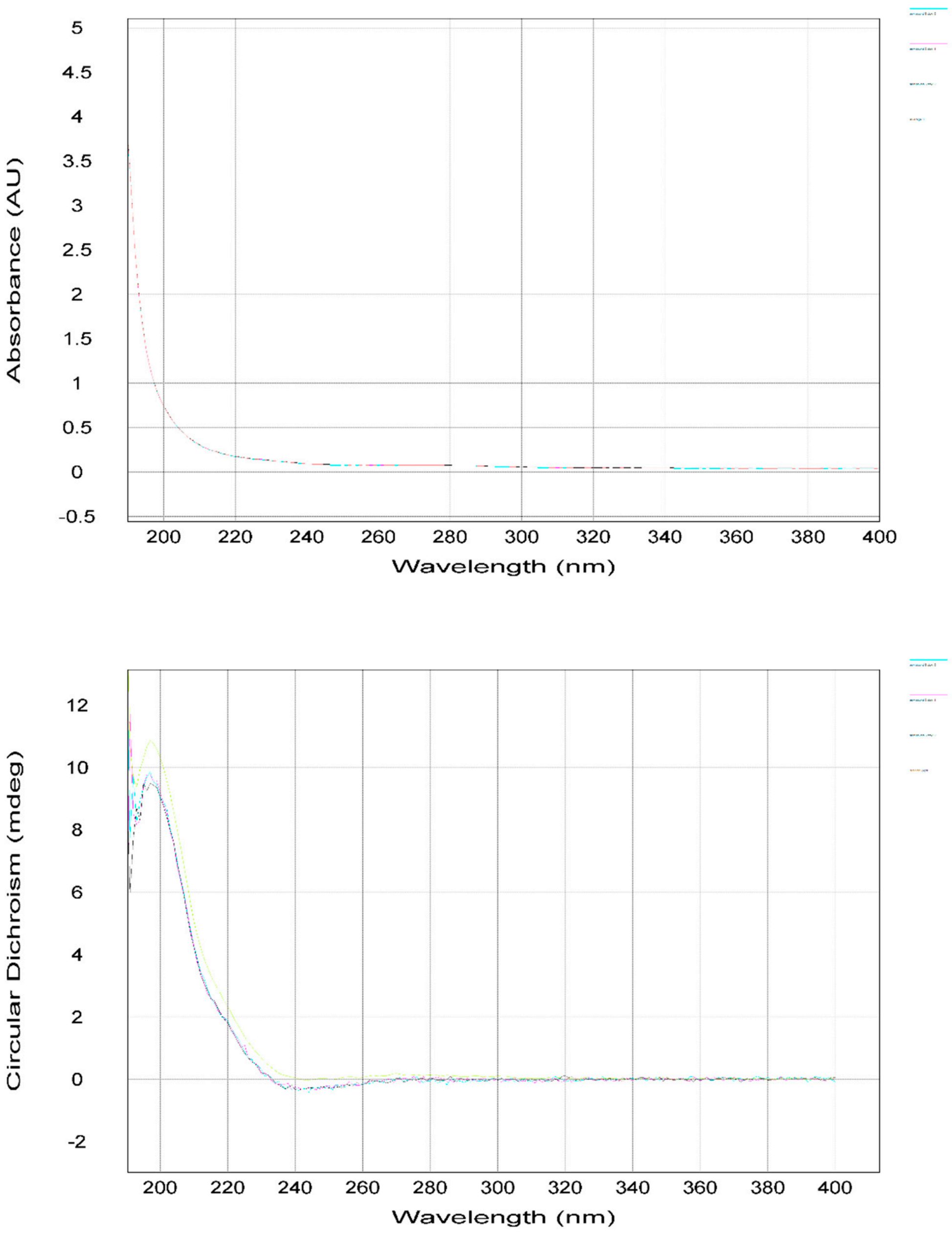

Figure S33. UV and CD spectrum of compound 3.

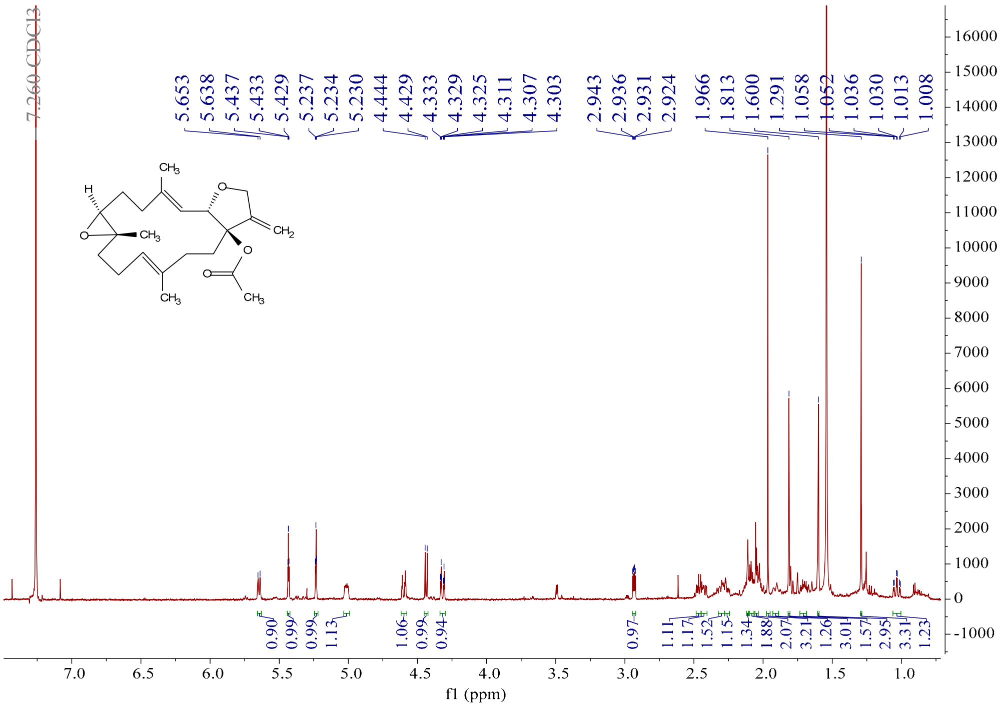

Figure S34. ^{1}H NMR spectrum (600 MHz) of compound 4 in CDCl$_3$.

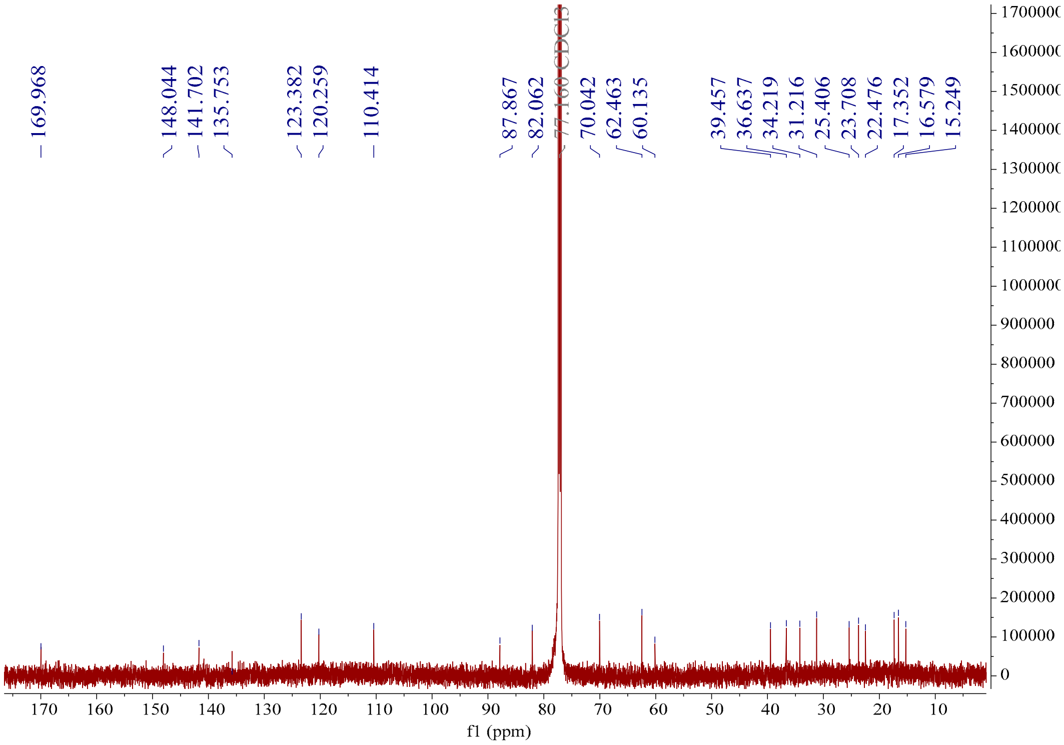

Figure S35. ^{13}C NMR spectrum (600 MHz) of compound 4 in CDCl$_3$.

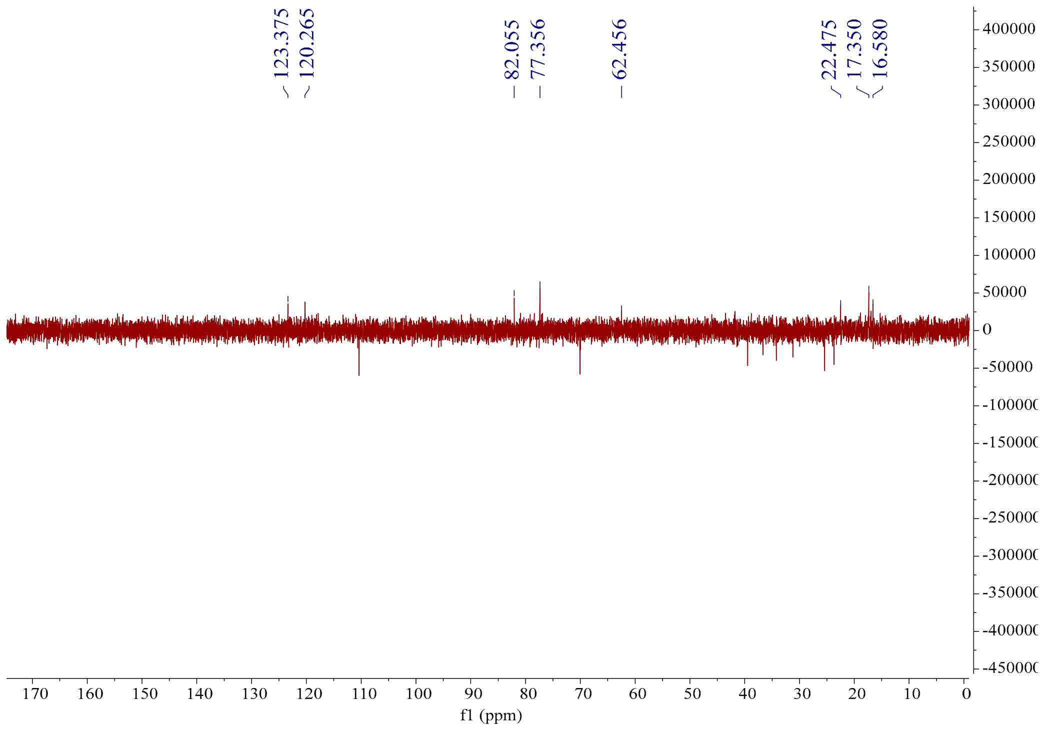

Figure S36. DEPT spectrum (150 MHz) of compound 4 in $CDCl_3$.

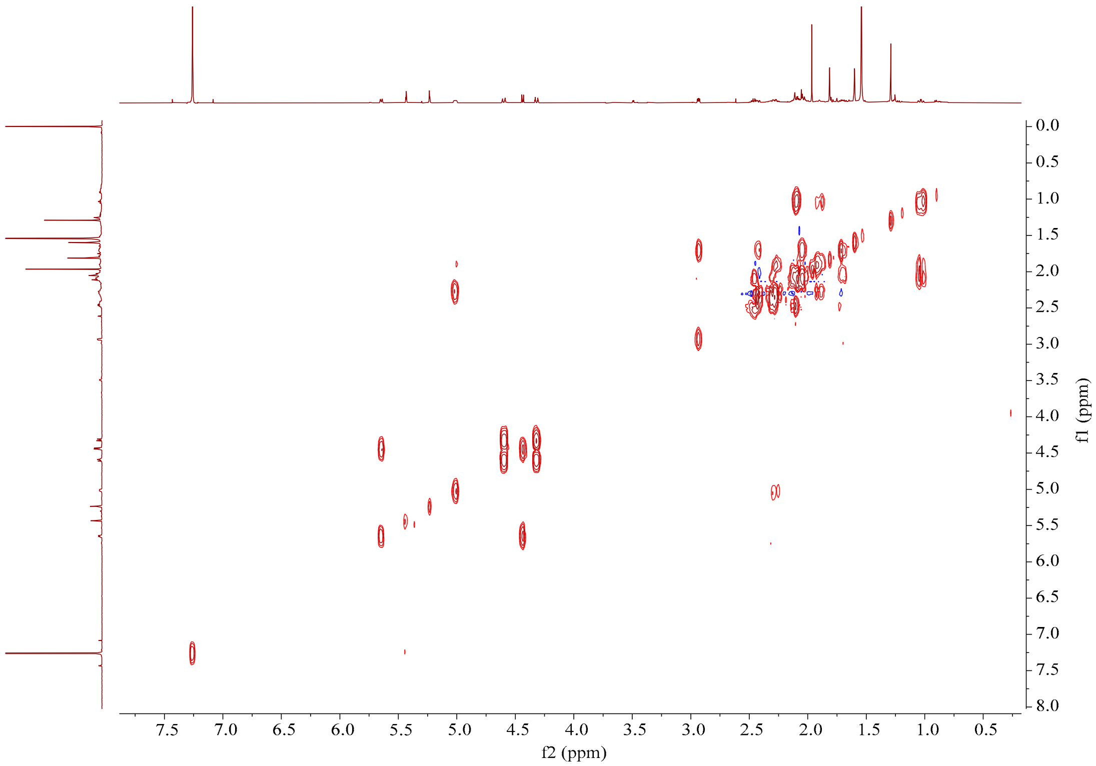

Figure S37. ^{1}H-^{1}H COSY spectrum (600 MHz) of compound 4 in $CDCl_3$.

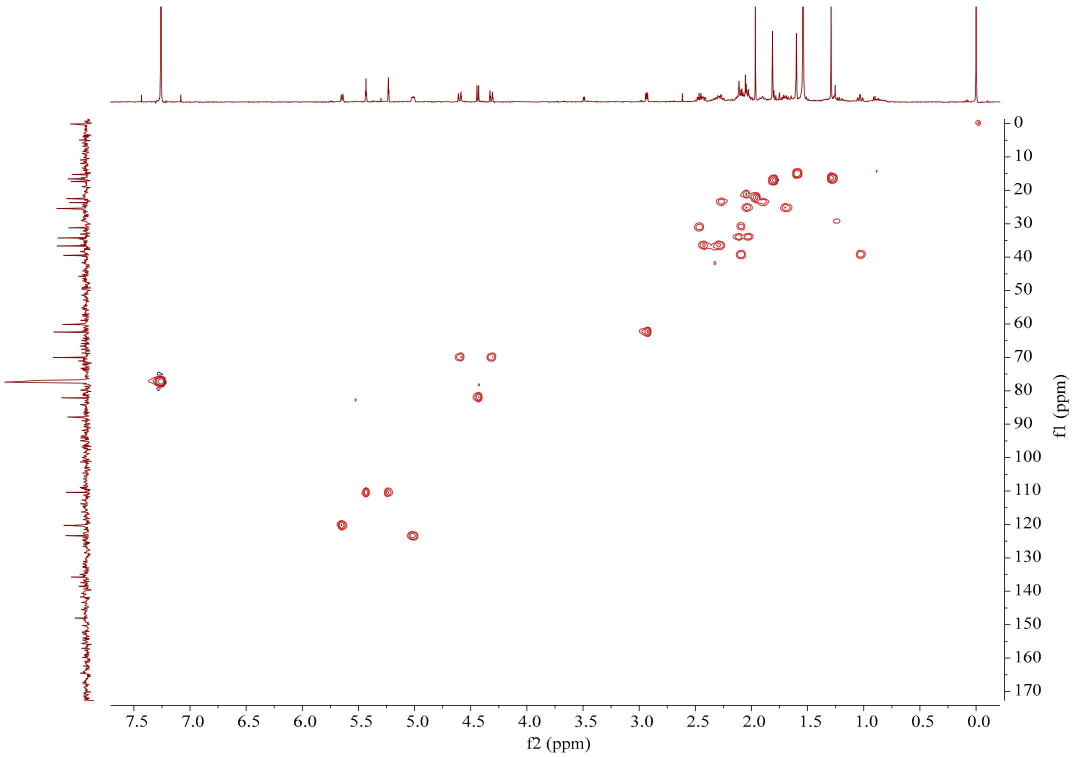

Figure S38. HSQC spectrum (600 MHz) of compound 4 in $CDCl_3$.

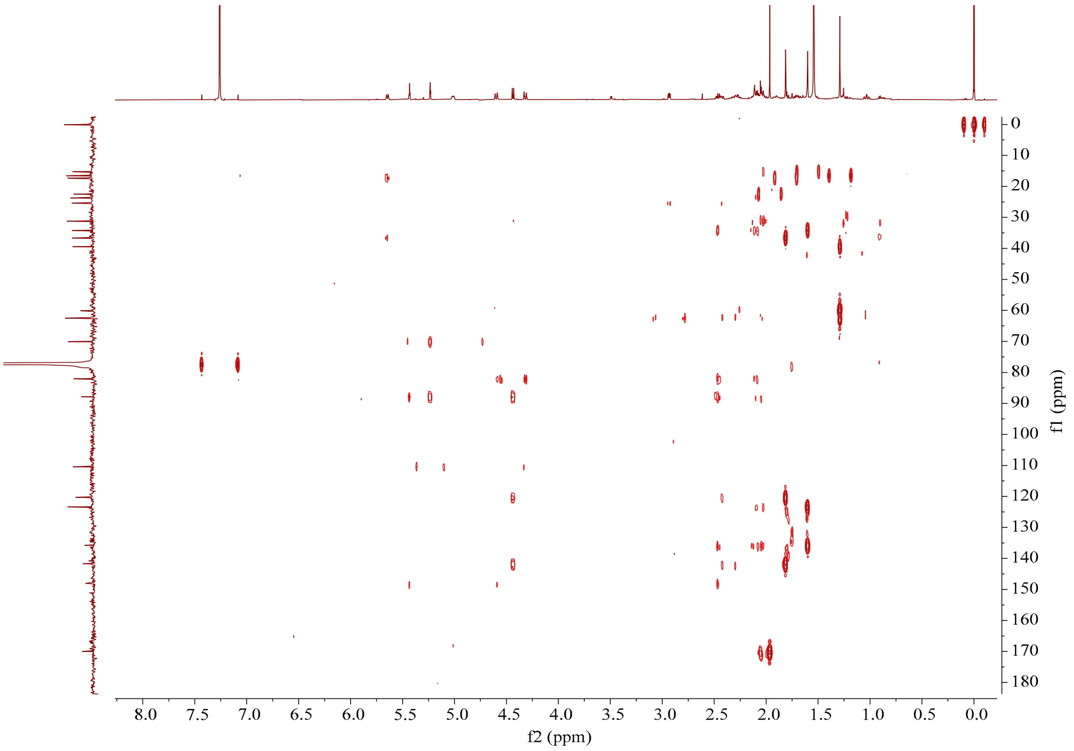

Figure S39. HMBC spectrum (600 MHz) of compound 4 in $CDCl_3$.

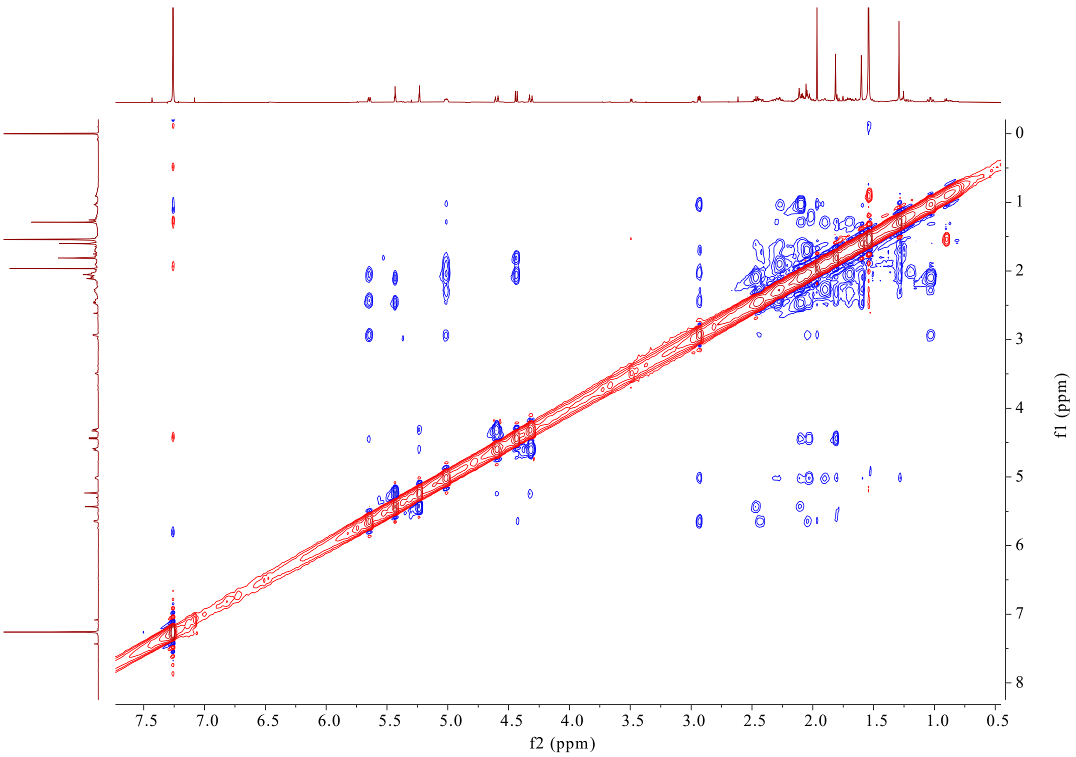

Figure S40. NOESY spectrum (600 MHz) of compound 4 in $CDCl_3$.

MS spectra

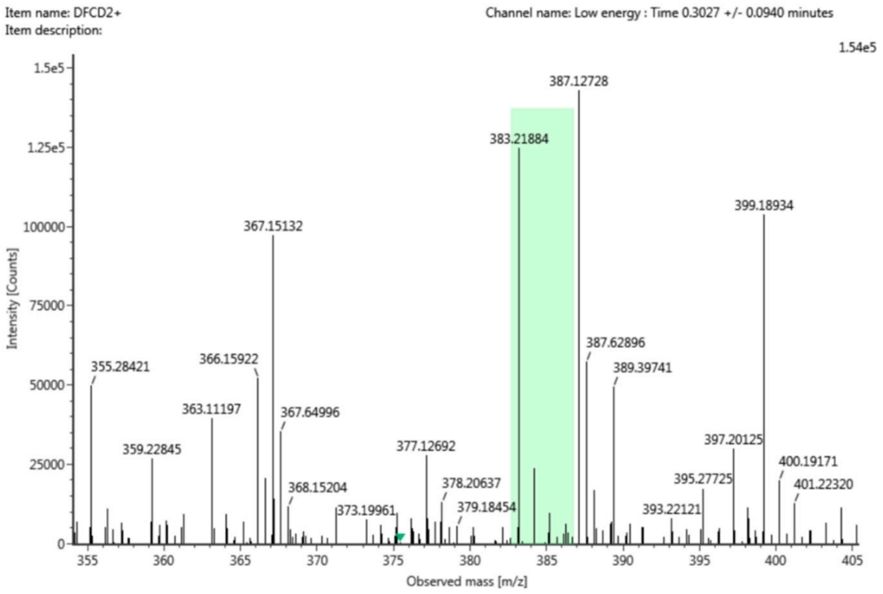

Formula Calculator Results

Formula	Neutral mass (Da)	Observed m/z	Observed RT (min)	Mass error (mDa)	Mass error (ppm)	Adducts
C22H32O4	360.23006	383.2188	0.30	-0.4	-1.1	+Na

Figure S41. HR-ESI-MS spectrum of compound 4.

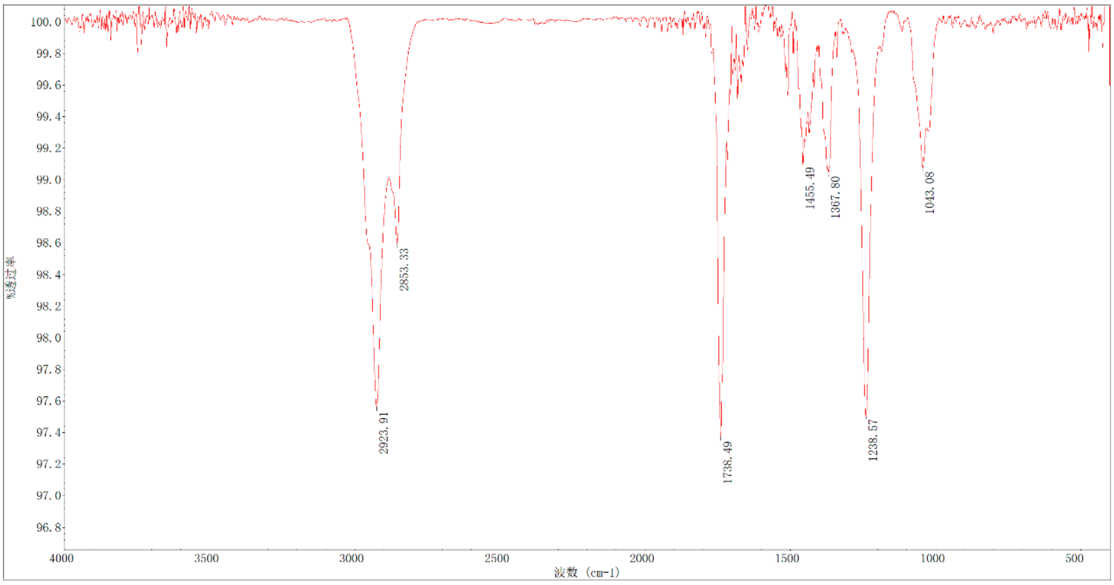

Figure S42. IR spectrum of compound 4.

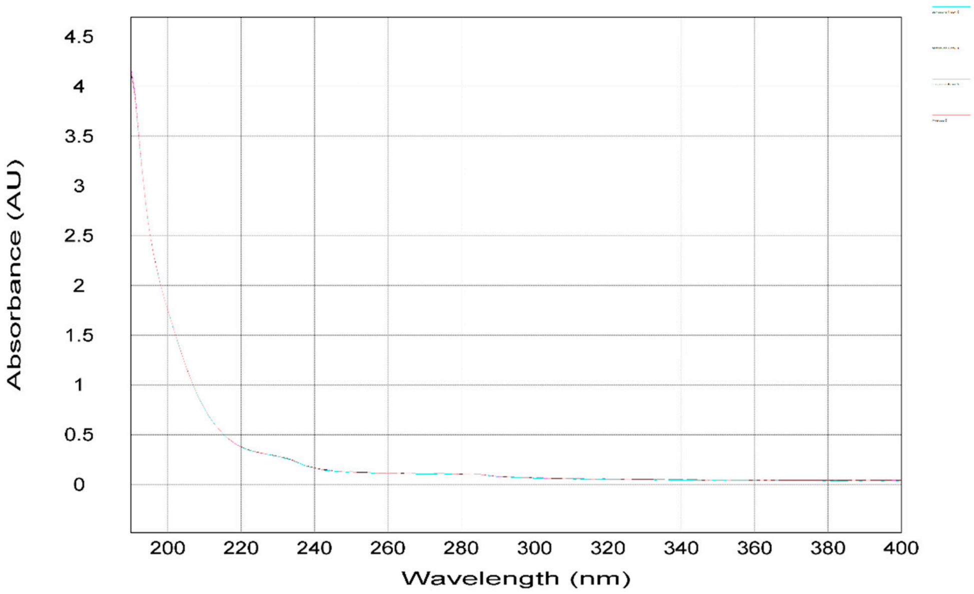

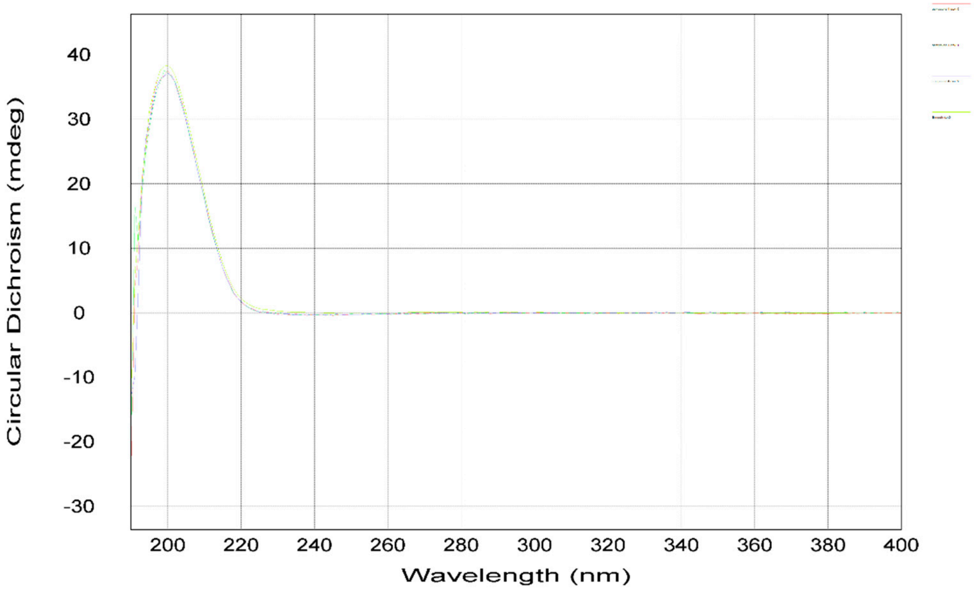

Figure S43. UV and CD spectrum of compound 4.

Functional	Solvent?	Basis Set	Type of Data
mPW1PW91	PCM	6-311+G(d,p)	Shielding Tensors

	Isomer 1	Isomer 2	Isomer 3	Isomer 4	Isomer 5	Isomer 6
sDP4+ (H data)	0.00%	0.00%	0.01%	99.99%	–	–
sDP4+ (C data)	0.00%	0.00%	0.00%	100.00%	–	–
sDP4+ (all data)	0.00%	0.00%	0.00%	100.00%	–	–
uDP4+ (H data)	0.00%	1.00%	0.02%	98.99%	–	–
uDP4+ (C data)	0.05%	0.01%	0.02%	99.92%	–	–
uDP4+ (all data)	0.00%	0.00%	0.00%	100.00%	–	–
DP4+ (H data)	0.00%	0.00%	0.00%	100.00%	–	–
DP4+ (C data)	0.00%	0.00%	0.00%	100.00%	–	–
DP4+ (all data)	0.00%	0.00%	0.00%	100.00%	–	–

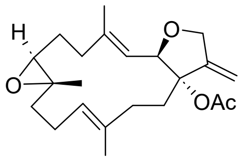

Isomer 1

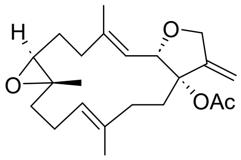

Isomer 2

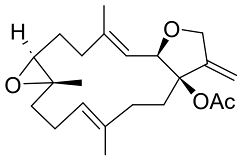

Isomer 3

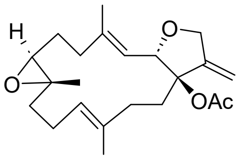

Isomer 4

Figure S44. DP4+ results of compound 4 (Isomer 1: 1*S, 2*R**, 7*S**. 8*S**; Isomer 2: 1*S**, 2*S**, 7*S**. 8*S**; Isomer 3: 1*R**, 2*R**, 7*S**. 8*S**; Isomer 4: 1*R**, 2*S**, 7*S**. 8*S**).**

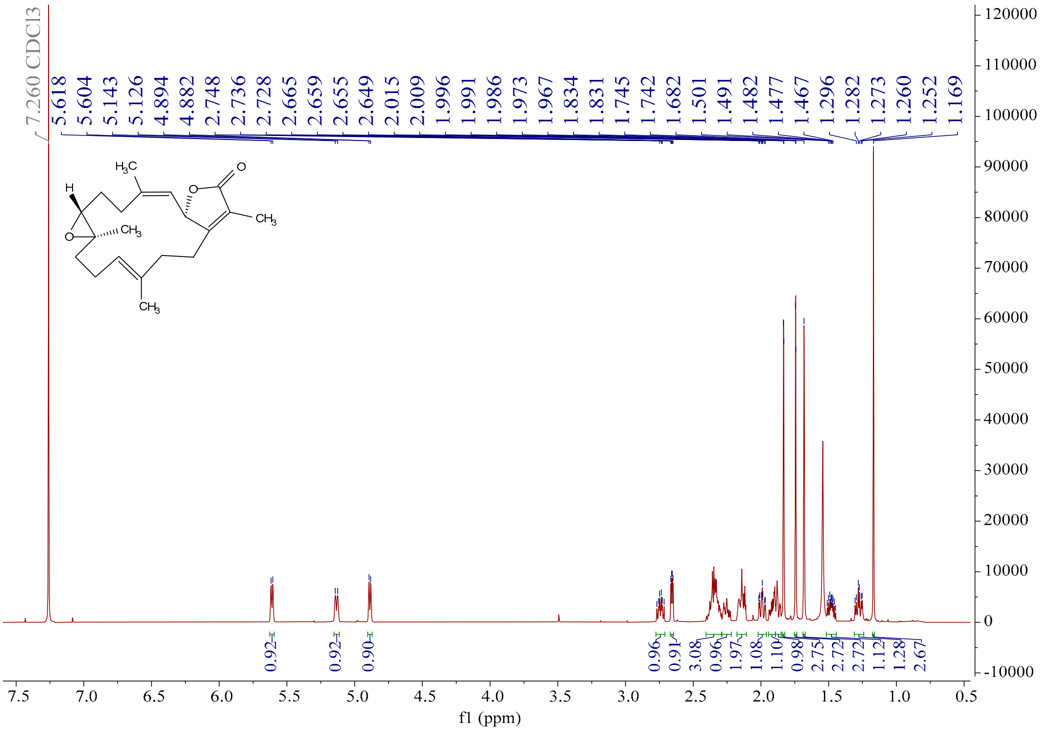

Figure S45. ^{1}H NMR spectrum (600 MHz) of compound 5 in $CDCl_3$.

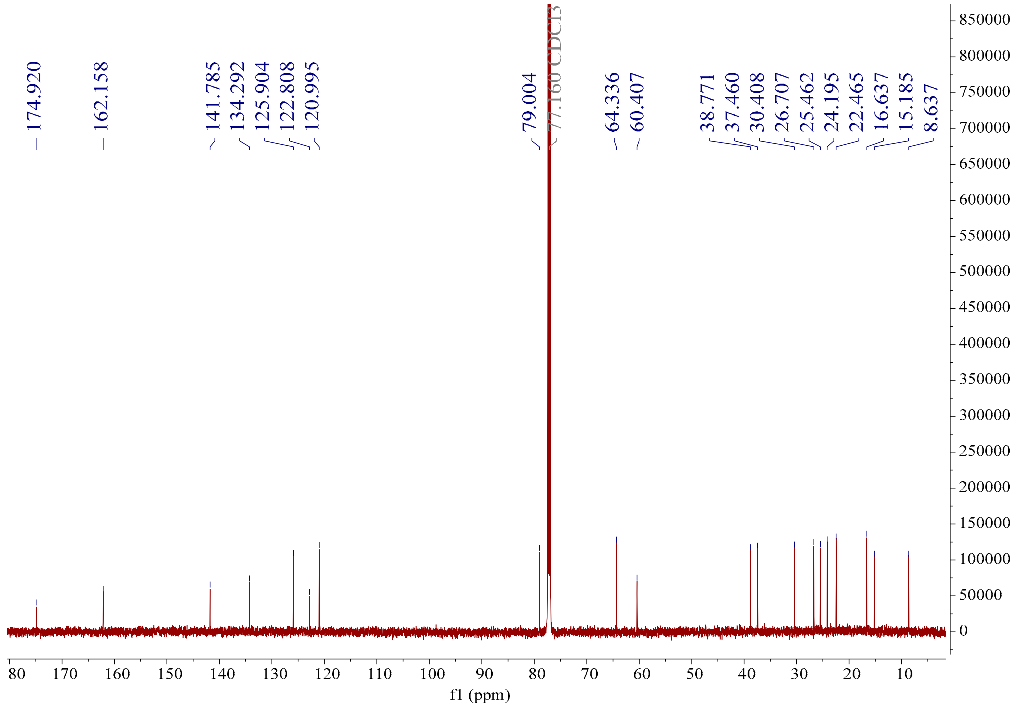

Figure S46. ^{13}C NMR spectrum (600 MHz) of compound 5 in $CDCl_3$.

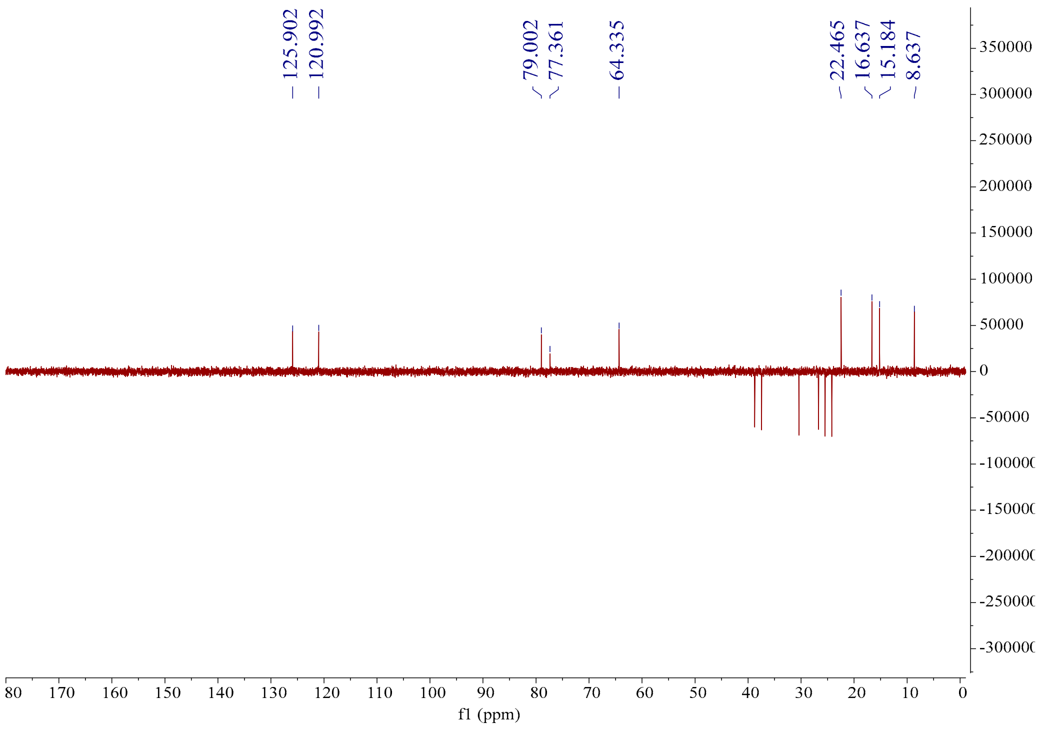

Figure S47. DEPT spectrum (150 MHz) of compound 5 in $CDCl_3$.

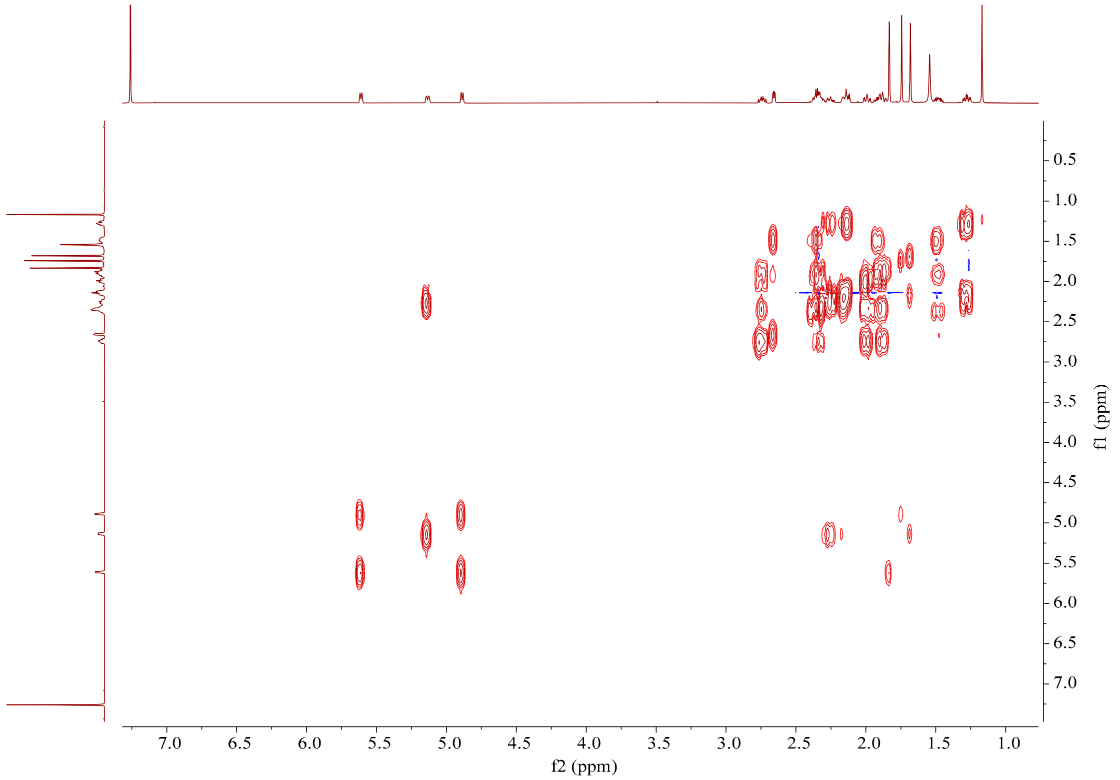

Figure S48. ^1H-^1H COSY spectrum (600 MHz) of compound 5 in $CDCl_3$.

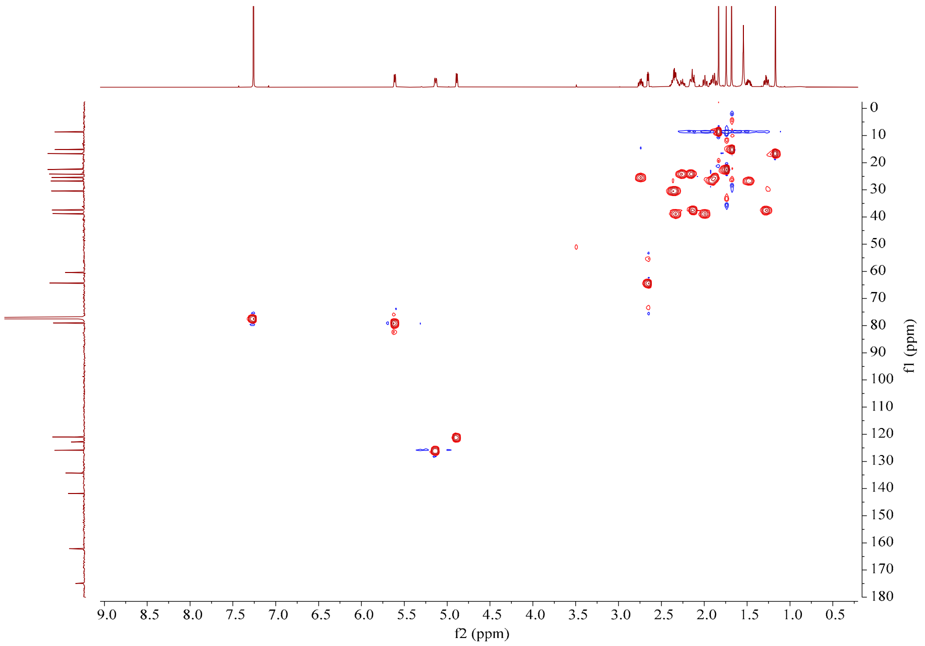

Figure S49. HSQC spectrum (600 MHz) of compound 5 in $CDCl_3$.

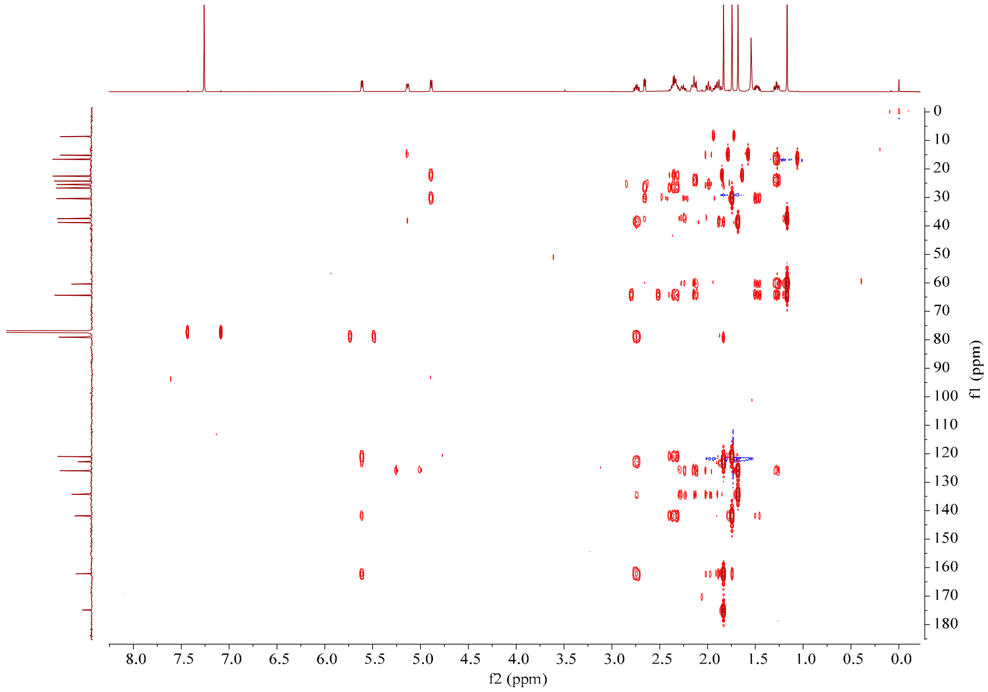

Figure S50. HMBC spectrum (600 MHz) of compound 5 in $CDCl_3$.

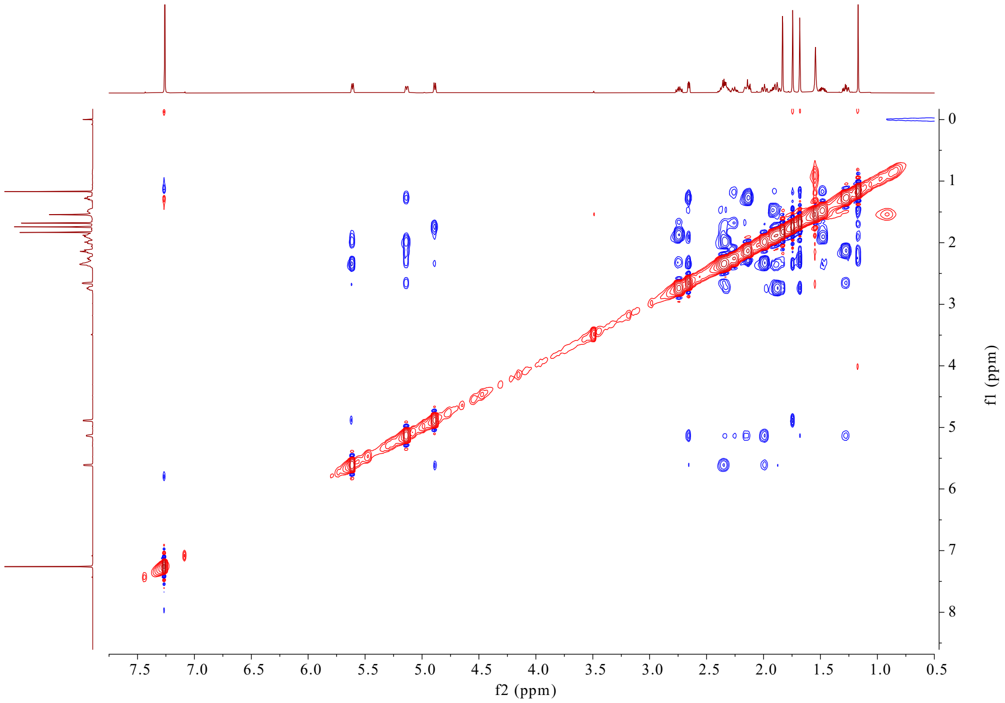

Figure S51. NOESY spectrum (600 MHz) of compound 5 in $CDCl_3$.

MS spectra

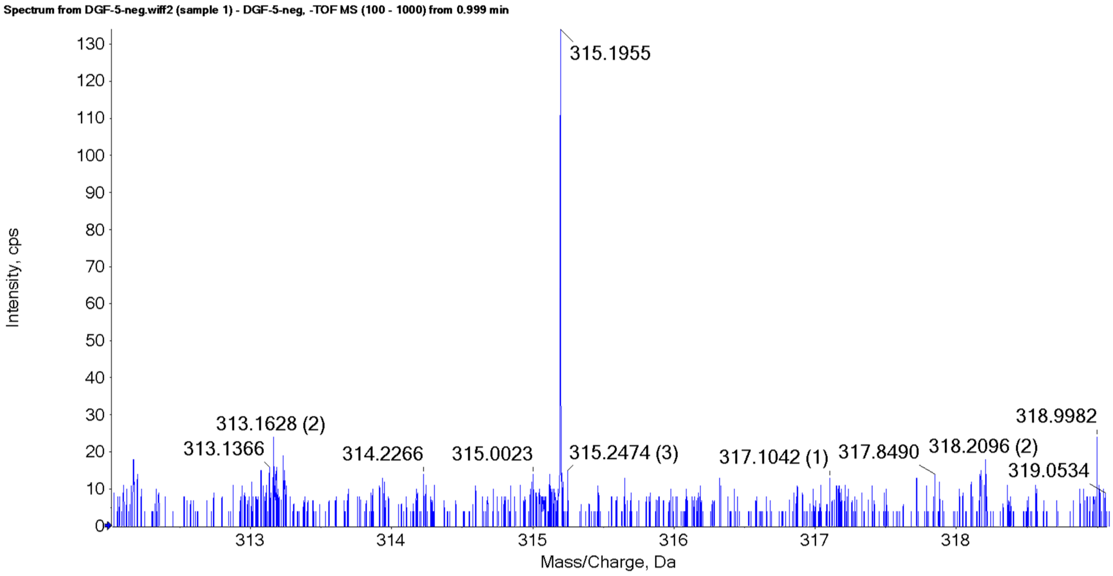

Formula Calculator Results

Measured m/z	Cal m/z	Error(mmu)	Error(ppm)	Ion Formula	Ion
315.1955	315.1955	0	0	C20H27O3	$[M-H]^-$

Figure S52. HR-ESI-MS spectrum of compound 5.

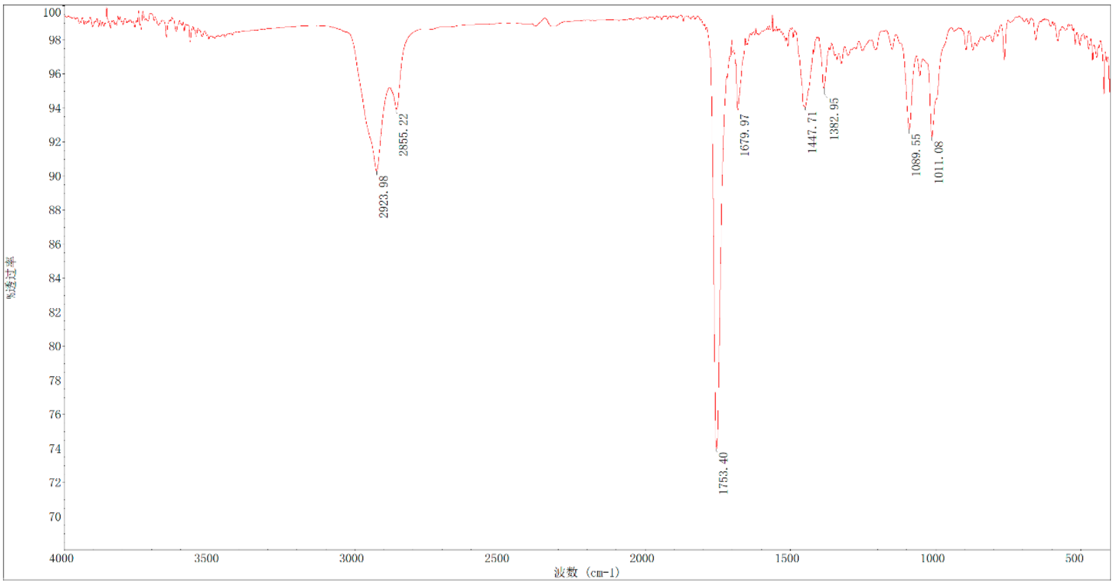

Figure S53. IR spectrum of compound 5.

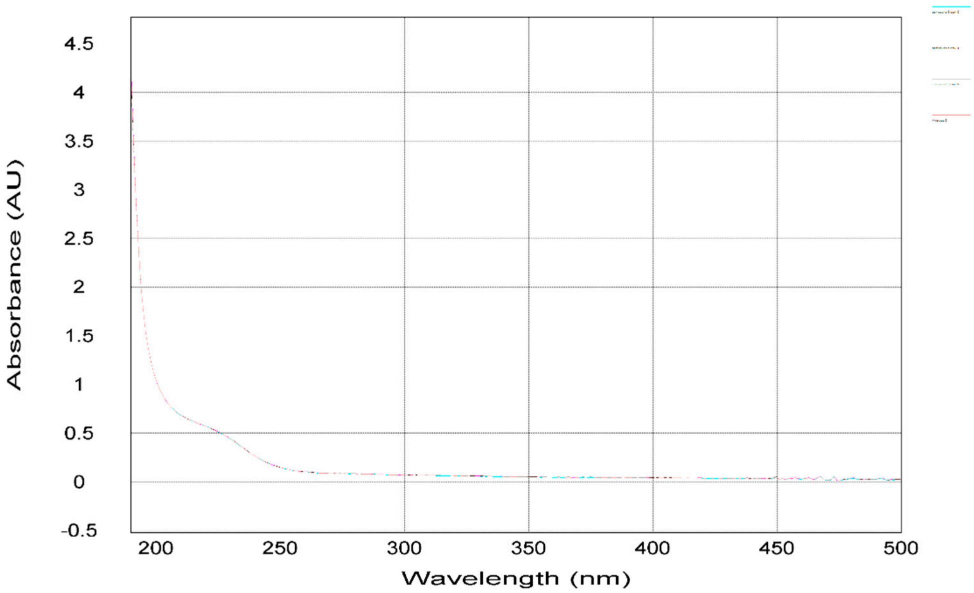

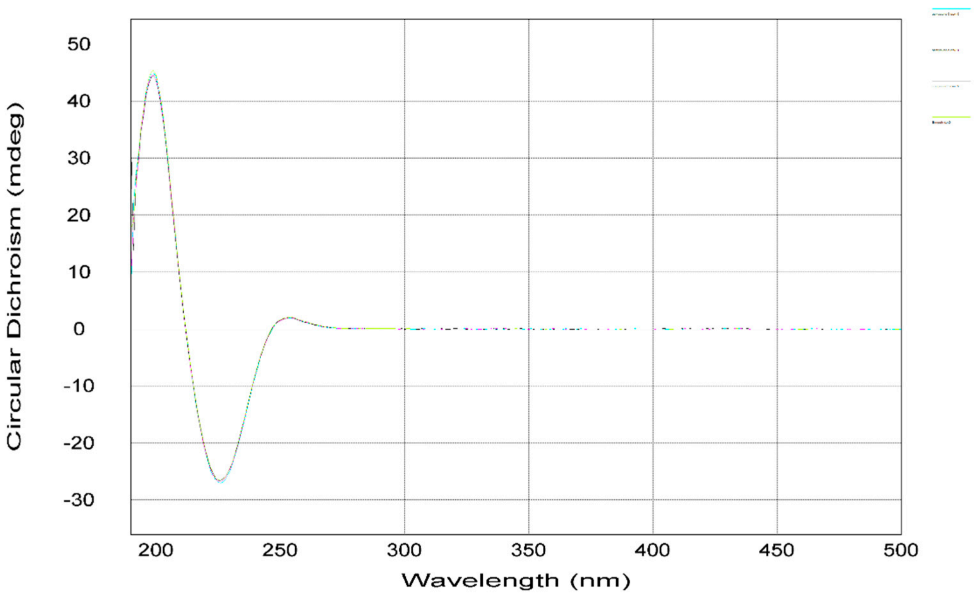

Figure S54. UV and CD spectrum of compound 5.

Functional	Solvent?		Basis Set		Type of Data	
mPW1PW91	PCM		6-311G(d,p)		Shielding Tensors	
	Isomer 1	Isomer 2	Isomer 3	Isomer 4	Isomer 5	Isomer 6
sDP4+ (H data)	0.01%	99.99%	-	-	-	-
sDP4+ (C data)	77.51%	22.49%	-	-	-	-
sDP4+ (all data)	0.04%	99.96%	-	-	-	-
uDP4+ (H data)	0.00%	100.00%	-	-	-	-
uDP4+ (C data)	0.00%	100.00%	-	-	-	-
uDP4+ (all data)	0.00%	100.00%	-	-	-	-
DP4+ (H data)	0.00%	100.00%	-	-	-	-
DP4+ (C data)	0.00%	100.00%	-	-	-	-
DP4+ (all data)	0.00%	100.00%	-	-	-	-

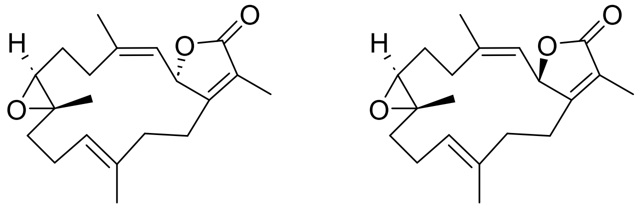

Isomer 1 Isomer 2

Figure S55. DP4+ results of compound 5 (Isomer 1: 2*R, 7*S**, 8*S**; Isomer 2: 2*S**, 7*S**, 8*S**).**

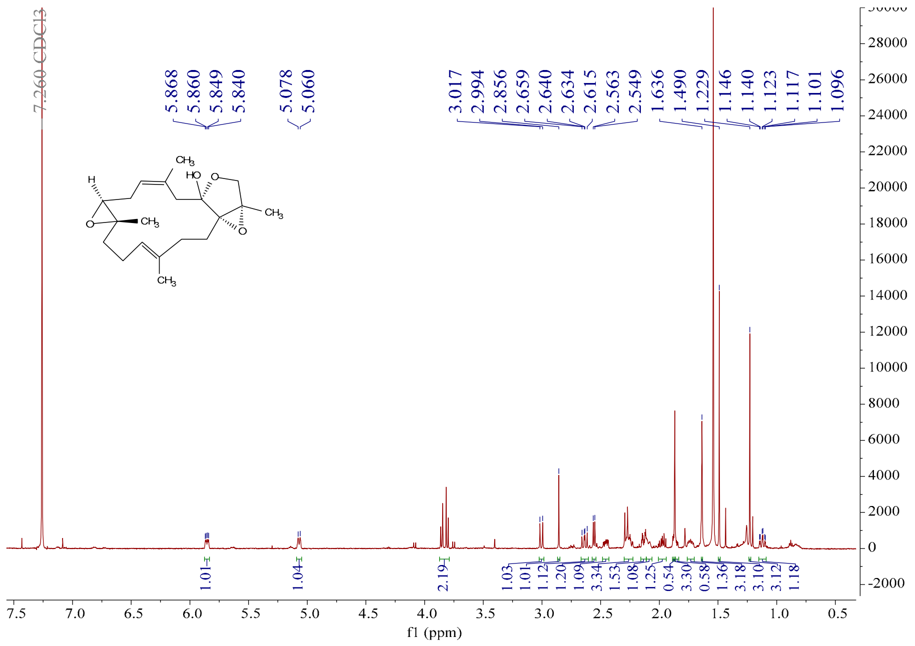

Figure S56. ¹H NMR spectrum (600 MHz) of compound 6 in $CDCl_3$.

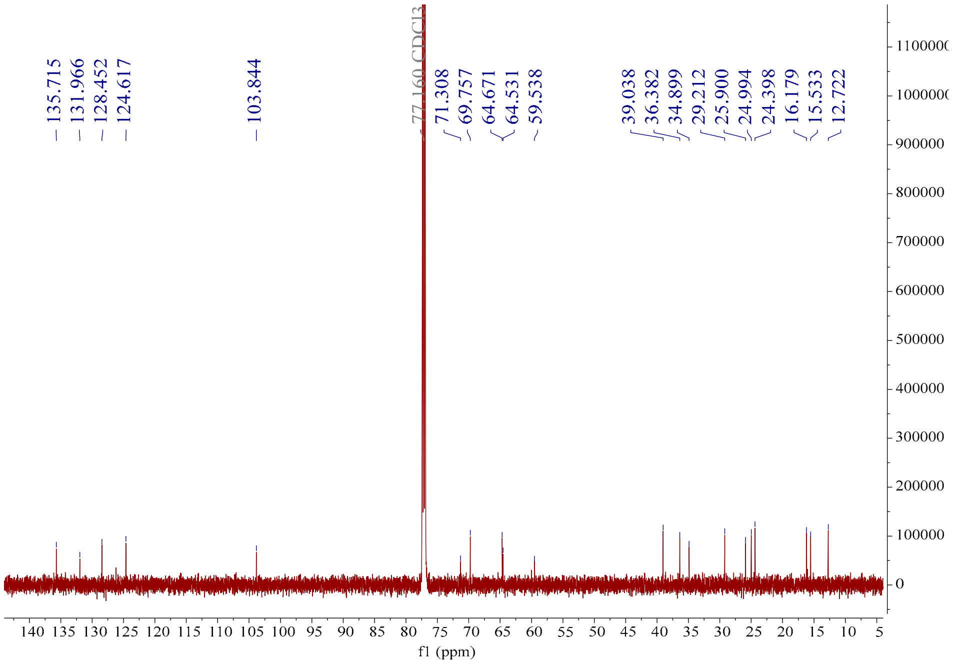

Figure S57. ¹³C NMR spectrum (600 MHz) of compound 6 in $CDCl_3$.

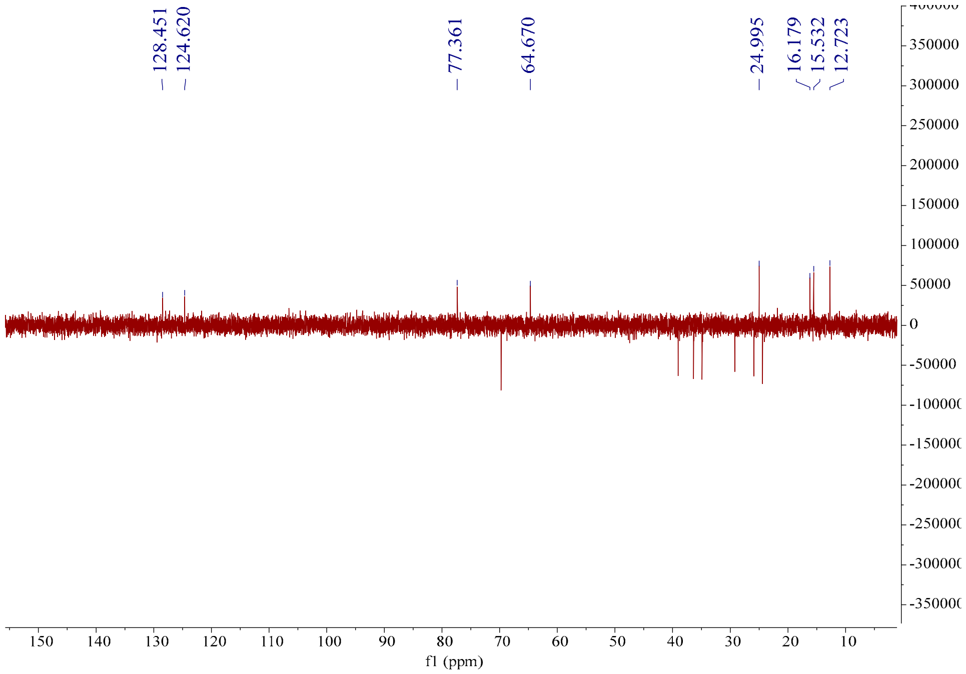

Figure S58. DEPT spectrum (150 MHz) of compound 6 in $CDCl_3$.

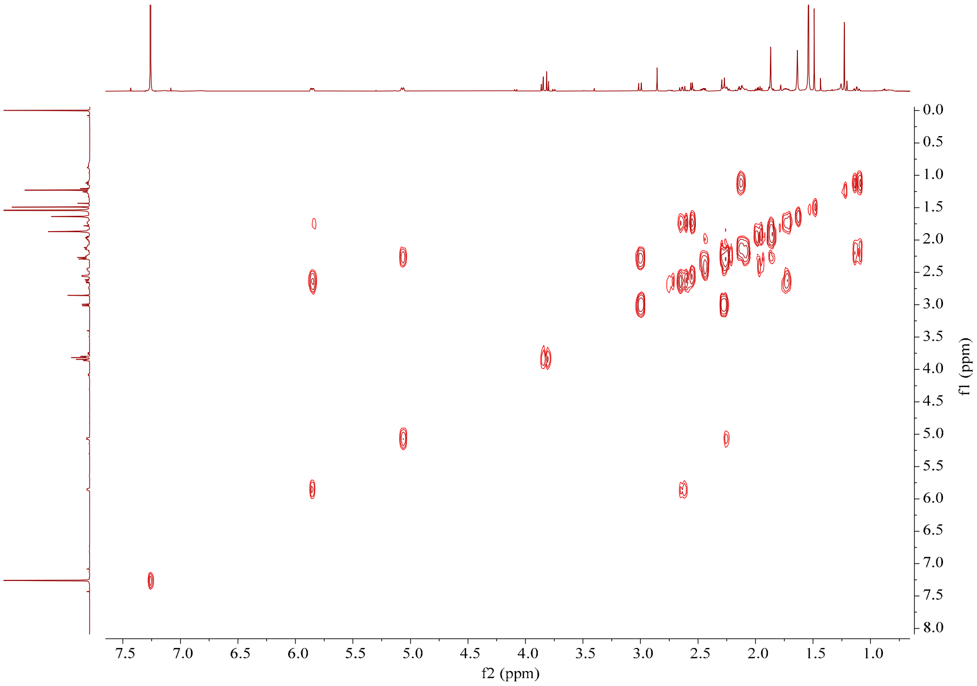

Figure S59. ^{1}H-^{1}H COSY spectrum (600 MHz) of compound 6 in $CDCl_3$.

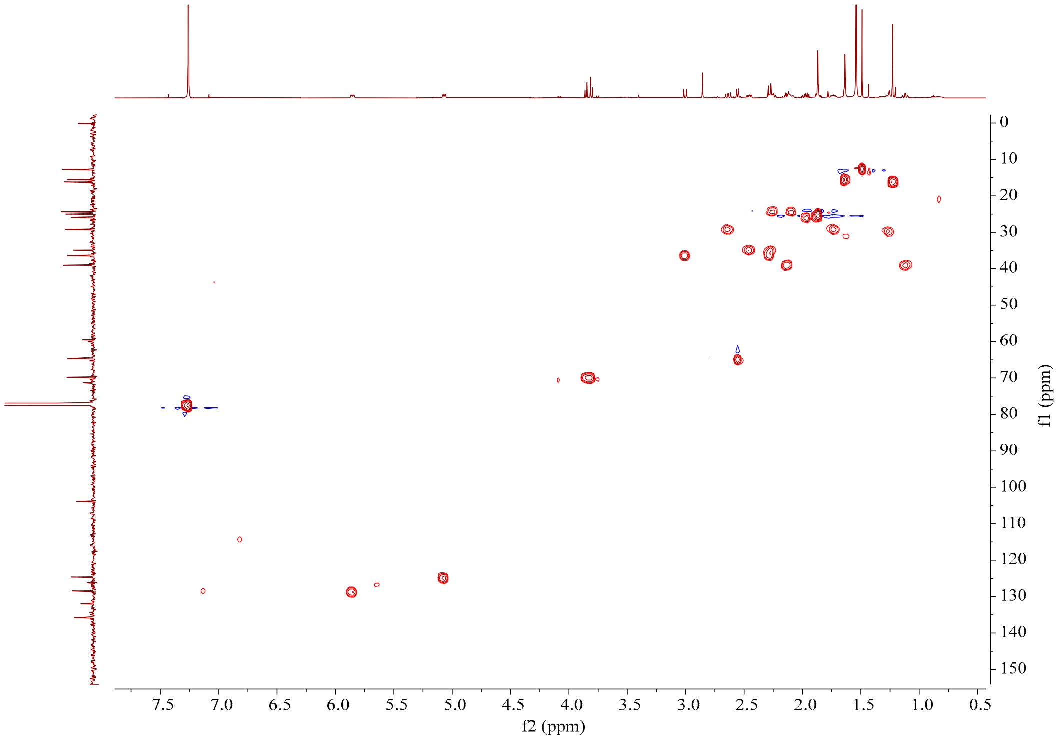

Figure S60. HSQC spectrum (600 MHz) of compound 6 in $CDCl_3$.

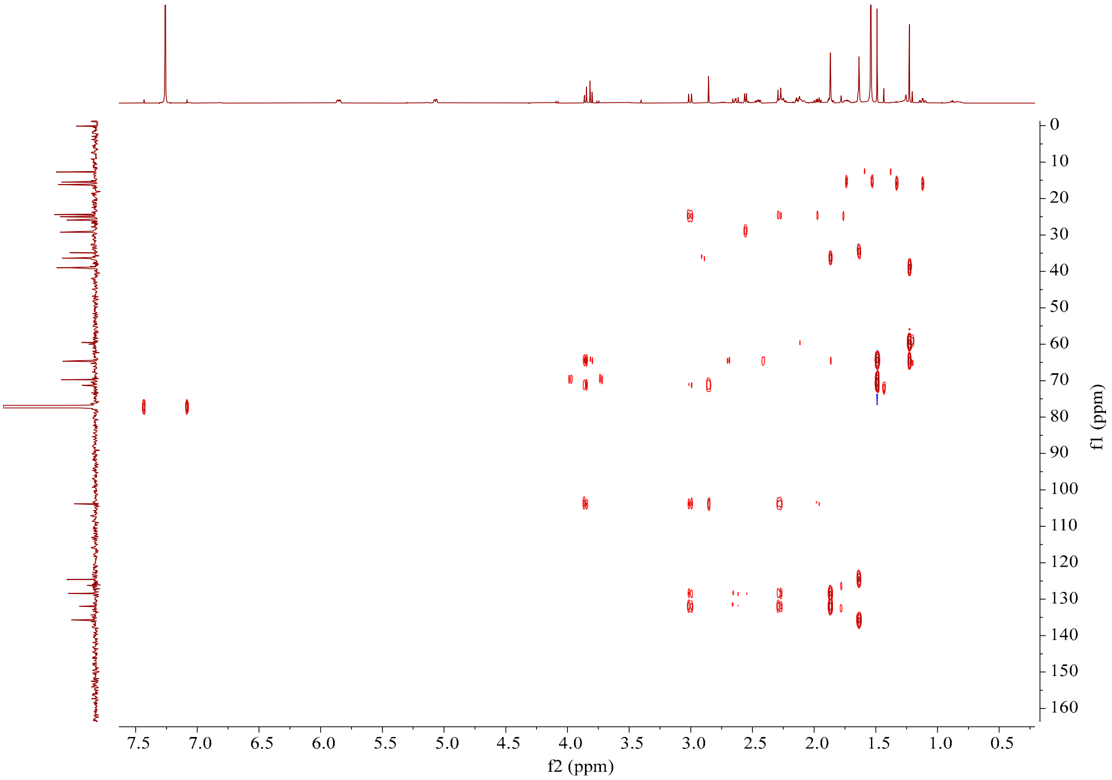

Figure S61. HMBC spectrum (600 MHz) of compound 6 in $CDCl_3$.

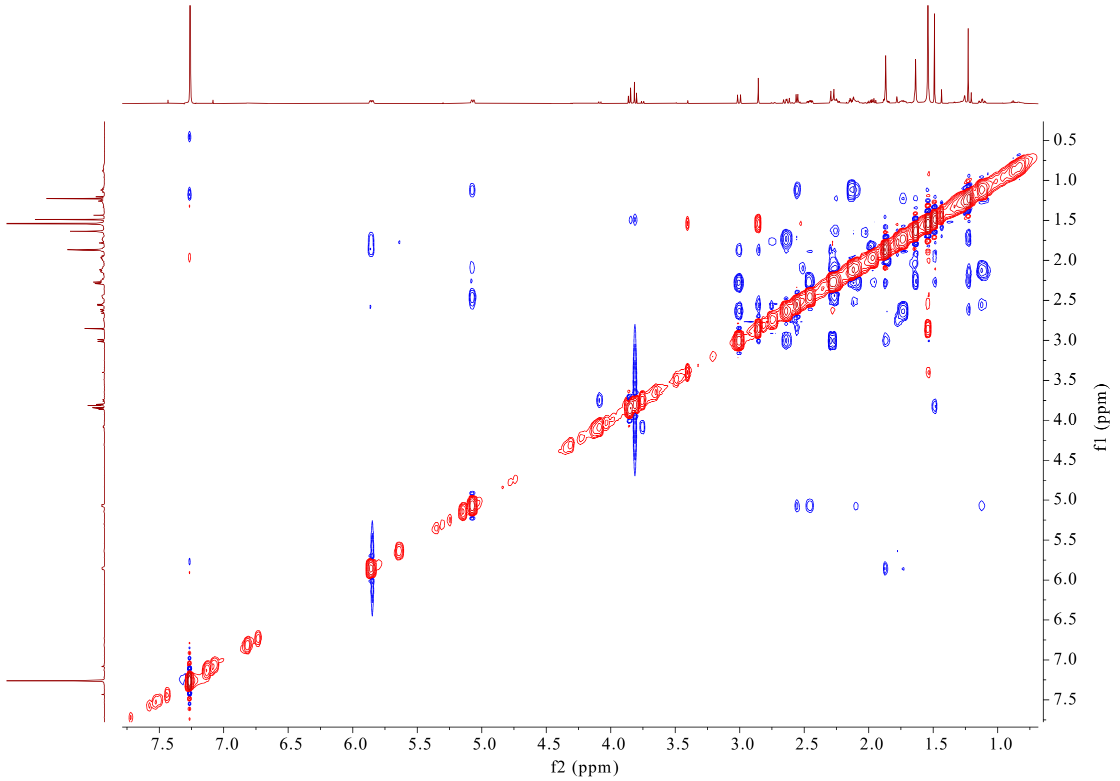

Figure S62. NOESY spectrum (600 MHz) of compound 6 in $CDCl_3$.

MS spectra

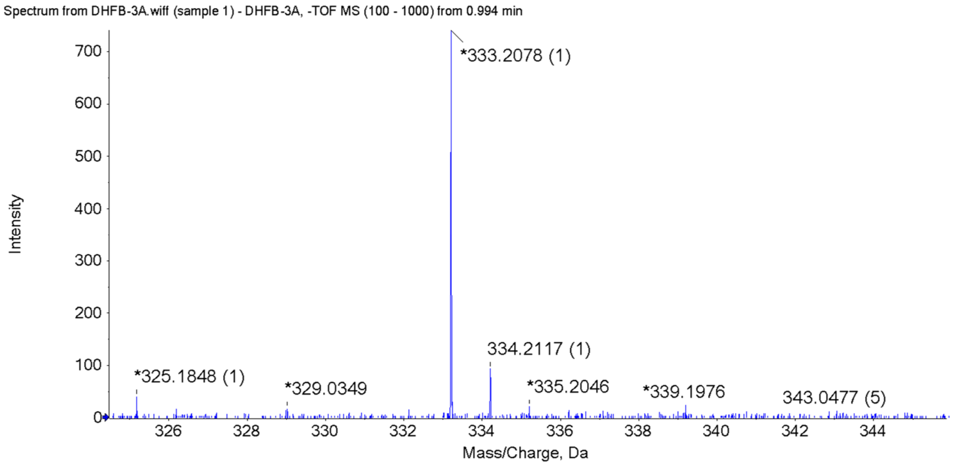

Formula Calculator Results

Measure m/z	Cal m/z	Error(mmu)	Error(ppm)	Ion Formula	Ion
333.2078	333.2066	1.2	3.7	C20H29O4	$[M-H]^-$

Figure S63. HR-ESI-MS spectrum of compound 6.

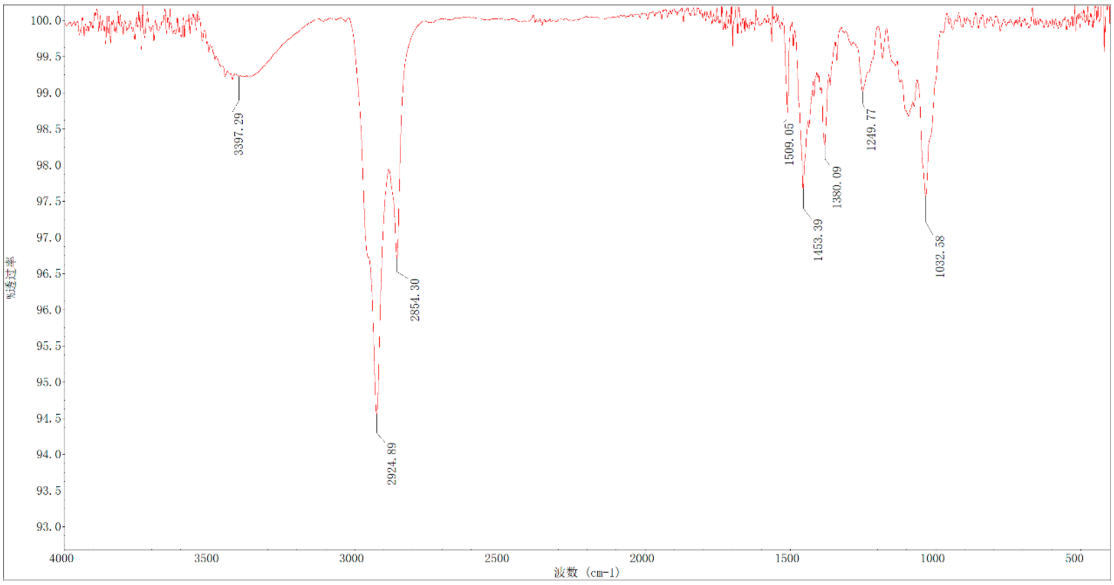

Figure S64. IR spectrum of compound 6.

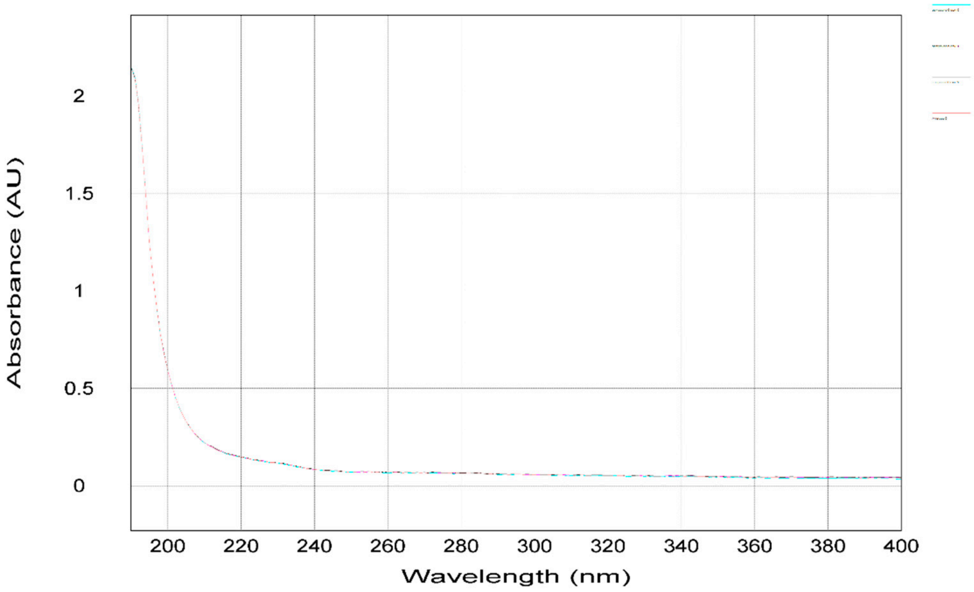

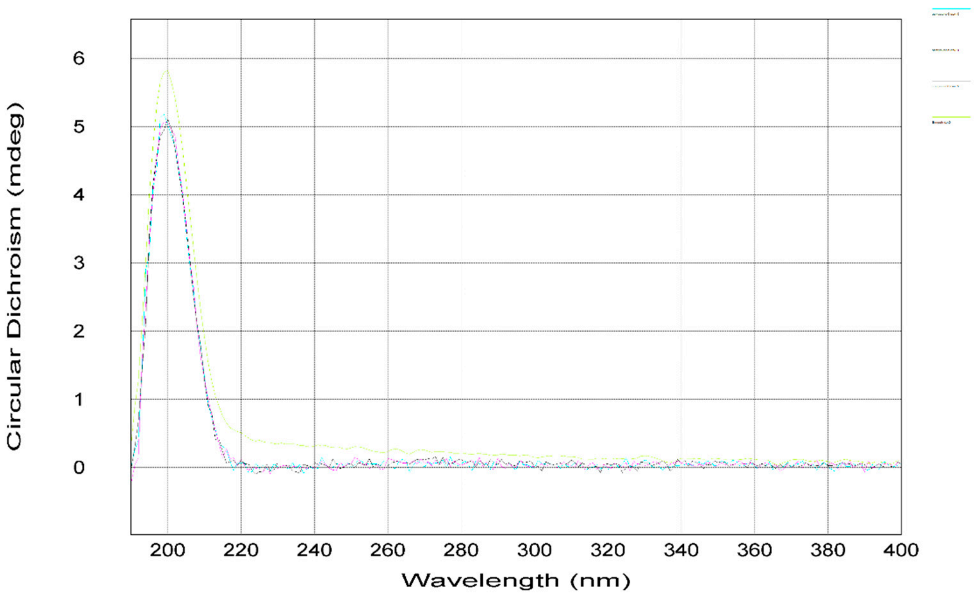

Figure S65. UV and CD spectrum of compound 6.

Functional	Solvent?	Basis Set	Type of Data
mPW1PW91	PCM	6-311G(d,p)	Shielding Tensors

	Isomer 1	Isomer 2	Isomer 3	Isomer 4	Isomer 5	Isomer 6
sDP4+ (H data)	0.00%	100.00%	0.00%	0.00%	-	-
sDP4+ (C data)	0.02%	61.57%	36.36%	2.06%	-	-
sDP4+ (all data)	0.00%	100.00%	0.00%	0.00%	-	-
uDP4+ (H data)	0.00%	100.00%	0.00%	0.00%	-	-
uDP4+ (C data)	0.00%	90.12%	9.74%	0.13%	-	-
uDP4+ (all data)	0.00%	100.00%	0.00%	0.00%	-	-
DP4+ (H data)	0.00%	100.00%	0.00%	0.00%	-	-
DP4+ (C data)	0.00%	94.00%	6.00%	0.00%	-	-
DP4+ (all data)	0.00%	100.00%	0.00%	0.00%	-	-

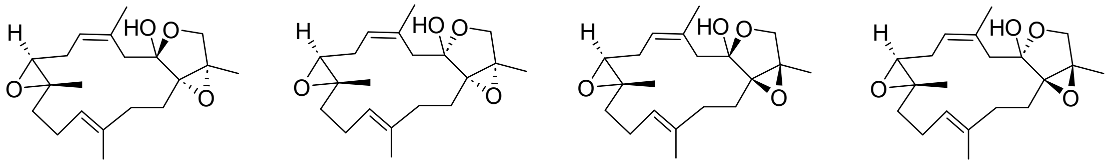

Isomer 1 Isomer 2 Isomer 3 Isomer 4

Figure S66. DP4+ results of compound 6 (Isomer 1: 1*S, 2*R**, 7*S**, 8*S**, 15*S**; Isomer 2: 1*S**, 2*S**, 7*S**, 8*S**, 15*S**; Isomer 3: 1*R**, 2*R**, 7*S**, 8*S**, 15*R**; Isomer 4: 1*R**, 2*S**, 7*S**, 8*S**, 15*R**).**

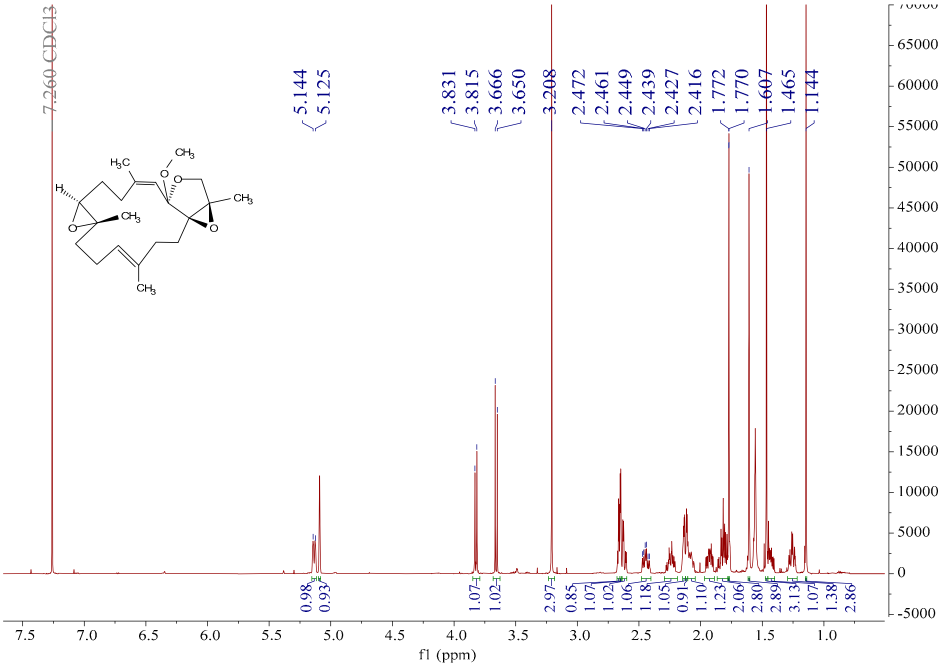

Figure S67. ^{1}H NMR spectrum (600 MHz) of compound 7 in $CDCl_3$.

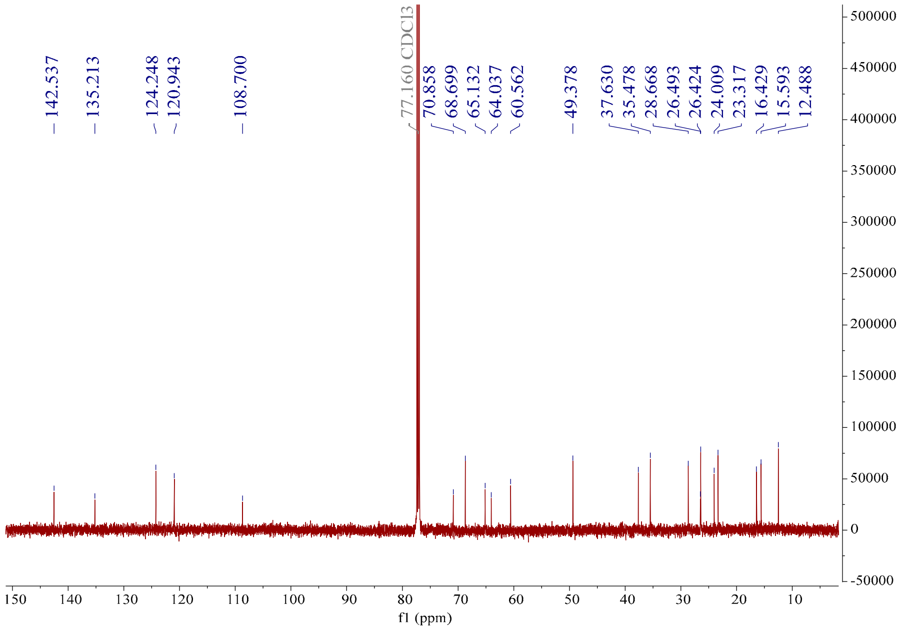

Figure S68. ^{13}C NMR spectrum (600 MHz) of compound 7 in $CDCl_3$.

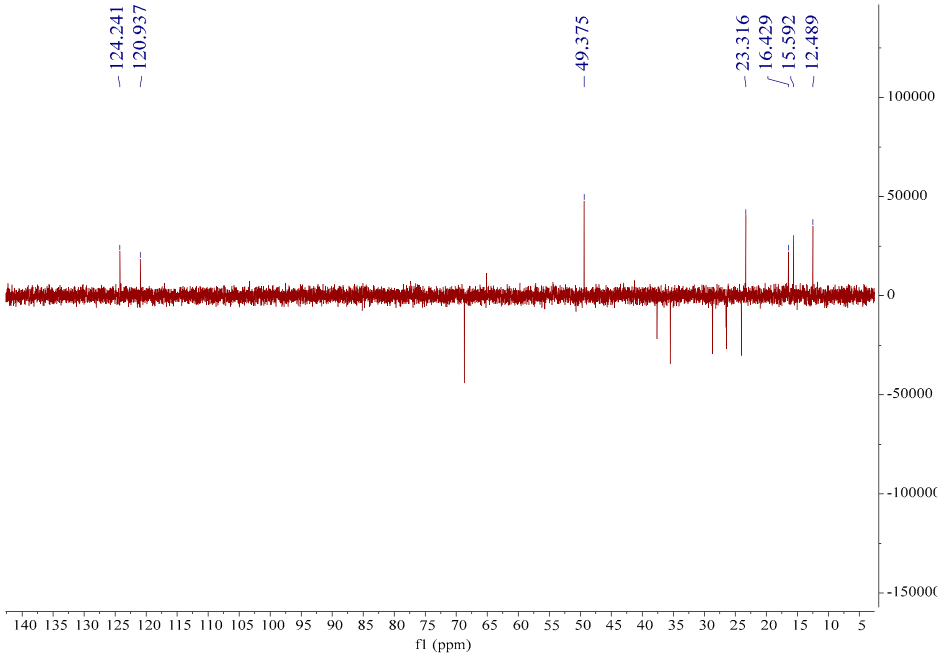

Figure S69. DEPT spectrum (150 MHz) of compound 7 in $CDCl_3$.

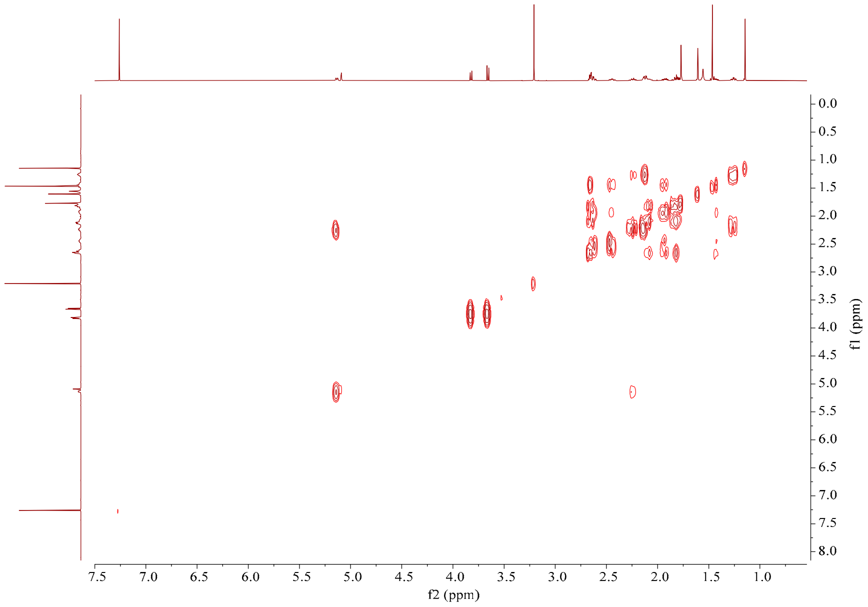

Figure S70. ^1H-^1H COSY spectrum (600 MHz) of compound 7 in $CDCl_3$.

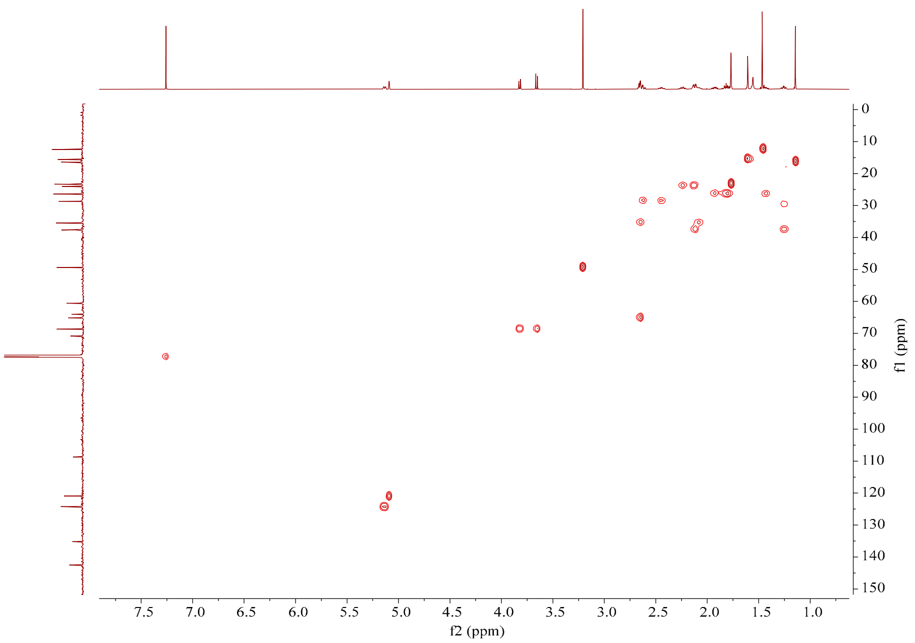

Figure S71. HSQC spectrum (600 MHz) of compound 7 in $CDCl_3$.

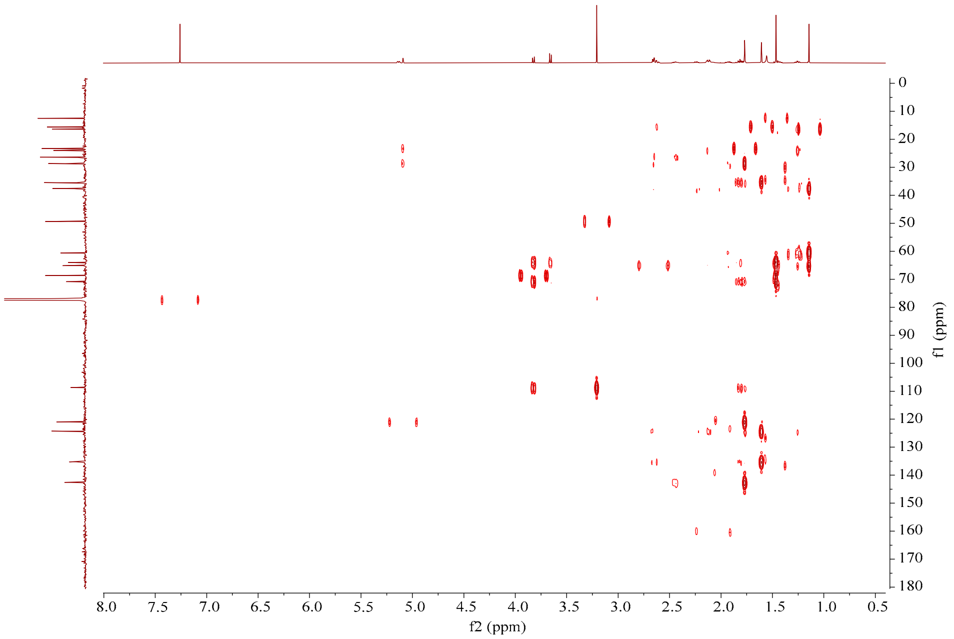

Figure S72. HMBC spectrum (600 MHz) of compound 7 in $CDCl_3$.

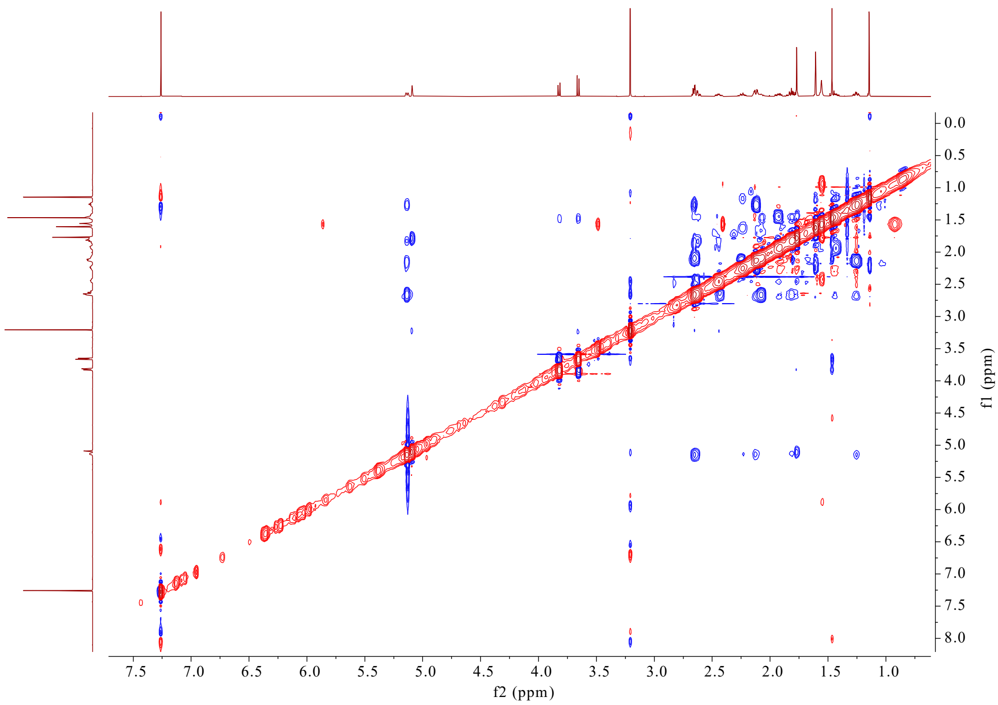

Figure S73. NOESY spectrum (600 MHz) of compound 7 in $CDCl_3$.

MS spectra

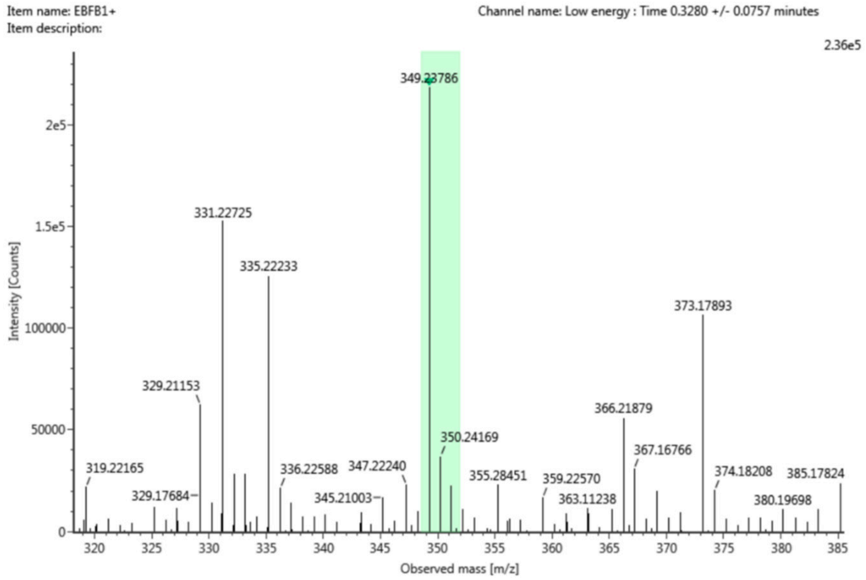

Formula Calculator Results

Formula	Neutral mass (Da)	Observed m/z	Observed RT (min)	Mass error (mDa)	Mass error (ppm)	Adducts
C21H32O4	348.23006	349.2379	0.33	0.5	1.5	+H

Figure S74. HR-ESI-MS spectrum of compound 7.

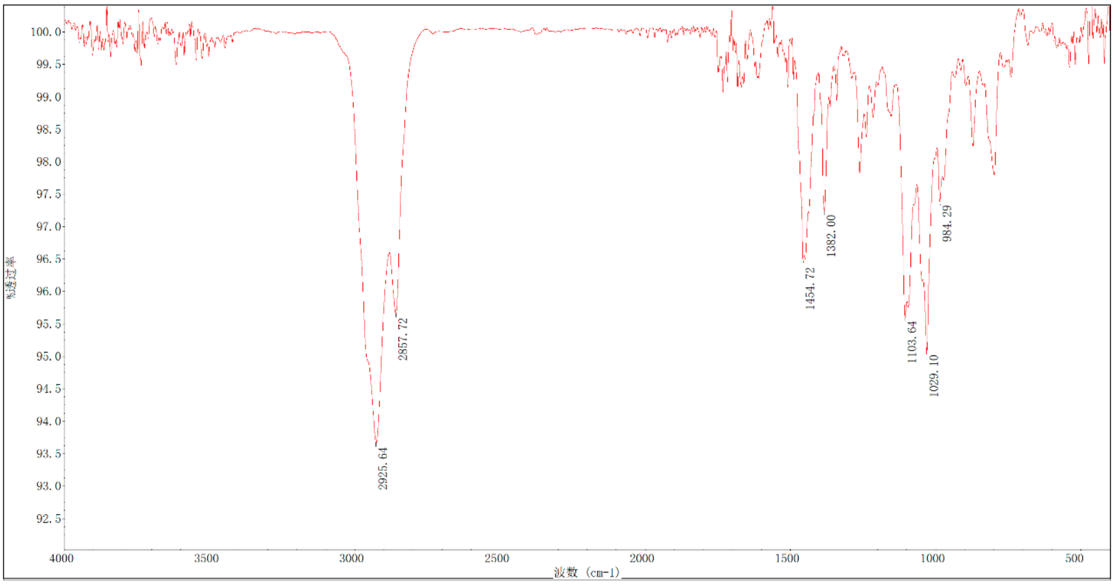

Figure S75. IR spectrum of compound 7.

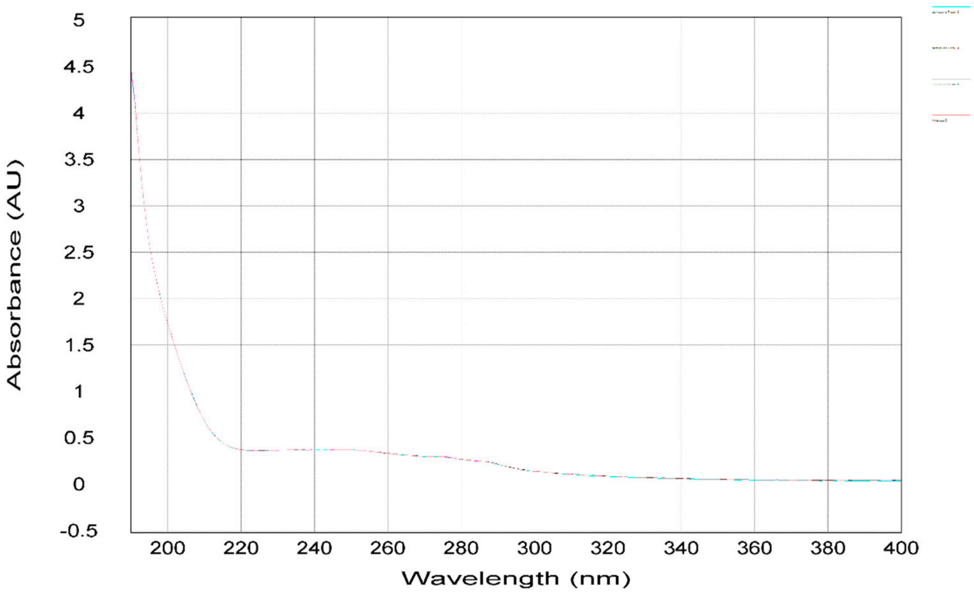

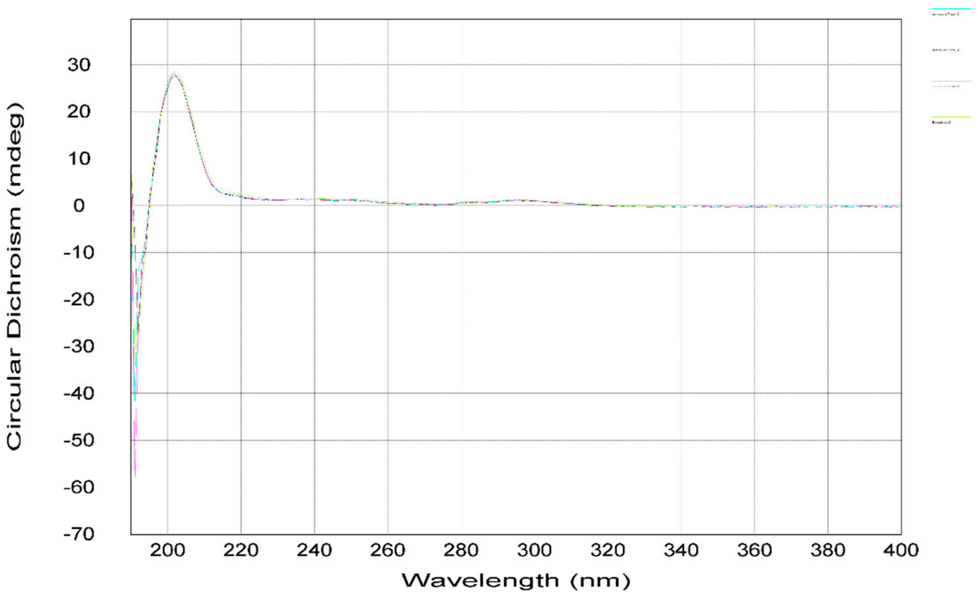

Figure S76. UV and CD spectrum of compound 7.

Functional	Solvent?	Basis Set	Type of Data
mPW1PW91	PCM	6-311+G(d,p)	Shielding Tensors

	Isomer 1	Isomer 2	Isomer 3	Isomer 4	Isomer 5	Isomer 6
sDP4+ (H data)	0.00%	0.00%	0.00%	100.00%	-	-
sDP4+ (C data)	0.00%	0.00%	0.00%	100.00%	-	-
sDP4+ (all data)	0.00%	0.00%	0.00%	100.00%	-	-
uDP4+ (H data)	0.00%	0.00%	0.00%	100.00%	-	-
uDP4+ (C data)	0.00%	0.00%	0.00%	100.00%	-	-
uDP4+ (all data)	0.00%	0.00%	0.00%	100.00%	-	-
DP4+ (H data)	0.00%	0.00%	0.00%	100.00%	-	-
DP4+ (C data)	0.00%	0.00%	0.00%	100.00%	-	-
DP4+ (all data)	0.00%	0.00%	0.00%	100.00%	-	-

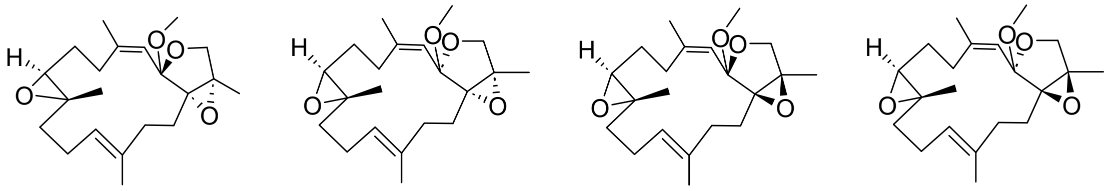

Isomer 1 Isomer 2 Isomer 3 Isomer 4

Figure S77. DP4+ results of compound 7 (Isomer 1: 1*S, 2*R**, 7*S**, 8*S**, 15*S**; Isomer 2: 1*S**, 2*S**, 7*S**, 8*S**, 15*S**; Isomer 3: 1*R**, 2*R**, 7*S**, 8*S**, 15*R**; Isomer 4: 1*R**, 2*S**, 7*S**, 8*S**, 15*R**).**

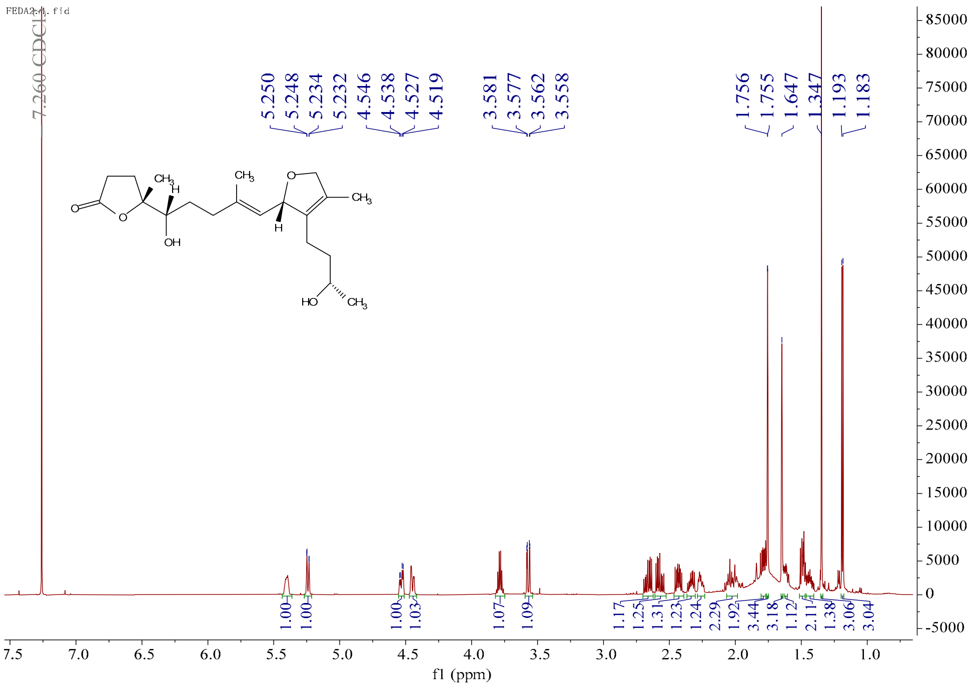

Figure S78. ^{1}H NMR spectrum (600 MHz) of compound 8 in $CDCl_3$.

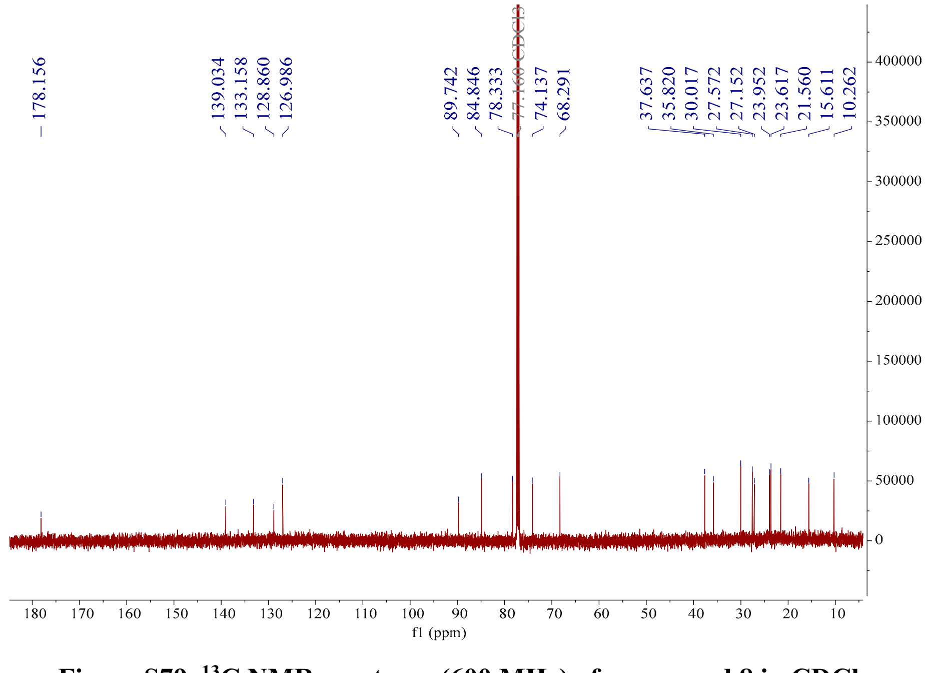

Figure S79. ^{13}C NMR spectrum (600 MHz) of compound 8 in $CDCl_3$.

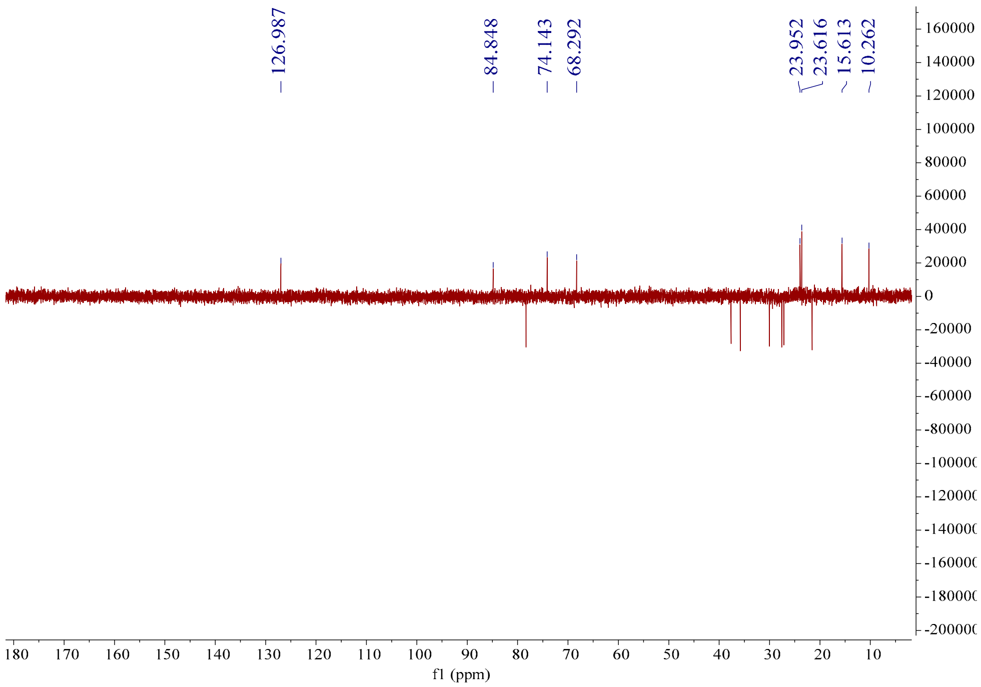

Figure S80. DEPT spectrum (150 MHz) of compound 8 in $CDCl_3$.

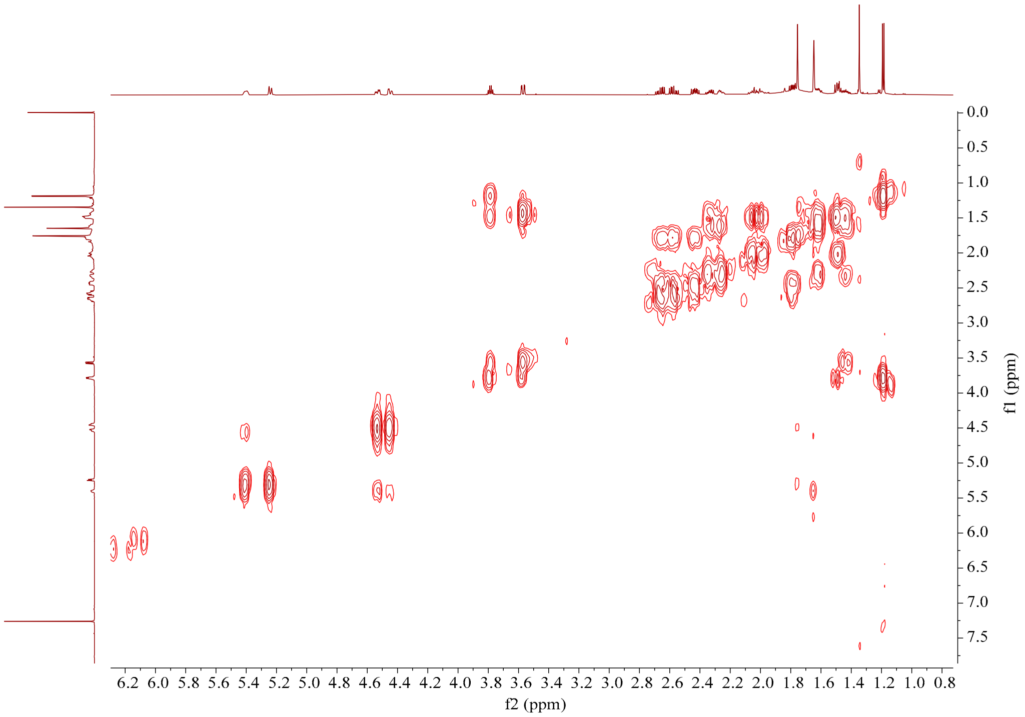

Figure S81. ^{1}H-^{1}H COSY spectrum (600 MHz) of compound 8 in $CDCl_3$.

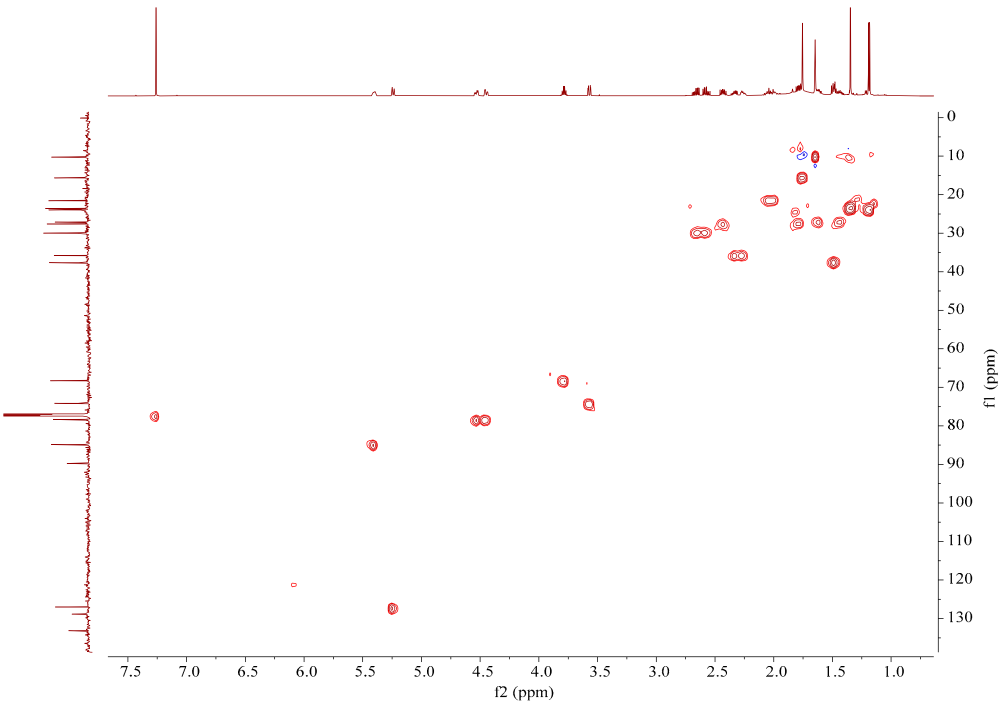

Figure S82. HSQC spectrum (600 MHz) of compound 8 in CDCl3.

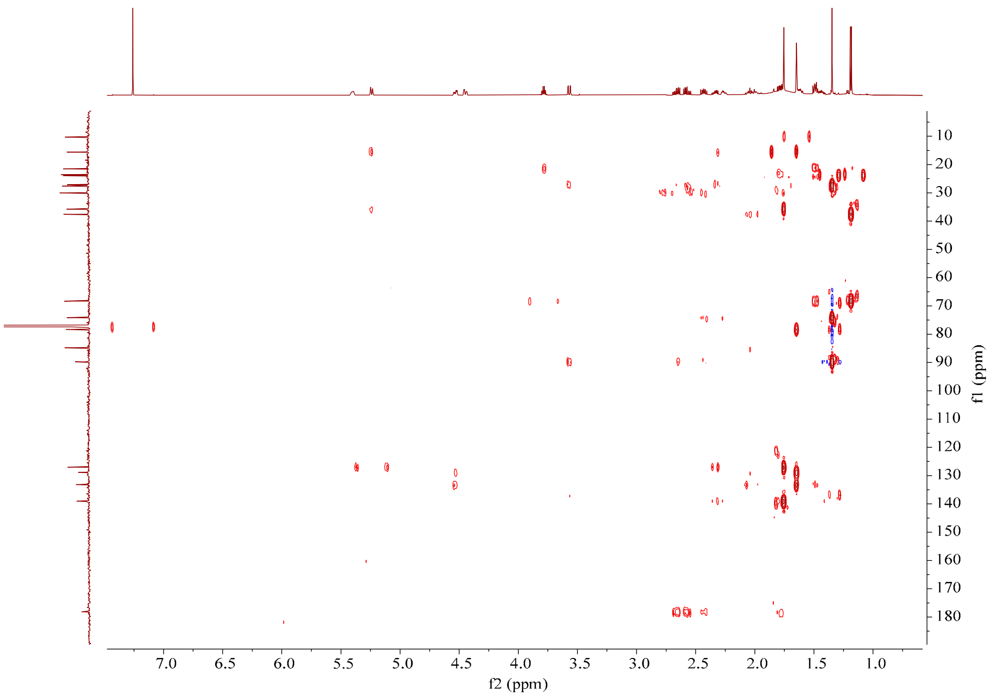

Figure S83. HMBC spectrum (600 MHz) of compound 8 in CDCl3.

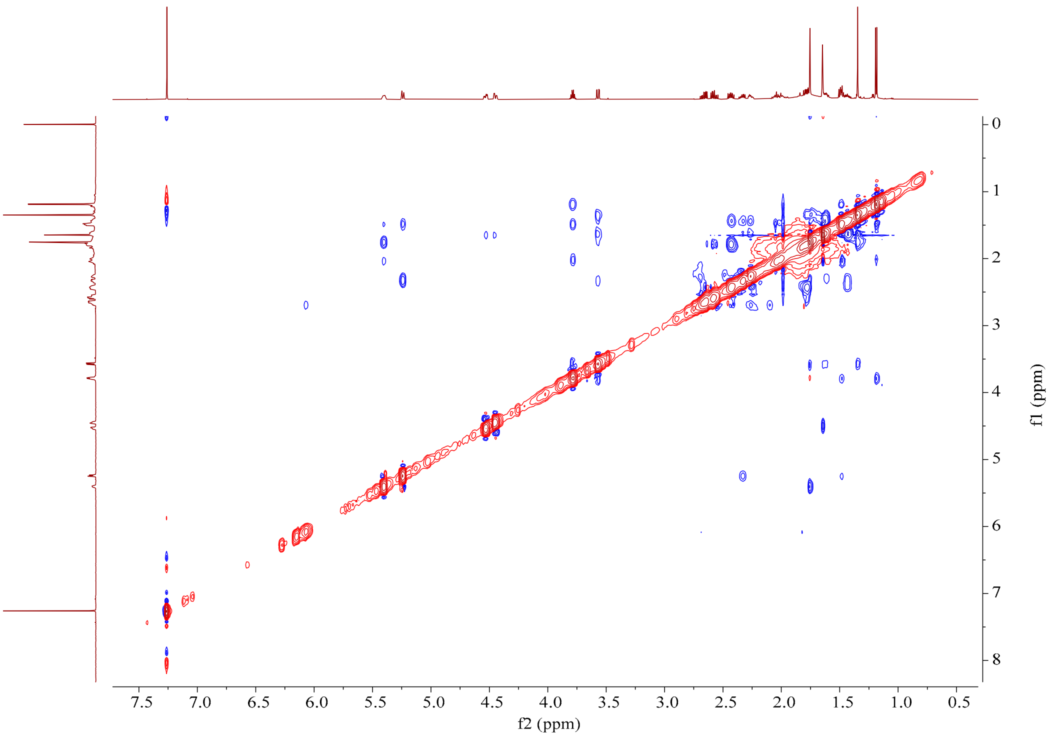

Figure S84. NOESY spectrum (600 MHz) of compound 8 in $CDCl_3$.

MS spectra

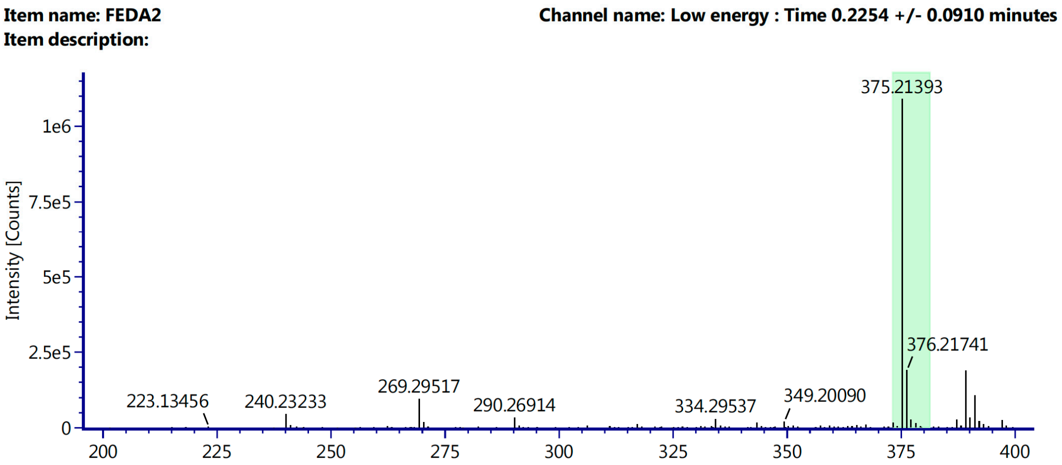

Formula Calculator Results

Formula	Neutral mass (Da)	Observed m/z	Observed RT (min)	Mass error (mDa)	Mass error (ppm)	Adducts
C20H32O5	352.22497	375.2139	0.23	-0.3	-0.7	+Na

Figure S85. HR-ESI-MS spectrum of compound 8.

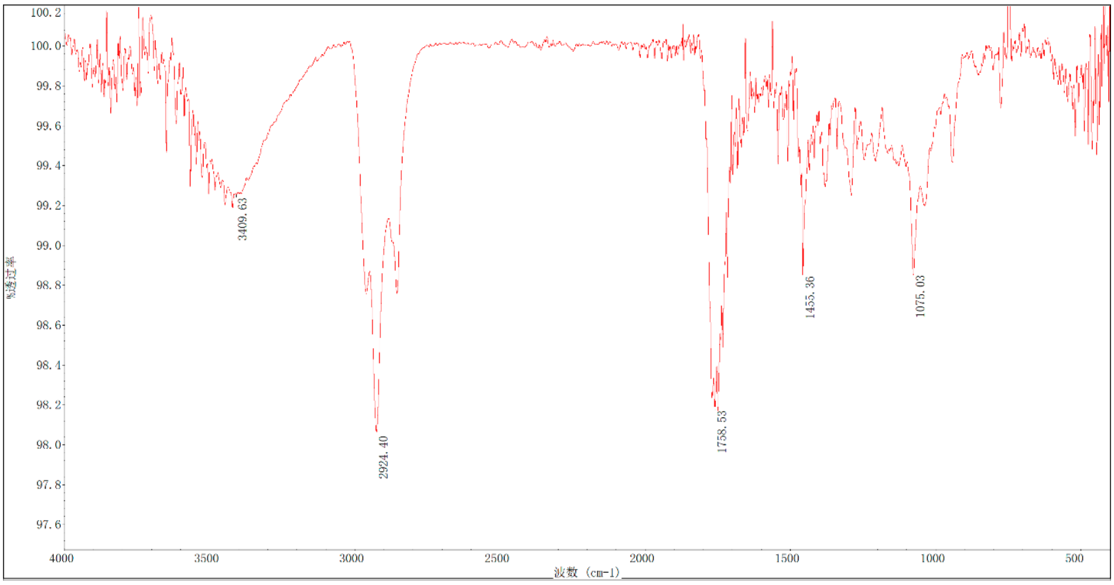

Figure S86. IR spectrum of compound 8.

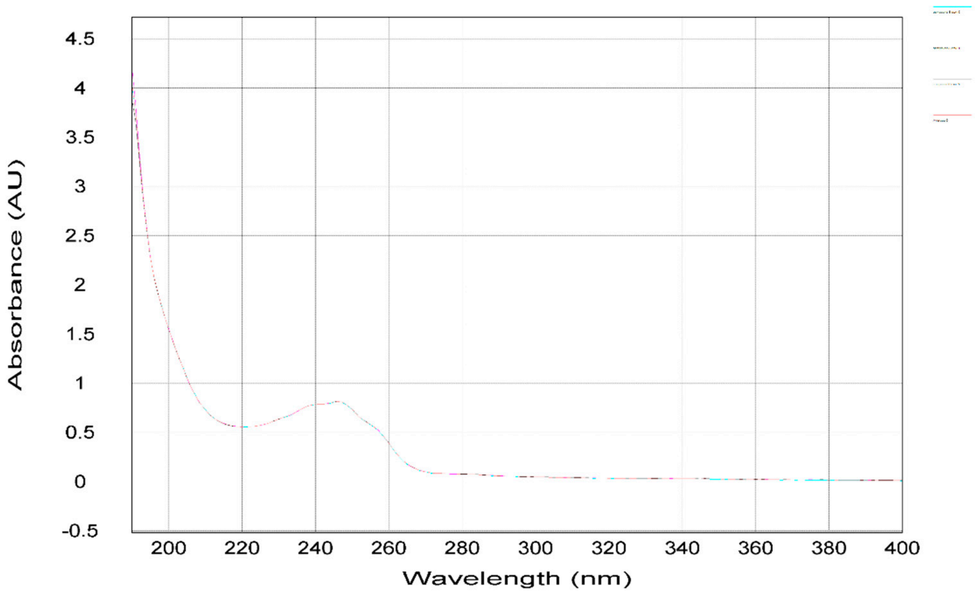

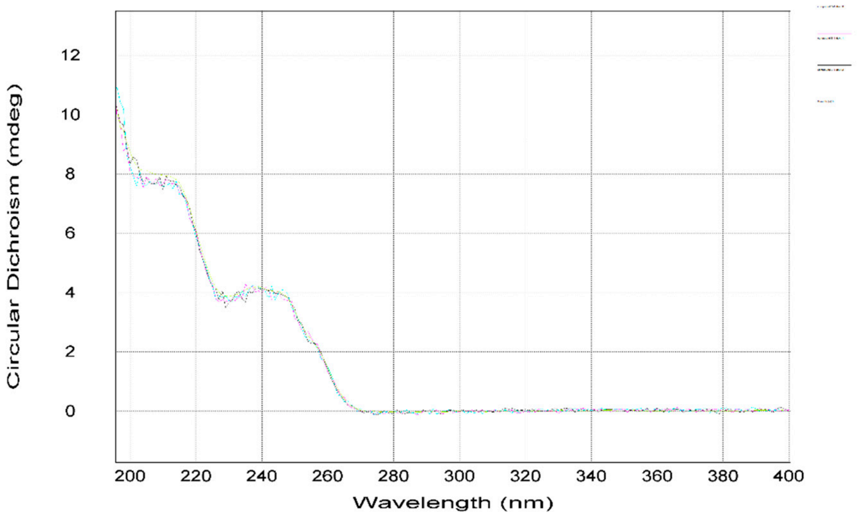

Figure S87. UV and CD spectrum of compound 8.

Functional	Solvent?	Basis Set	Type of Data			
mPW1PW91	PCM	6-311+G(d,p)	Shielding Tensors			
	Isomer 1	Isomer 2	Isomer 3	Isomer 4	Isomer 5	Isomer 6
sDP4+ (H data)	0.23%	99.77%	0.00%	0.00%	-	-
sDP4+ (C data)	3.92%	96.08%	0.00%	0.00%	-	-
sDP4+ (all data)	0.01%	99.99%	0.00%	0.00%	-	-
uDP4+ (H data)	1.20%	98.80%	0.00%	0.00%	-	-
uDP4+ (C data)	25.20%	74.24%	0.56%	0.00%	-	-
uDP4+ (all data)	0.41%	99.59%	0.00%	0.00%	-	-
DP4+ (H data)	0.00%	100.00%	0.00%	0.00%	-	-
DP4+ (C data)	1.37%	98.63%	0.00%	0.00%	-	-
DP4+ (all data)	0.00%	100.00%	0.00%	0.00%	-	-

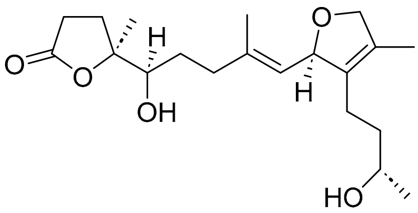

Isomer 1

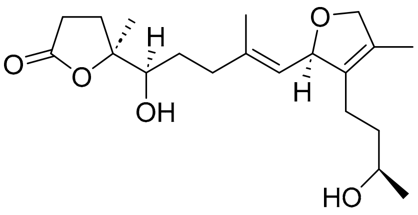

Isomer 2

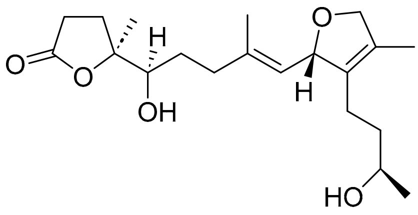

Isomer 3

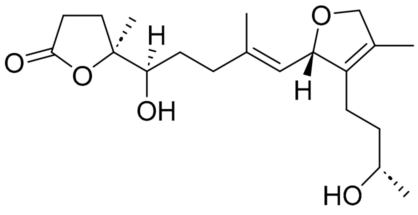

Isomer 4

Figure S88. DP4+ results of compound 8 (Isomer 1: 2*R, 7*R**, 8*S**, 12*S**; Isomer 2: 2*R**, 7*R**, 8*S**, 12*R**; Isomer 3: 2*S**, 7*R**, 8*S**, 12*R**; Isomer 4: 2*S**, 7*R**, 8*S**, 12*S**).**
